# Molecular diversity and evolution of far-red light-acclimated photosystem I

**DOI:** 10.3389/fpls.2023.1289199

**Published:** 2023-11-20

**Authors:** Christopher J. Gisriel, Donald A. Bryant, Gary W. Brudvig, Tanai Cardona

**Affiliations:** ^1^ Department of Chemistry, Yale University, New Haven, CT, United States; ^2^ Department of Biochemistry and Molecular Biology, The Pennsylvania State University, University Park, PA, United States; ^3^ Department of Molecular Biophysics and Biochemistry, Yale University, New Haven, CT, United States; ^4^ Department of Life Sciences, Imperial College London, London, United Kingdom; ^5^ School of Biological and Behavioural Sciences, Queen Mary University of London, London, United Kingdom

**Keywords:** far-red light, photosystem I, photosynthetic diversity, molecular evolution, chlorophyll f, ancestral sequence reconstruction, phylogenetic analysis, structural analysis

## Abstract

The need to acclimate to different environmental conditions is central to the evolution of cyanobacteria. Far-red light (FRL) photoacclimation, or FaRLiP, is an acclimation mechanism that enables certain cyanobacteria to use FRL to drive photosynthesis. During this process, a well-defined gene cluster is upregulated, resulting in changes to the photosystems that allow them to absorb FRL to perform photochemistry. Because FaRLiP is widespread, and because it exemplifies cyanobacterial adaptation mechanisms in nature, it is of interest to understand its molecular evolution. Here, we performed a phylogenetic analysis of the photosystem I subunits encoded in the FaRLiP gene cluster and analyzed the available structural data to predict ancestral characteristics of FRL-absorbing photosystem I. The analysis suggests that FRL-specific photosystem I subunits arose relatively late during the evolution of cyanobacteria when compared with some of the FRL-specific subunits of photosystem II, and that the order Nodosilineales, which include strains like *Halomicronema hongdechloris* and *Synechococcus* sp. PCC 7335, could have obtained FaRLiP via horizontal gene transfer. We show that the ancestral form of FRL-absorbing photosystem I contained three chlorophyll *f*-binding sites in the PsaB2 subunit, and a rotated chlorophyll *a* molecule in the A_0B_ site of the electron transfer chain. Along with our previous study of photosystem II expressed during FaRLiP, these studies describe the molecular evolution of the photosystem complexes encoded by the FaRLiP gene cluster.

## 1 Introduction

Far-Red Light Photoacclimation (FaRLiP) enables a subset of cyanobacteria to perform oxygenic photosynthesis using far-red light (FRL, 700-800 nm) (Gan and Bryant, 2015; Ho et al., 2017; Nürnberg et al., 2018; Ho et al., 2020). When the cyanobacteria are grown in environments that are enriched in FRL, a gene cluster is upregulated that contains ~20 genes: a PsbA paralogue called ChlF that enables the synthesis of chlorophyll (Chl) *f*, several photosystem I (PSI) and photosystem II (PSII) subunits, several phycobiliproteins, and a phytochrome and response regulator system. For PSI and PSII, this results in the alteration of some Chl-binding sites and the incorporation of FRL-absorbing Chls *d* and *f* in addition to the standard Chl *a*, allowing them to perform the light reactions of photosynthesis using the lower energy FRL. Recently, FRL-absorbing PSI and PSII (FRL-PSI and FRL-PSII, respectively) have been investigated using various spectroscopic and structural techniques (Nürnberg et al., 2018; Hastings et al., 2019; Zamzam et al., 2019; Cherepanov et al., 2020; Gisriel et al., 2020; Judd et al., 2020; Kato et al., 2020; Tros et al., 2020; Zamzam et al., 2020; Gisriel et al., 2021; Gisriel et al., 2022a; MacGregor-Chatwin et al., 2022; Viola et al., 2022; Gisriel et al., 2022b; Gisriel et al., 2022c, ; Gisriel et al., 2023b). In FRL-PSII, the PsbA (i.e., D1), PsbB (i.e., CP47), PsbC (i.e., CP43), PsbD (i.e., D2), and PsbH subunits are all FRL-specific. The Chl_D1_ site in the electron transfer chain (ETC) is occupied by a Chl *d* molecule, and four Chl *f* molecules are found at high occupancy in specific sites of the PsbB and PsbC core antenna subunits (Gisriel et al., 2022a; Gisriel et al., 2022c; Gisriel et al., 2023b). In FRL-PSI, the PsaA, PsaB, PsaF, PsaJ, PsaI, and PsaL subunits are FRL specific, and at least five of the antenna Chl sites are highly occupied by Chl *f* (Gisriel et al., 2020; Gisriel et al., 2021; Gisriel et al., 2022b). Note that the FRL-specific isoforms of those subunits are called PsaA2, PsaB2, PsaF2, PsaJ2, PsaI2, and PsaL2, respectively.

**Figure 1:**
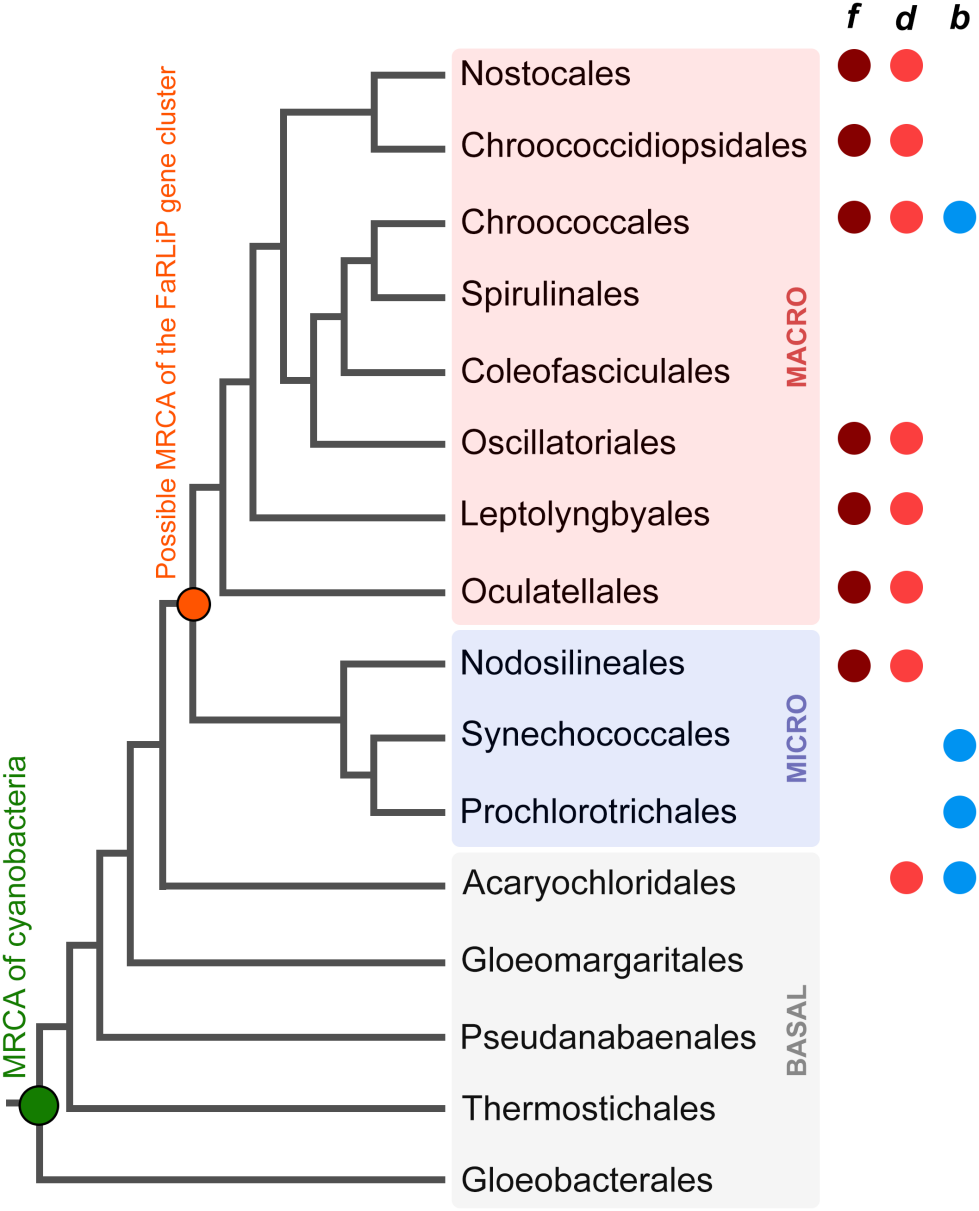
Evolutionary relationships of cyanobacteria. A schematic tree based on that presented by Strunecký et al., 2023 highlighting some of the key orders as proposed by the authors. BASAL (gray shading) denotes the early diversification events, and MICRO (pale blue shading) and MACRO (pale pink shading) denote microcyanobacteria and macrocyanobacteria, respectively, as described previously (Sánchez-Baracaldo, 2015; Boden et al., 2021). The colored circles represent the presence of strains with the capacity to produce Chl *f*, *d*, or *b* within the respective order. The green circle marks the most recent common ancestor (MRCA) of all cyanobacteria and the orange circle marks the MRCA of all organisms that have inherited a complete FaRLiP gene cluster assuming this has been inherited vertically since its formation. The tree recapitulates only branching orders, but branch lengths do not represent in this instance phylogenetic distance.

The FaRLiP response is just one example of acclimation and adaptation mechanisms that occur in the phylum Cyanobacteria, which include a large diversity of organisms distributed across nearly all environments (Ho et al., 2017). A schematic and simplified representation of cyanobacterial phylogeny is shown in **Figure 1** based on the recent taxonomic work by Strunecký et al., 2023, and some representative genera and strains are listed in **Table 1**. After the early diversification events leading to the basal clades, a large expansion of diversity occurred, which can be subdivided in two major radiation events; one leading to the microcyanobacteria and another to the macrocyanobacteria, as described previously (Sánchez-Baracaldo, 2015; Boden et al., 2021). The microcyanobacteria are characterized by having an overall small cell diameter, ranging from ca. 1 to 2 μm, while the macrocyanobacteria are characterized by having overall larger cell diameters in the range of 3 to 50 μm.

**Table 1:** Overall classification of cyanobacteria highlighting clades of relevance for this study.

Tree placement	Orders	Example of strains
Basal	Gloeobacterales	*Gloeobacter violaceus*, *Anthocerotibacter panamensis*
	Thermostichales	*Synechococcus* (*Thermosticus*) sp. JA-3Ab, *Synechococcus* sp PCC 7336
	Pseudanabaenales	*Pseudanabaena* sp. PCC6802*, Synechococcus* sp. PCC 7502
	Acaryochloridales	*Acaryochloris marina, Thermosynechococcus elongatus, Cyanothece* sp. PCC 7425*, Synechococcus* sp. PCC 6312
Microcyanobacteria	Nodosilineales	*Synechococcus* sp. PCC 7335, *Halomicronema hongdechloris, Nodosilinea nodulosa*, *Leptolyngbya* sp. PCC 6406
	Prochlorotrichales	*Prochlorothrix hollandica*
	Synechococcales	*Cyanobium gracile*, *Synechococcus* sp. RS9916, *Synechococcus elongatus*, *Prochlorococcus marinus*
Macrocyanobacteria	Leptolyngbyales	Oscillatoriales cyanobacterium JSC-12, *Leptolyngbya boryana*, *Stenomitos frigidus*
	Oscillatoriales	*Lyngbya* sp. PCC 8106, *Oscillatoria nigro-viridis*, *Planktothrix rubescens*
	Chrooccocales	*Synechocystis* sp. PCC 6803, *Microcystis aeruginosa*, *Prochloron didemni*, *Pleurocapsa* sp. PCC 7319
	Chroococcidiopsidales	*Gloeocapsa* sp. PCC 7428, *Chroococcidiopsis thermalis*
	Nostocales	*Nostoc punctiforme*, *Fischerella* sp. PCC 9432, *Calothrix* sp. PCC 6303, *Scytonema hofmannii*

FaRLiP is well distributed in the microcyanobacteria and macrocyanobacteria but has not yet been discovered in a basal clade (Antonaru et al., 2020). FaRLiP strains are found in a variety of environments including freshwater, terrestrial, marine, or host associated (Antonaru et al., 2020; Chen et al., 2021). A few other known adaptations to FRL occur in cyanobacteria: within the basal clade Acaryochloridales, in which some strains constitutively express Chl *d* (Chen et al., 2021; Viola et al., 2022), and in some *Thermostichus* (formerly *Synechococcus*) spp. in which unique phycobiliproteins absorb FRL and transfer energy to PSI, as part of a photoacclimation to low light (LoLiP) (Nowack et al., 2015; Olsen et al., 2015; Soulier et al., 2020; Soulier and Bryant, 2021; Gisriel et al., 2023a).

The origin and early evolution of photosynthesis has remained challenging to study because many of the key innovations during the emergence of pigment biosynthesis and the photosystems occurred in deep time, obscuring transitional stages and patterns of gene loss and gain (Cardona, 2019). Given that FaRLiP evolved within the cyanobacteria, and the process involves a well-defined set of genes, it provides a more tractable opportunity to study how adaptations in photosynthesis, requiring the synthesis of new pigments and photosystem subunits, can emerge and are distributed in nature. Advances in cyanobacterial genomics have also enabled studies of this process in detail. A study by Antonaru et al., 2020 surveyed genomes and metagenome projects for cyanobacteria that can perform FaRLiP, and based on the phylogeny of ApcE2, a phycobilisome linker subunit expressed during FaRLiP, the authors suggested that the FaRLiP gene cluster had been inherited vertically from the most recent common ancestor of microcyanobacteria and macrocyanobacteria (**Figure 1**). This implies widespread loss of FaRLiP capability among a large diversity of cyanobacteria. In that study, no evidence for the horizontal transfer of a full FaRLiP gene cluster was observed.

We previously studied the molecular evolution of FRL-PSII (Gisriel et al., 2022a) and observed a sequential emergence of FaRLiP components based on phylogenetic inference. First, ChlF (Ho et al., 2016; Shen et al., 2019; Trinugroho et al., 2020) originated from a duplication event, which seemed to antedate the most recent common ancestor (MRCA) of cyanobacteria (Cardona et al., 2015). At what point in its evolutionary history this divergent PsbA acquired its novel pigment synthesis function is still unclear, but its early divergence opens the possibility of ancestral states prior to the emergence of FaRLiP specific photosystem subunits utilizing Chl *f* or *d* in the absence of any other photosystem change, which in PSI have experimentally been shown to be functional (Kurashov et al., 2019). Second, we saw that the FRL-specific PsbA incorporated into FRL-PSII was also amongst the oldest of the FRL-PSII subunits, possibly diverging shortly before the MRCA of cyanobacteria. The duplication leading to this subunit enabled the evolution of a specific binding site for Chl *d* at position Chl_D1_ (Nürnberg et al., 2018; Gisriel et al., 2022c; Gisriel et al., 2023b), likely leading to an enhancement of the yield of charge separation under FRL. Third, the evolution of FRL-specific PsbA was followed by duplications leading to the FRL-specific PsbD and PsbC subunits, both of which had a basal position in their respective phylogenetic trees, splitting shortly after cyanobacteria began to diversify. The duplication of these subunits likely led to optimization of FRL absorbance, energy transfer, and electron transfer. Finally, we observed duplications leading to FRL-specific PsbB and PsbH that occurred along with the diversification of known groups of cyanobacteria, which further optimized FRL harvesting by adding additional Chl *f* binding sites and altering the linkage to the FRL-specific phycobilisome (Gisriel et al., 2023b).

The in-depth evolutionary analyses of FRL-PSII let to unexpected insights, but the molecular evolution of FRL-PSI has not been analyzed. Here, we explore the molecular evolution of FRL-PSI using phylogenetic and structural approaches. We show that unlike FRL-PSII, FRL-PSI paralogs retained in the FaRLiP gene cluster have a relatively late origin and likely emerged in an ancestor of the macrocyanobacteria or within their diversity. This suggests that microcyanobacteria capable of FaRLiP (Nodosilineales: *Synechococcus* 7335, *Halomicronema*, etc.) acquired their FRL-PSI paralogs at a late stage via a horizontal gene transfer (HGT). In the context of sequence conservation and ancestral sequence reconstructions, we show that the ancestral FRL-PSI had three Chl sites that bound Chl *f* with high specificity. We also reveal that all available FRL-PSI structures contain a slight rotation of the Chl *a* in the A_0B_ site of the ETC due to nearby FRL-specific residues, which has gone unnoticed in recent structural reports. This A_0B_ Chl rotation was most likely also present in the ancestral form of FRL-PSI.

## 2 Materials and methods

### 2.1 Construction of phylogenetic trees and ancestral sequences

Amino acid sequences for PSI subunits were downloaded from the NCBI refseq database using PSI-BLAST restricted to the phylum Cyanobacteria. PsaA, PsaA2, PsaB, and PsaB2 sequences were downloaded on the 17^th^ of August 2021, PsaL(2) sequences were downloaded on the 14^th^ of February 2022, and PsaJ, PsaJ2, PsaI, PsaI2, PsaF, and PsaF2 sequences were downloaded on the 2^nd^ of May 2022. The sequences were curated to remove fragmented sequences and to decrease sequence redundancy to 98% identity. The following numbers of sequences remained: 467 PsaA (38 PsaA2), 527 PsaB (38 PsaB2), 708 PsaF (54 PsaF2), 696 PsaJ (54 PsaJ2), 630 PsaI (44 PsaI2), and 900 PsaL (50 PsaL2).

To acquire outgroups for PsaI and PsaJ, we examined *Gloeobacter* spp. sequences. Genomic studies of *Gloeobacter* and their close relatives (Gloeobacterales) have suggested that their photosystems lack several of the subunits commonly found in other cyanobacteria. In the case of PSI, it had previously been reported that Gloeobacterales uniquely lack PsaI, PsaJ and PsaK (Nakamura et al., 2003), and instead had an additional subunit known as PsaZ (Inoue et al., 2004). However, the cryo-electron microscopy structure of *Gloeobacter violaceus* PSI revealed that PsaZ occupies the same position and has the same fold as PsaI (Kato et al., 2022). Given the short sequence and the large phylogenetic distance of Gloeobacterales to all other cyanobacteria, the level of sequence identity with well described PsaI (e.g., *Thermosynechococcus vulcanus*) is low, at just over 20% (9 of 40 identical residues in alignment) compared with 62% (25/40) for *T. vulcanus vs*. *Synechocystis* sp. PCC 6803, and 45% (18/40) when comparing *T. vulcanus* PsaI with a PsaI2 sequence. It is safe to assume that PsaZ is indeed PsaI, and hereafter we will refer to it as such. We found that the *psaI* (*psaZ*) gene was only annotated in the genome of *Gloeobacter kilaueensis*, but it was missing in the genomes of other Gloeobacterales. A tblastn (protein to translated nucleotide BLAST), using the *G. kilaueensis* PsaI amino acid sequence retrieved the homologous sequence from unannotated genes in *G. violaceus*, *G. morelensis*, and *Anthocerotibacter panamensis*. These were added to the sequence alignment of PsaI as an outgroup. Similarly, the structure of PSI from *G. violaceus* also revealed a subunit at the exact position of PsaJ, which was modeled as a chain of alanine residues. We assumed that the gene encoding PsaJ might be in the genome but had not been identified by automated annotation pipelines. Indeed, the unannotated *psaJ* gene was found next to *psaF* in the genomes of the three *Gloeobacter* strains or annotated as a hypothetical gene in the genomes of *Anthocerotibacter panamensis* and *Candidatus* Cyanaurora vandensis. These were also added to the PsaJ/PsaJ2 sequence alignment as outgroups.

The sequences were aligned with Clustal Omega using five combined guided trees and HMM iterations (Sievers et al., 2011). Maximum likelihood phylogenetic analysis was done with IQTREE multicore version 2.0.3 (Minh et al., 2020) and run in a computing cluster. The best substitution model was selected with ModelFinder (Kalyaanamoorthy et al., 2017) and support values were calculated using ultrafast bootstrap with >1000 iterations until the correlation coefficient converged and also using the average likelihood ratio test method. Ancestral sequence reconstruction was carried out by activating the function *-asr* on IQTREE. Trees were visualized with the software Dendroscope version 3.8.1 (Huson and Scornavacca, 2012). Sequence alignments, trees, and inferred ancestral sequences with posterior probabilities for the FaRLiP ancestor as well as all other nodes in the trees are provided in **Supplementary Data 1**.

### 2.2 Structural comparisons

To make structural comparisons, the following coordinate files for corresponding structures were gathered from the Protein Data Bank (PDB): FRL-PSI from *Fischerella thermalis* PCC 7521 (PDB 7LX0) (Gisriel et al., 2020; Gisriel et al., 2021), FRL-PSI from *H. hongdechloris* (PDB 6KMX) (Kato et al., 2020), FRL-PSI from *Synechococcus* sp. PCC 7335 (PDB 7S3D) (Gisriel et al., 2022b), visible light (VL)-PSI from *H. hongdechloris* expressed when the cells are grown in VL (PDB 6KMW) (Kato et al., 2020), and PSI from the non-FaRLiP cyanobacteria *Thermosynechococcus elongatus* (PDB 1JB0) (Jordan et al., 2001) and *Synechocystis* sp. PCC 6803 (PDB 5OY0) (Malavath et al., 2018). Structures were superimposed using the *super* function of PyMOL (DeLano, 2014). FRL-specific residues were determined based on multiple sequence alignments and highlighted on the structure of FRL-PSI from *F. thermalis* PCC 7521.

## 3 Results

### 3.1 Phylogenetic overview

**Figure 2:**
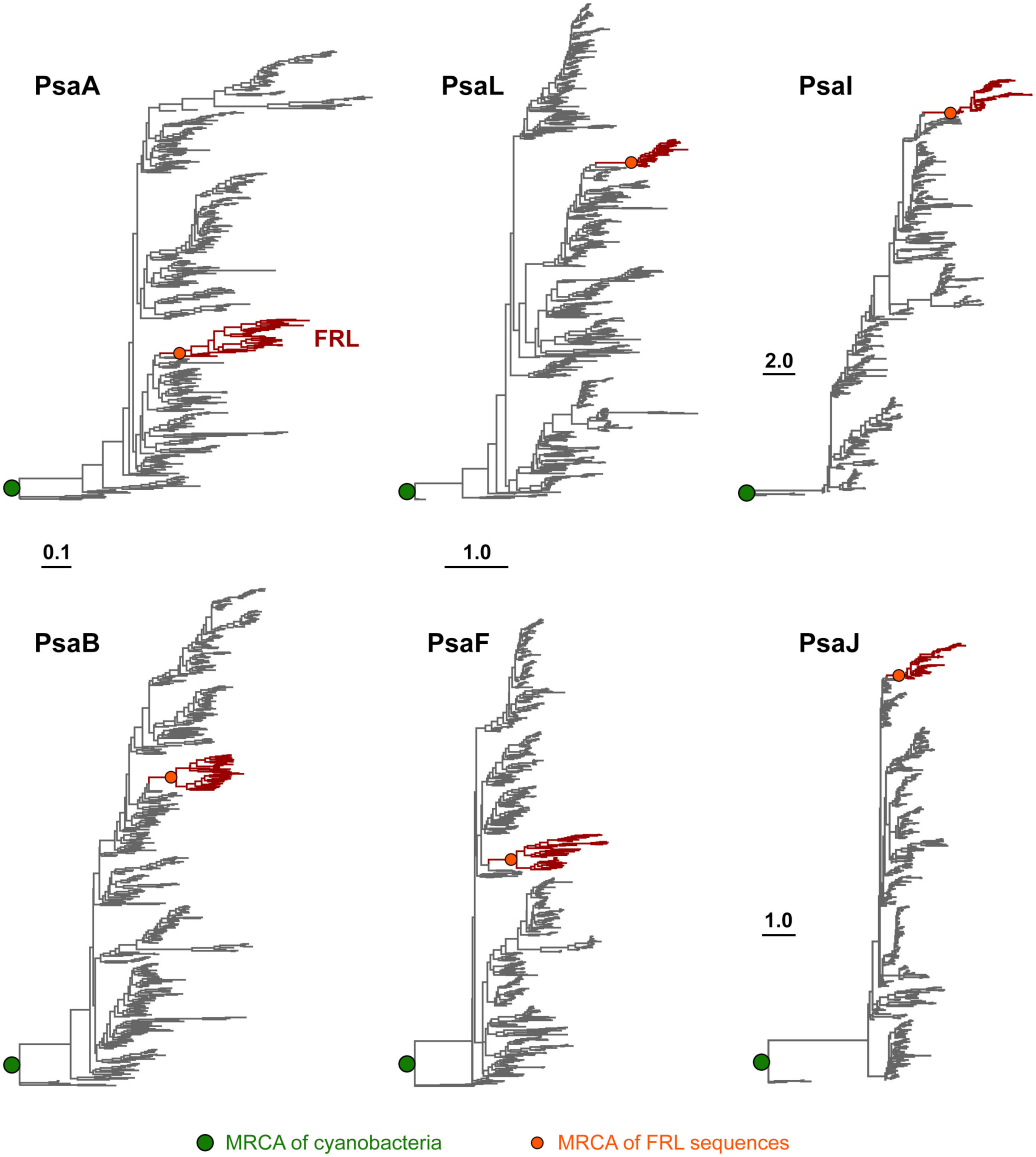
Maximum Likelihood phylogenetic trees of PSI subunits. Each tree was rooted placing sequences from the Gloeobacterales as an outgroup. Branches in gray represent VL subunits, while those in red show the FRL subunits. Scale bars denote amino acid substitutions per site, for PsaA and PsaB, PsaL and PsaF, PsaI, and PsaJ, respectively.

**Figure 3:**
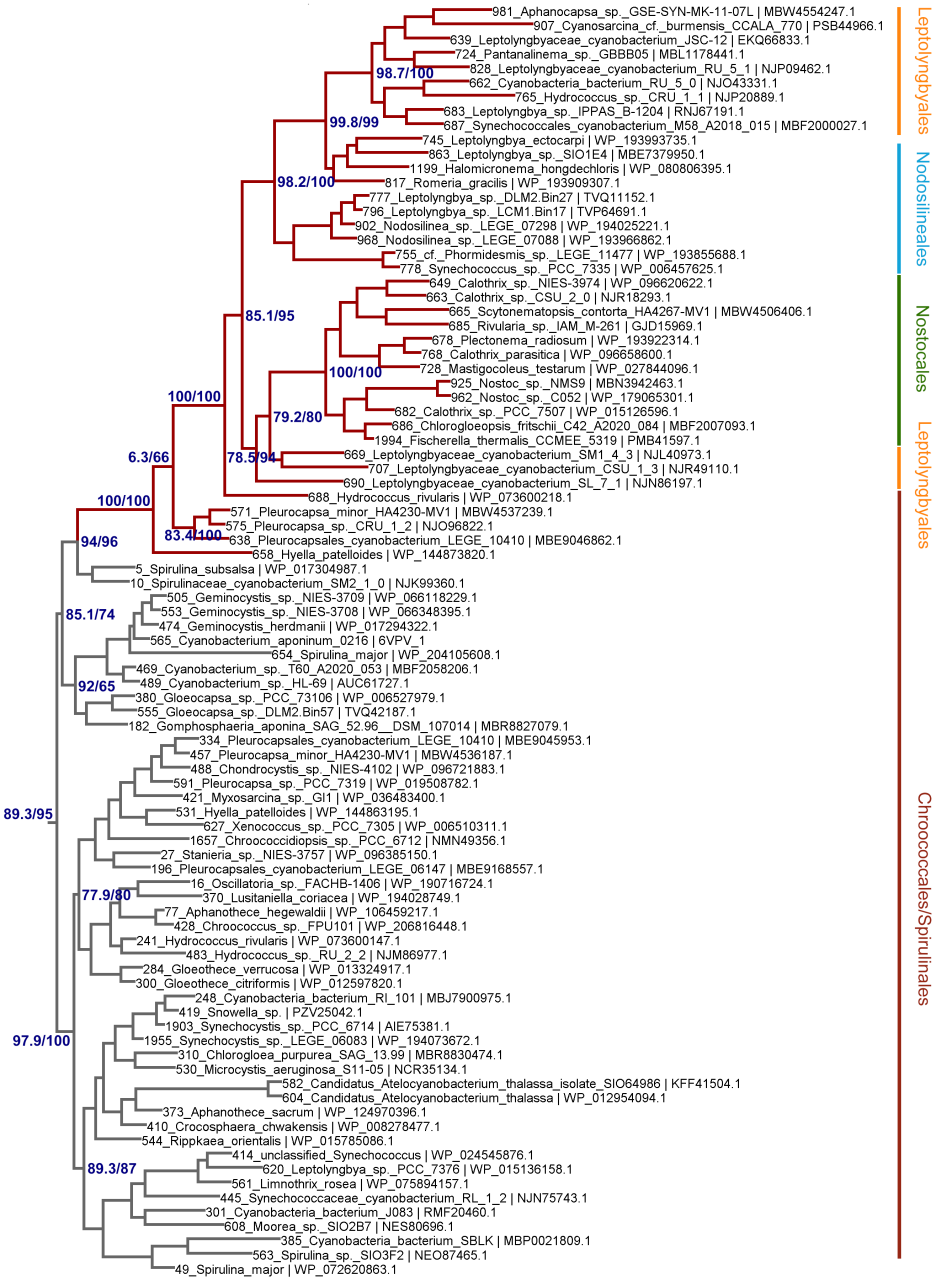
Close-up on the Maximum Likelihood (ML) phylogeny of PsaA around the PsaA2 sequences (dark red branches). Grey branches represent the visible light (VL) form. Orders are shown to the right. Support values are by the Ultrafast Bootstrap and the Average Likelihood Ratio Test methods, respectively, and these are only shown on selected branches for clarity. The complete tree is found in the **Supplementary Data 1**.

**Figure 4:**
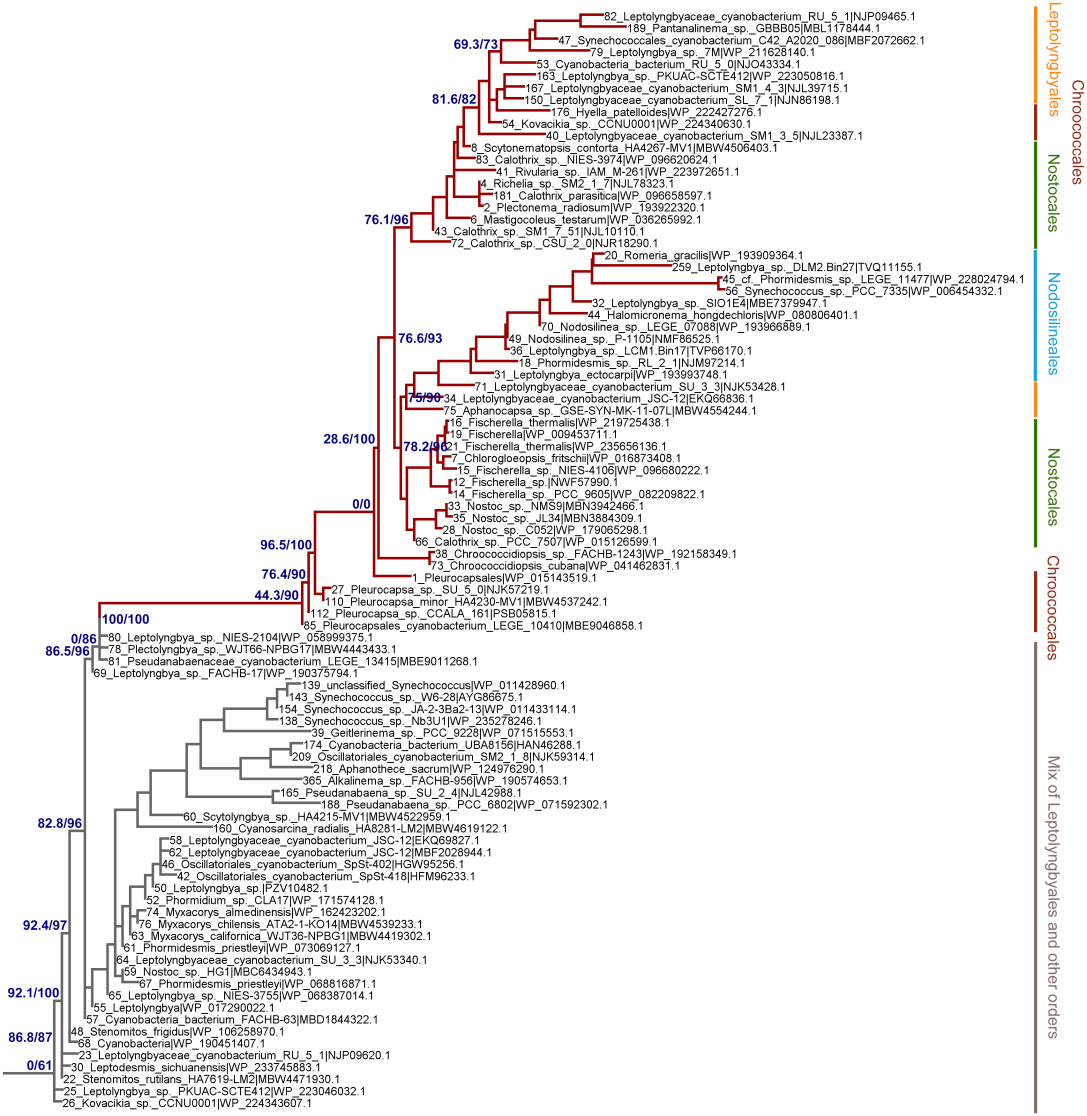
Close-up on the ML phylogeny of PsaI around the PsaI2 sequences (dark red branches). Grey branches represent the VL forms, which in this case are not well resolved and show mostly sequences belonging to the Leptolyngbyales mixed with some Pseudanabaenales and Thermostichales sequences. Orders are shown to the right. Support values are by the Ultrafast Boostrap and the Average Likelihood Ration Test methods, respectively, and these are only shown on selected branches for clarity. The complete tree is found in the **Supplementary Data 1**.

To assess how the FRL-PSI subunits evolved relative to the VL paralogs, we performed Maximum Likelihood phylogenetic analysis for sequence alignments of all PSI subunits with FRL variants. We found that none of the FRL-PSI subunits showed signatures of deep ancestry (**Figure 2**): none appear to antedate the last common ancestor of cyanobacteria, and none appear to make a separate lineage of sequences branching out in between any of the known groups of cyanobacteria. Instead, the FRL-PSI sequences were all embedded within a clade of cyanobacteria, appearing to emerge late relative to the diversification of the known groups. Specifically, the FRL sequences appeared more closely related to VL sequences found in macrocyanobacteria of the orders Chroococcales, Spirulinales, Coleofasciculales, Leptolyngbyales, and Oscillatoriales as classified in Strunecký et al., 2023 (**Figures 3**, **4**, **Supplementary Figures 1–4**). PsaA2 sequences were embedded within a cluster of sequences that included representatives from the Chroococcales and Spirulinales (**Figure 3**). PsaB2 sequences were embedded within a cluster of sequences from representatives of the order Oscillatoriales (**Supplementary Figure 1**), and its topology did not match that of PsaA2. PsaL2 branched within a small group of sequences within the Coleofasciculales and Oscillatoriales (**Supplementary Figure 2**), while PsaF2 clustered within sequences found in Chroococcales (**Supplementary Figure 3**). The phylogenetic trees for PsaI and PsaJ are overall poorly resolved because these are very small, mostly hydrophobic subunits. Nevertheless, PsaI2 and PsaJ2 both clustered within sets of sequences composed mostly of VL forms from representatives of the Leptolyngbyales (**Figure 4**, **Supplementary Figure 4**, respectively).

Given that PsaA2 and PsaB2 are usually encoded within the same operon conserved within the FaRLiP gene cluster, it could be predicted that they co-evolved as the operon was inherited and diversified. However, we observe notable differences between PsaA2 and PsaB2. The topology of the PsaA2 subtree places sequences from Chroococcales strains *Hyella patelloides* and *Pleurocapsa* spp. basally, retaining less FRL-specific amino acid substitutions compared to sequences that diversified later in that group (see also section 3.2.1), which is consistent with the phylogenetic position. In contrast, the topology of the PsaB2 subtree is split into two subgroups that can be well recognized at the sequence level, one including heterocystous cyanobacteria (Nostocales) and four sequences from environmental studies, potentially from various orders, and a second that includes all other sequences. This indicates that the sequences have not remained together throughout the entire diversification process of the FaRLiP gene cluster, and exchange of paralogs may have occurred early during their initial diversification. We also observed that the PsaI2 subtree, like PsaA2, also placed at the base sequences from Chroococcales strains (*Pleurocapsa* spp.). Notably, the most basal branch (sequence 85 in **Figure 4**) has no branch length. This means that effectively this sequence is identical to the predicted ancestral sequence, which is supported by the ancestral sequence reconstruction data, and thus has acquired virtually no change relative to the ancestor, yet not accounting for gaps or insertions.

We also found that none of the FRL subtrees in any of the studied subunits from PSI had a topology that recapitulated entirely the species trees of cyanobacteria. Of note was the position of the Nodosilineales (e.g., *Synechococcus* sp. PCC 7335, *Halomicronema*, *Nodosilinea*). Nodosilineales represents a clade sister to all other orders containing the FaRLiP gene cluster. Thus, under the assumption of vertical descent, it could be predicted that the root of each FRL-specific subtree should be placed at the node that separates Nodosilineales from all other sequences. This prediction was not confirmed in any of the trees of FRL-PSI subunits in this work and none of the trees of FRL-PSII sequences including ChlF, from our previous work. Instead, Nodosilineales sequences clustered late, often, but not exclusively within Chroococcales (**Figures 3**, **4**, **Supplementary Figures 1–4**). This pattern opens the possibility that Nodosilineales obtained their FaRLiP gene cluster via HGT.

A third paralog of PsaB, unrelated to the FaRLiP-specific PsaB2 subunit, was noted in the phylogeny of this subunit; it was apparent because it is widespread within Nostocales, whose PsaB sequences split into two distinct groups (**Supplementary Figure 5**). One group contains what could potentially be considered the standard VL form, and this clade included the majority of the Nostocales sequences (92 out of 134, excluding FRL sequences), which clustered next to their closest non-heterocystous relatives in the Chroococcidiopsidales and Gomontiellales according to the phylogenomic classification of Strunecký et al., 2023. Thus, this clade is somewhat congruent with the species tree of cyanobacteria. The second group, PsaB3, contained the remaining 42 sequences in the heterocystous cyanobacteria, and the topology of the tree showed these clustered among a number of sequences from various orders, but mostly Leptolyngbyales and Oscillatoriales. A similar “third paralog” is not seen in the phylogenetic tree of PsaA. These two groups of PsaB are found together with other sequences belonging to members of the Leptolyngbyales, making up a sister clade to the VL sequences from which the PsaB2 sequence originated.

### 3.2 FRL-specific residues and characteristics of ancestral FRL subunits

#### 3.2.1 Overview of FRL-specific residues

To determine which residues of FRL-PSI subunits are FRL-specific, we constructed a separate multiple sequence alignment of the following subunits: PsaA/PsaA2, PsaB/PsaB2, PsaF/PsaF2, PsaJ/PsaJ2, and PsaL/PsaL2 (**Supplementary Figure 6**). Each included sequences from diverse cyanobacterial species including *F. thermalis* PCC 7521, *H. hongdechloris*, *Synechococcus* sp. PCC 7335, *Aphanocapsa* sp. GSE-SYN-MK-11-07L, *Chroococcidiopsis thermalis* sp. 7203, and *Pleurocapsa* sp. PCC 7327. We also included sequences from *Synechocystis* sp. PCC 6803 and *T. elongatus* that do not contain the FaRLiP gene cluster (i.e., they have VL subunit isoforms only). To provide insight into the ancestral protein sequences, we additionally generated ancestral sequences of the FRL-specific subunits (see Materials and Methods and included them in the multiple sequence alignments.

Among all alignments, 130 residues were conserved in the FRL sequences but not the VL sequences, and therefore these residues were considered FRL-specific (highlighted yellow in **Supplementary Figure 6**). PsaI2, PsaJ2, and PsaL2 contained the largest fraction of FRL-specific residues, ~16%, and PsaA2 contained the largest number of FRL-specific residues overall (**Supplementary Table 1**). PsaB2 and PsaF2 contained the smallest fraction of FRL-specific residues in their sequences, <2%. Additionally, we marked FRL-specific residues that were also conserved in the ancestral sequence (vertical lines in **Supplementary Figure 6**), which implies that they were present in the corresponding ancestral FRL-PSI protein subunit prior to diversification events. Of the 130 residues that were determined to be FRL-specific, 88 were conserved in the ancestral sequences (68%). Interestingly, all FRL-specific residues in PsaB2 and PsaJ2 are conserved in their respective ancestral sequences, and only about half of the FRL-specific residues in PsaA2 are conserved in the ancestral sequence. These observations are consistent with the suggestion that there have been different evolutionary trajectories for PsaA2 and PsaB2.

**Figure 5:**
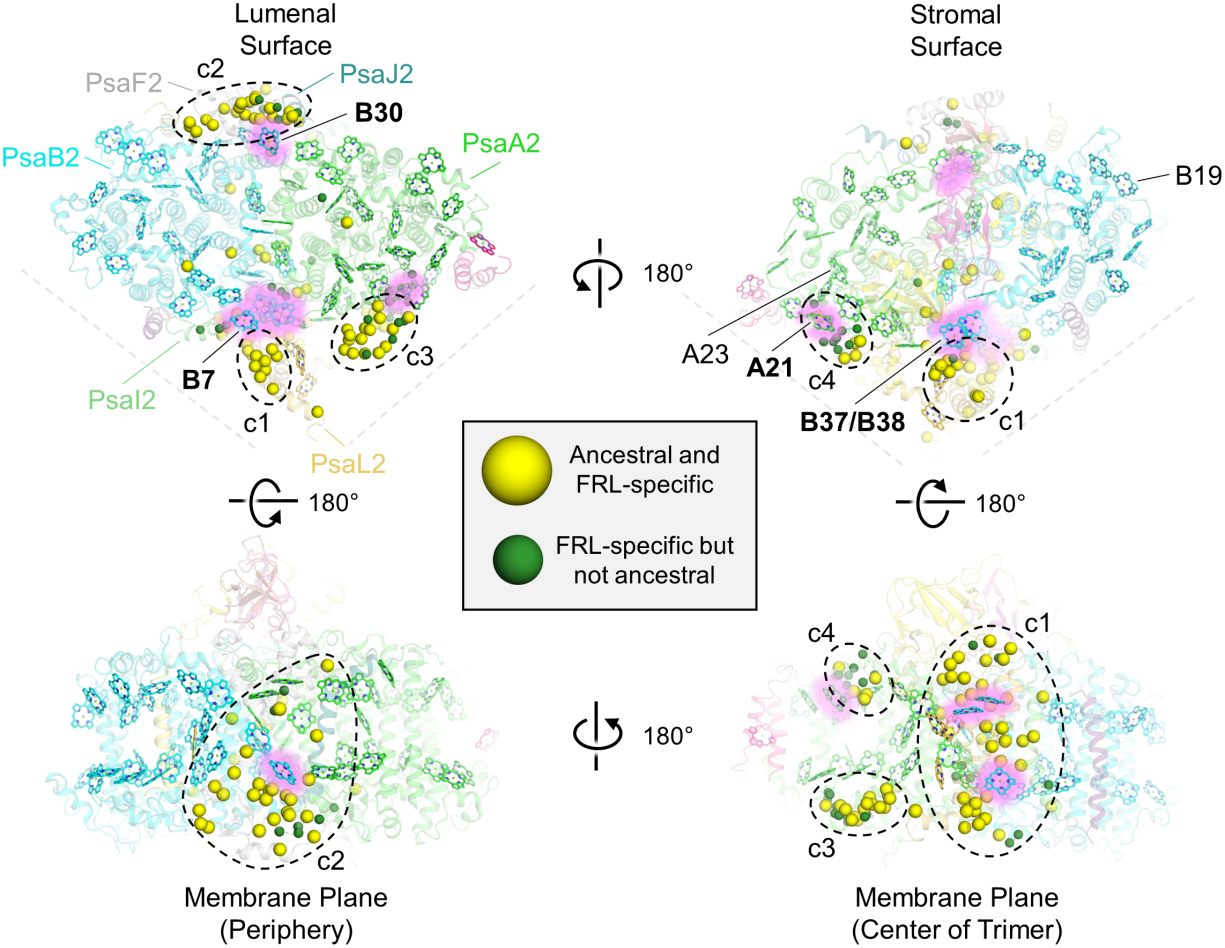
Location of FRL-specific residues in FRL-PSI. The structure of FRL-PSI from *F. thermalis* PCC 7521 is shown. This figure corresponds to the multiple sequence alignment shown in **Supplementary Figure 6**. Yellow spheres represent residues that are FRL-specific and are conserved in the ancestral sequence. Green spheres represent FRL-specific residues that are not conserved in the ancestral sequence. Chl *f*-binding sites that are conserved among the current FRL-PSI structures are shown with a pink glow and are labeled with bold font on each of the surface views (e.g., site B30). Chl *f*-binding sites that are observed to be species-specific based on the current structures are labeled with regular font (e.g., site B19). In the top left panel, the FRL-specific subunits are additionally labeled. The four clusters of FRL-specific residues are labeled c1 through c4.

To determine the physical location of the FRL-specific residues, especially those that would have been present in the ancestral subunits, we mapped them to the FRL-PSI structure from *F. thermalis* PCC 7521 (**Figure 5**). Four clusters (c1 to c4) of FRL-specific residues were observed: near PsaL2 found near the center of the trimer (labeled c1 in **Figure 5**), at the periphery of the complex near PsaF2 and PsaJ2 (labeled c2 in **Figure 5**), near the lumenal side of the dimerization interface in PsaA2 (labeled c3 in **Figure 5**), and near the stromal side of the dimerization interface in PsaA2 (labeled c4 in **Figure 5**). A few other FRL-specific residues did not fall into these clusters and were much more sparsely distributed.

#### 3.2.2 FRL-specific residues near the ETC and rotation of A_0B_

**Figure 6:**
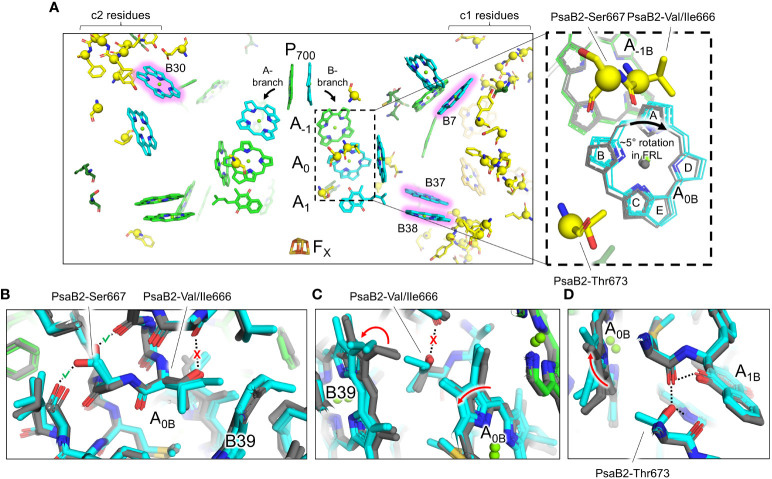
FRL-specific residues near the ETC cofactors and rotation of the Chl molecule in site A_0B_. **(A)** Structural view of FRL-PSI from *F. thermalis* PCC 7521 (PDB 7LX0) centered on the ETC cofactors. Chl molecules are shown as tetrapyrrole rings only. Chl *f* sites are shown with a pink glow. Phylloquinones are shown as the headgroup with a truncated tail. FRL-specific residues are shown as sticks with a sphere for their C_α_ atom, the latter of which is consistent with spheres shown in **Figure 3**. The dashed box region is magnified in the right panel. Here, the residues shown are from the structure of FRL-PSI from *F. thermalis* PCC 7521, but additional cofactors are shown from the following structures: FRL-PSI from *Synechococcus* sp. PCC 7335 (PDB 7S3D, colored), FRL-PSI from *H. hongdechloris* (PDB 6KMX, colored), VL-PSI from *H. hongdechloris* (PDB 6KMW, grey), non-FaRLiP PSI from *T. elongatus* (PDB 1JB0, grey), and non-FaRLiP PSI from *Synechocystis* sp. PCC 6803 (PDB 5OY0, grey). For the A_0B_ cofactor, the tetrapyrrole rings are labeled A-E. **(B–D)** Views of the protein near the A_0B_ cofactor and FRL-specific alterations that cause its rotation. The structures of FRL-PSI (colored) from *Synechococcus* sp. PCC 7335 and *H. hongdechloris*, and VL-PSI (grey) from *T. vulcanus* and *H. hongdechloris* are shown. Dashed lines denote H-bonding interactions. Where dashed lines are covered by a red “X”, the H-bond is unique to VL-PSI structures. Where dashed lines are covered by a green check mark, the H-bond is unique to FRL-PSI structures. Red arrows indicate where rotations occur in FRL-PSI structures relative to VL-PSI structures.

A few of the FRL-specific residues are found near the ETC cofactors (**Figure 5**) and are not contained within the clusters. This is surprising, because none of the four publications presenting FRL-PSI structures have identified Chl *f* molecules bound in the ETC, nor have they reported any other FRL-specific change to the ETC (Gisriel et al., 2020; Kato et al., 2020; Gisriel et al., 2021; Gisriel et al., 2022b; Gisriel et al., 2023b). The FRL-specific residues are primarily clustered around the B-branch of the ETC (**Figure 6A**). Although it had previously gone unnoticed, we now report that the Chl *a* molecule in site A_0B_ is altered in its position in all available FRL-PSI structures due to the FRL-specific residues near the B-branch. Namely, A_0B_ is rotated ~5°, pivoting about ring E of the tetrapyrrole causing rings A and B to exhibit the largest difference in position (magnified panel of **Figure 6A**). To confirm this subtle rotation of the Chl *a* molecule in site A_0B_, we performed various angular measurements showing that the rotation is indeed greater than would be expected for an average variation in structural coordinates (**Supplementary Figure 7**, **Supplementary Table 2**). The rotation is most obviously observed upon performing structural superpositions of all the FRL-PSI structures with several VL-PSI structures (magnified panel of **Figure 6A**).

The rotation of the Chl *a* in site A_0B_ of FRL-PSI is caused primarily by three FRL-specific residues from PsaB2: Val/Ile666, Ser667, and Thr673 (**Figures 6B–D**). In VL, position 666 of PsaB is a strictly conserved Thr whose hydroxyl moiety forms an interhelix H-bond (**Figure 6B**). Additionally, in VL, the C3 vinyl moiety of the Chl *a* in site B39 is directed toward position 666 (**Figure 6C**). In FRL, the Thr is replaced by Val or Ile, removing the interhelix H-bond (**Figure 6B**). This coincides with the rotation of the B39 C3 vinyl moiety to point away from position 666 (**Figure 6C**). This alteration allows the ring A region of A_0B_ to shift closer to B39 in FRL (**Figure 6C**). The stability lost by removal of the H-bond is compensated for by PsaB2-Ser667 in FRL which replaces a Gly in VL and creates a new interhelix H-bond (**Figure 6B**), stabilizing the FRL-specific structure. Additionally, position 673 of PsaB2, which is near ring B of A_0B_, is a Thr in FRL that replaces a conserved Ser in VL (**Figure 6D**). In both FRL- and VL-PSI structures, the hydroxyl moiety of the Ser or Thr sidechain donates an H-bond to the phylloquinone molecule in site A_1B_. Only in FRL, the additional methyl moiety of the Thr sidechain forces the B-ring of A_0B_ to rotate toward the A-ring, which is the same direction that was caused by the changes at positions 666 and 667 (**Figure 6D**). Thus, somewhat subtle changes to the protein alter the position of the A_0B_ cofactor in the ETC. We note that there are other FRL-specific residues near the ETC (**Figure 6A**), although their influence is not apparent.

#### 3.2.3 Cluster 1

Cluster 1 contains the largest fraction of FRL-specific residues, ~38, and is nearby three sites that have been proposed to contain Chl *f* molecules: B7, which is found toward the lumenal side of the complex, and the B37/B38 dimer that is found toward the stromal side of the complex (**Figures 5**, **6A**). One of the FRL-specific residues in cluster 1 is PsaI2-Tyr27. This residue coordinates a water molecule found only in FRL-PSI structures that donates an H-bond to the C2 formyl moiety of the Chl *f* molecule in site B7 (**Supplementary Figure 8A**) (Kato et al., 2020). PsaL2-Tyr93 also occurs specifically in FRL-PSI and its sidechain donates an H-bond to the backbone carbonyl oxygen atom of PsaI2-Tyr27, probably stabilizing the interaction of the FRL-specific water molecule and its H-bond with the B7 formyl moiety. PsaI2-Tyr27, PsaL2-Tyr93, and seven other nearby FRL-specific residues in PsaI2 and PsaL2 (**Figure 6A**) are all conserved in the corresponding ancestral sequence. This is strong evidence that site B7 was occupied by Chl *f* in the ancestral FRL-PSI complex.

**Figure 7:**
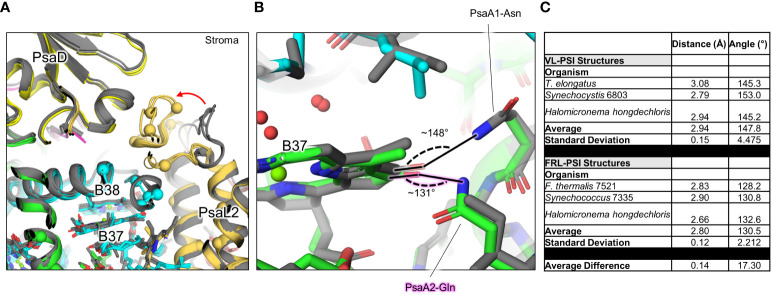
FRL-specific residues in cluster 1 (c1) near B37 and B38, and altered H-bonding interaction of B37. **(A)** The superimposed structures of FRL-PSI (colored) from *Synechococcus* sp. PCC 7335 and *H*. *hongdechloris*, and VL-PSI (grey) from *T. vulcanus* and *H*. *hongdechloris* are shown in the c1 vicinity (**Figures 5**, **6A**). FRL-specific residues are shown as spheres. **(B)** Altered H-bonding interaction of the B37 13^1^ keto oxygen. The FRL-specific PsaA2 Gln sidechain is labeled with a pink glow. **(C)** Angles of H-bonding interactions corresponding to panel **(B)** The table reveals that although the distance of the H-bond is consistent between the two structures, the angle is closer to optimal (~120°) in FRL-PSI structures.

The B37/B38 dimer that has also been suggested to contain Chl *f* is thought to give rise to a signature, low-energy contribution to the FRL-PSI absorbance spectrum (Tros et al., 2020). B37 was previously confirmed to contain Chl *f* at high occupancy by the cone scan method (Gisriel et al., 2021). The formyl moiety of the Chl *f* molecule in site B37 accepts an H-bond from the backbone amide nitrogen atom of the FRL-specific PsaB2-Gly697 as described previously (Gisriel et al., 2020; Gisriel et al., 2023b) (**Supplementary Figure 8B**). Additionally, this residue is one of four FRL-specific residues that surround the C2 position of B37. All four of these FRL-specific residues, and five more nearby from PsaL2 that contains an altered looping region, are conserved in their respective ancestral sequences (**Figure 7A**). These observations provide strong support for the hypothesis that B37 was also occupied by Chl *f* in ancestral FRL-PSI.

On the other side of the B37 tetrapyrrole, the 13^1^-keto oxygen atom accepts an H-bond which is observed in all PSI structures. Interestingly, there are two FRL-specific residues that alter this H-bond in FRL-PSI structures: PsaB2-Ala452 and Gln456 (**Figure 7B**). In VL-PSI structures, position 452 is a conserved Asn that donates the H-bond to the 13^1^-keto oxygen atom of B37, and position 456 is a conserved Ile. In FRL-PSI structures, the H-bond donor is flipped, such that position 452 is Ala that cannot donate an H-bond, and position 456 now donates the H-bond from the Gln sidechain (**Figure 7B**). The difference in H-bonding appears not to be equivalent, however, because the H-bonding angle is closer to ideal in all three FRL-PSI structures compared to structures of VL-PSI (**Figure 7C**). It is possible that the altered H-bond tunes the energy of Chl B37, perhaps causing a slight red shift. The FRL-specific PsaB2-Ala452 and Gln456 are both also conserved in the ancestral sequences, so it is likely that the altered H-bonding interaction of B37 was additionally present in the FRL-PSI ancestor.

Site B38 was not confirmed to contain Chl *f* by quantitative cone scan analyses of the cryo-EM maps (Gisriel et al., 2021). This led to the proposal that it contains a mixture of Chl *f* and Chl *a* (i.e., it contains Chl *f* at low occupancy), and/or that the low energy contribution observed spectroscopically could be explained by a heterodimer of Chl *f* and Chl *a* (Tros et al., 2020). Furthermore, there is not a strongly polar H-bond donor near the C2 position of the B38 tetrapyrrole ring. However, all three FRL-PSI structures replace a Trp in the VL sequence nearby the C2 position with a Phe (PsaB2-Phe22 in the sequence from *F. thermalis* PCC 7521) in the FRL sequence, leading to the suggestion that the extra volume allows for Chl *f*-binding (Gisriel et al., 2020; Tros et al., 2020; Gisriel et al., 2022b; Gisriel et al., 2023b), even if at partial occupancy. Interesting, there are no FRL-specific residues observed in the multiple sequence alignment that are conserved in all the extant sequences (**Supplementary Figure 6**). The Phe residue found in FRL sequences that replaces Trp found in VL sequences does so in only about half of the available FRL sequences, though it is strictly conserved in all VL sequences as Trp. These observations suggest that if site B38 contains Chl *f*, even at low occupancy, this is the case for only about half of the FRL-PSI complexes. It should also be noted that the ancestral sequence predicts Trp to be found in this position (**Supplementary Figure 6**); therefore, this site would not have been occupied by Chl *f* in the FRL-PSI ancestor. Rather, this would have evolved after diversification of FRL-PSI.

The FRL-specific residues described so far in this section account for only 20 of the ~38 residues in cluster 1, and these 20 all appear to be involved in the coordination of Chl *f* molecules. The specific functions of the other FRL-specific residues are unclear. Some may play a role in spectral tuning of Chl *a* molecules for more efficient energy transfer. It is also possible that some are important for FRL-specific insertion of Chl molecules into the FRL-PSI complex during assembly. For example, there is a stromal side loop of PsaL2 between its second and third transmembrane helices containing six FRL-specific residues (**Figure 7A**). This region is >12 Å away from the B37/B38 dimer, but nearly all the FRL-specific residues near B37/B38 are found between it and that stromal surface of PsaL2, so there may be FRL-specific dynamics present during FRL-PSI assembly that direct B37 and/or B38 into the binding site observed in the FRL-PSI structures. It is also possible that some FRL-specific residues influence the binding interfaces of the subunits close to the center of the trimer. Continuing with the same example, those six FRL-specific PsaL2 residues are found at the interface of PsaL2 with PsaD, so this interface may exhibit different molecular interactions in FRL compared to VL.

#### 3.2.4 Cluster 2

**Figure 8:**
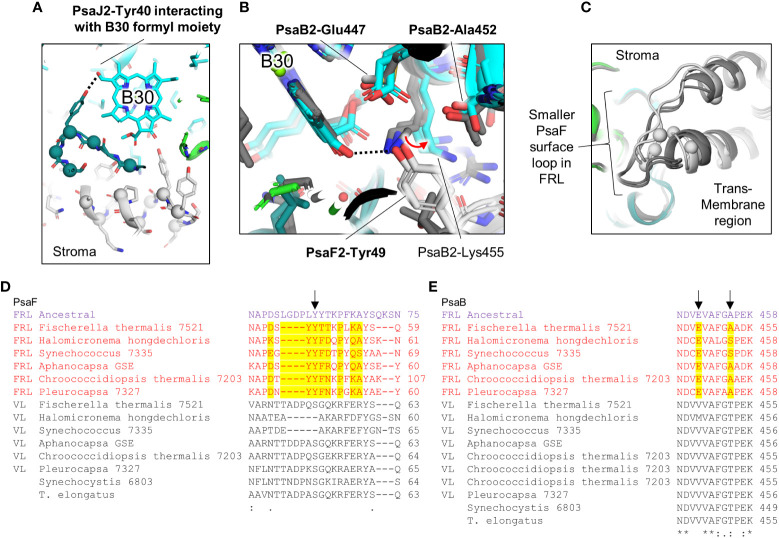
FRL-specific residues near Chl site B30 and corresponding partial sequence alignments. **(A)** Structure of FRL-PSI from *F. thermalis* PCC 7521 shown in the vicinity of B30. FRL-specific residues are shown as spheres. **(B)** Vicinity of B30 showing the H-bond alteration that occurs in FRL. In panels **(B, C)**, VL-PSI structures are shown in grey. **(C)** Stromal surface loop of PsaF near B30 that is shorter in FRL-PSI (white) than it is in VL-PSI (grey). **(D)** Partial sequence alignment of PsaF corresponding to the shortened FRL-specific loop and Tyr residue that H-bonds to the 13^1^-keto oxygen of the Chl *f* in site B30. **(E)** Partial sequence alignment of PsaB highlighting its FRL-specific residues near the Chl *f* in site B30. In panels **(D, E)**, the black arrows correspond to the residues labeled in bold font in panel **(B)**.

Cluster 2 also spans the entire membrane (**Figure 5**). It is found on the opposite side of a FRL-PSI monomer relative to cluster 1 and comprises ~30 FRL-specific residues, most of which are closer to the lumenal side of the complex. This cluster is also nearby a Chl site that exhibits high confidence for binding Chl *f*, site B30, which is also found on the lumenal side. One of the FRL-specific residues in cluster 2 is PsaJ2-Tyr40 whose sidechain provides an H-bond to the C2 formyl moiety of the Chl *f* in site B30 as described previously (**Figure 8A**) (Gisriel et al., 2020; Gisriel et al., 2021). PsaJ2-Tyr40 is one of five sequential residues, PsaJ2-39 through 43, that is a FRL-specific insertion in PsaJ2 compared to PsaJ1 (VL) sequences; therefore, all residues in this insertion probably help to stabilize the FRL-specific H-bonding interaction of the Tyr sidechain (**Figure 8A**). There are also three FRL-specific hydrophobic residues just outside of this region that probably help to stabilize the configuration as well. All these FRL-specific residues are found in the ancestral sequence, and therefore the occurrence of Chl *f* in site B30 is a likely characteristic of the ancestral FRL-PSI complex.

Similar to the Chl *f* in site B37 in cluster 1, the 13^1^-keto oxygen atom of B30 also has a FRL-specific environment different from VL-PSI due to residues in cluster 2. In VL-PSI structures, the 13^1^-keto oxygen atom of the Chl *a* in site B37 accepts an H-bond from a Lys sidechain in PsaB. Although the same Lys is conserved in FRL-PSI, its sidechain is found in a different position, directed away from B30, removing its H-bonding interaction (**Figure 8B**). This is caused by the FRL-specific PsaF2-Tyr49 of a shortened surface loop (**Figure 8C**) that appears to displace the Lys sidechain (PsaB2-Lys544 in the sequence from *F. thermalis*) and it forms a new H-bond to the 13^1^-keto oxygen atom from its sidechain (**Figure 8B**). PsaF2-Tyr49 also donates an H-bond to the FRL-specific PsaB2-Glu447, which likely stabilizes the H-bonding interaction of PsaF2-Tyr49 with B30. Nearby is also the FRL-specific PsaJ2-Leu31 that replaces a conserved Asn found in VL sequences (**Supplementary Figure 9**), thus altering the polarity of the environment near the PsaF2-Tyr49 H-bond with the 13^1^-keto oxygen atom of the Chl *f* in site B30. These three FRL-specific residues (PsaB2-Glu447, PsaF2-Tyr49, and PsaJ2-Leu31), and at least three others nearby that stabilize them, probably participate in tuning the energy of the Chl *f* in site B30. Additionally, all these FRL-specific residues are conserved in the FRL ancestral sequences (**Figures 8D, E**), and therefore the FRL-specific chemical environment of the Chl *f* in site B30 described here was most likely present in the ancestral FRL-PSI complex.

Like cluster 1 on the opposite side of the FRL-PSI complex, only about half of the residues in cluster 2 appear to stabilize the interactions with Chl *f* observed in the FRL-PSI structures. It seems likely that subunit interactions may be altered due to FRL-specific residues as well. For example, there are three consecutive FRL-specific residues in PsaF2, residues 25-27 (**Supplementary Figure 6**), that closely interact with PsaB2, yet they are far from any proposed Chl *f*-containing site, the shortest distance being site B30 that is ~25 Å away. These FRL-specific residues may alter the subunit-subunit interactions and thus stability of the complex. Additionally, it is observed that three Chl *a* molecules are bound by PsaF and PsaJ In VL-PSI, but none of these Chl-binding sites are present in FRL-PSI. Thus, it seems possible that some of the FRL-specific residues deter Chl *a* binding. The reason for this is unknown but it may involve energy transfer (or the blockage of it) from peripheral IsiA-like antenna as suggested previously (Gisriel et al., 2020).

#### 3.2.5 Clusters 3 and 4

**Figure 9:**
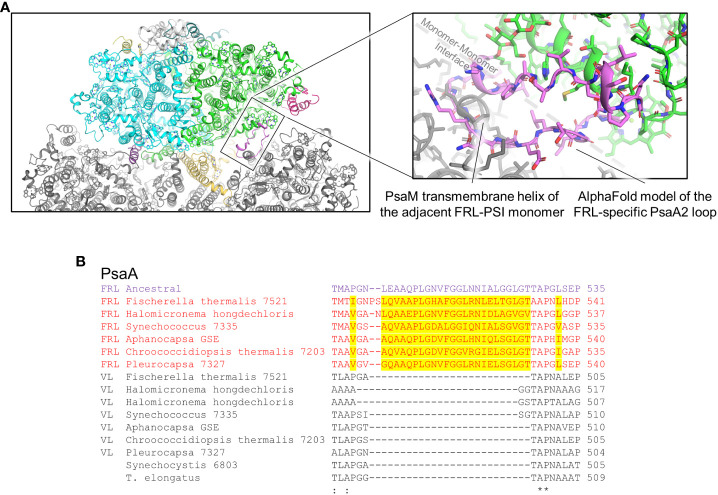
FRL-specific looping region comprising cluster 3 that is unresolved in cryo-EM structures. **(A)** The region of the PsaA2 loop found at the interface of PSI monomers. The magnified region shows the AlphaFold model in more detail (pink). **(B)** Partial sequence alignment showing the FRL-specific looping region. FRL-specific residues are highlighted.

Clusters 3 and 4 are both found on the PsaA2-side of the FRL-PSI complex at the monomer-monomer interface but are on opposite sides of the membrane: cluster 3 is on the lumenal side and cluster 4 is on the stromal side (**Figure 5**). All the residues in cluster 3 are found in a looping region of PsaA2 between transmembrane helices 7 and 8 (**Figure 9A**). This FRL-specific loop is an ~25 residue insertion relative to VL-PsaA sequences (**Figure 9B**). This region is unmodeled in all the FRL-PSI structures implying that it is especially flexible. Consistent with this hypothesis, Jpred4 secondary structural prediction (**Supplementary Data 2**) (Drozdetskiy et al., 2015) and AlphaFold (Jumper et al., 2021) models both suggest little secondary structure for the loop except a small α-helical region, and the AlphaFold model clashes with PsaM from the adjacent PSI monomer, an unlikely configuration. This FRL-specific loop does not appear to interact directly with any Chl-binding site either from its monomer or the adjacent one, so its function is unknown.

Cluster 4 residues are also all found in a single FRL-specific loop of PsaA2, between its 4th and 5th transmembrane helices. This is nearby a site containing substantial alterations in the FRL-PSI structures where a trimer of Chl *a* molecules found in VL-PSI is altered to contain a dimer of Chl molecules in FRL-PSI, one of which is a Chl *f* in site A21 (Gisriel et al., 2020; Gisriel et al., 2023b) (**Supplementary Figure 10A**). Like the cluster 3 loop, the cluster 4 loop also exhibits variation among species. About 1/3rd of its residues are found in the ancestral sequence, but the others are not (**Supplementary Figure 10B**). Likewise, the full sequence alignment shows that ~15% of PsaA2 sequences lack this FRL-specific loop, including the basal sequences in the PsaA2 subtree. Thus, it seems likely that some form of loop alteration was present in the FRL-PSI ancestor, probably causing the Chl trimer conversion to the FRL-specific dimer, but it is unclear whether that alteration bound Chl *f* in site A21. In the present FRL-PSI structures, the C2 formyl moiety of A21 accepts an H-bond from the backbone amide nitrogen of a Leu with the cluster 4 loop (**Supplementary Figure 10A**), so the sidechain identities need not necessarily be tightly regulated. These observations suggest that site A21 may not be vital for achieving charge separation using FRL, and that some extant FRL-PSI complexes do not exhibit the FRL-specific loop that provides its H-bonding interactions observed in the present structures. However, it is interesting that clusters 3 and 4 are likely to influence the same region of the FRL-PSI complex, but on different sides of the membrane. It may be that these FRL-specific changes have more to do with altering the monomer-monomer interfaces than they do with absorbing FRL.

## 4 Discussion

### 4.1 A timeline for the evolution of FaRLiP photosystems

The first goal of this study was to provide a more detailed picture of how PSI in the FaRLiP response evolved. Unlike some of the FRL-PSII subunits, none of the ancestral gene duplications leading to FRL-specific PSI-subunit paralogs appear to have occurred before the MRCA of cyanobacteria or during the early evolution of cyanobacteria. Instead, these duplications seem to have occurred already when the major clades of cyanobacteria had started to diversify. More specifically, the FRL-PSI sequences appear more closely related to VL sequences from macrocyanobacteria. This result is consistent across all phylogenetic trees produced here, despite those of the smaller subunits (PsaL, PsaF, PsaI, and PsaI) being less well resolved due to lack of phylogenetic signal. This prompted us to reexamine the topology of the FRL-specific PsbB (CP47) and PsbH subunits of PSII, which also showed a late divergence in our previous work (Gisriel et al., 2022a). Similarly, FRL-CP47 and FRL-PsbH sequences also clustered within VL sequences belonging to macrocyanobacteria (Oscillatoriales, Chroococcales). The evolutionary relationship between microcyanobacteria, branching as a sister group to macrocyanobacteria, is well resolved in phylogenetic and phylogenomic studies (Shih et al., 2013; Sánchez-Baracaldo, 2015; Chen et al., 2021; Strunecký et al., 2023); therefore, this topology in combination with our phylogenetic studies suggests that Nodosilineales, the only known group of microcyanobacteria with FaRLiP, inherited the gene cluster via HGT. It was previously suggested that the patchy distribution of FaRLiP across cyanobacteria could be due to HGT (Gan et al., 2014a; Gan and Bryant, 2015), with the probable exception of Nostocales (Gan et al., 2014a) and closer relatives, while (Antonaru et al., 2020) noted congruence of the unrooted phylogeny of the *apcE2* gene with that of the species tree of cyanobacteria, even at genus level, demonstrating a good degree of vertical inheritance and frequent losses, most confidently in the Nostocales. Given that these are single tree phylogenies, and that some of the smaller sequence alignments do not have high resolving power, it is still possible that these results can be explained by other phenomena such as incomplete lineage sorting of duplicated genes as a consequence of the rapid diversification process occurring around the initial divergence of micro- and macrocyanobacteria. This has been demonstrated recently for incongruences in gene and species trees within the heterocystous cyanobacteria (Pardo-De la Hoz et al., 2023) and is likely to affect other cyanobacterial relationships as well. Future analyses should also consider the conservation of gene synteny in the cluster across taxa and disparities in G+C content in the FaRLiP cluster relative to the rest of the genome to infer and single out HGT events more confidently.

In combination with our previous study on the evolution of FRL-PSII, we can describe two stages in the evolution of the FaRLiP: an early and a late stage. In the early stage, the capacity to produce Chl *f* as well as the capacity to drive charge separation and water splitting in PSII using FRL originated. It should be noted that this scenario does not account for the origin of Chl *d* synthesis. However, considering that only one Chl *d* is required per PSII complex and that it can be produced spontaneously in the presence of thiols and oxygen (Ho and Bryant, 2019; Bryant et al., 2020), there may not be any specific requirement for an enzyme to have evolved to perform its synthesis early on or in FaRLiP cells. In addition, it is consistent with recent work demonstrating that effective acclimation to FRL can occur in the absence of FRL-PSI in a case where a naturally occurring *Chroococcidiopsis* strain lost several of the FRL-specific genes (Billi et al., 2022).

**Figure 10:**
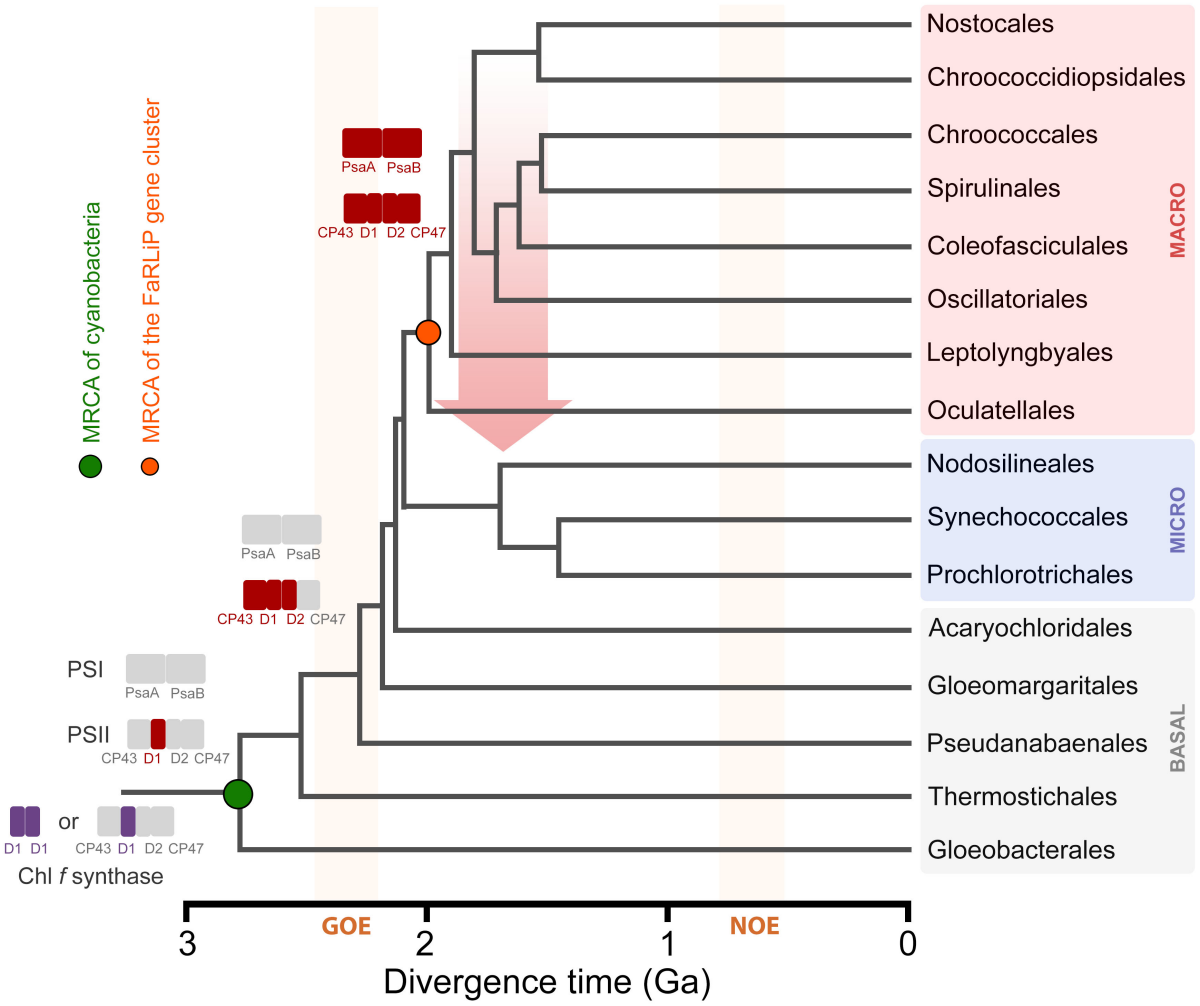
A schematic representation of the evolution of FaRLiP along a chronogram of cyanobacterial evolution. Divergence times are approximately taken from (Boden et al., 2021). Branch lengths in this instance denote time in billions of years before the present (Ga). Colored rectangles represent photosystem subunits for PSI and PSII. The Chl *f* synthase is represented as a homodimer (double purple rectangle) (Shen et al., 2019) or a standard PSII harbouring the divergent form of D1 (Trinugroho et al., 2020). GOE stands for the Great Oxidation Event, and NOE stands for Neoproterozoic Oxygenation Event. The red arrow represents a horizontal gene transfer event of a full gene cluster from an early evolving macrocyanobacterium to a potential ancestor of the Nodosilineales.

In the late stage, the utilization of FRL by PSII was optimized and FRL-PSI was introduced. A complete gene cluster could have become assembled at around the time macrocyanobacteria began to diversify. Using some of the latest work attempting to time the diversification of cyanobacteria (Boden et al., 2021; Fournier et al., 2021), it is possible to assign some reasonable timings for these events (**Figure 10**). For example, assuming that ChlF and FRL-PsbA acquired their functions relatively soon after they duplicated from standard, but early forms of PsbA, prior to the MRCA of cyanobacteria, this would constrain their origin to before the Great Oxidation Event (GOE), in the range of 2.6 (Boden et al., 2021) to before 3.0 Ga (Fournier et al., 2021). The duplication of FRL-PsbD and FRL-PsbC would be placed just before or at around the start of the GOE, while all duplications enabling the assembly of a FaRLiP cluster had already occurred by about 2.2 to 2.0 Ga, the time that extant macrocyanobacteria lineages began to radiate (Boden et al., 2021; Fournier et al., 2021). Remarkably, the MRCA of extant Nodosilineales is timed in the work by Boden et al. (2021) to about 0.8 Ga. Boden et al. (2021) performed the most taxa-rich molecular clock analysis of cyanobacterial evolution available, which contains several representatives from diverse Nodosilineales, including some of the FaRLiP strains. If HGT indeed occurred, these divergence times permit ample time for an early-evolving cyanobacterium of the Nodosilineales to have acquired a full cluster from the early radiating macrocyanobacteria.

### 4.2 Multiplicity of PSI forms

Some cyanobacteria encode in their genomes up to four distinct PsaB paralogues, reviewed in Cardona (2015). PsaB3 was first noted in a proteomic study of the non-FaRLiP organism, *Nostoc punctiforme*, in which the heterocyst-specific, uptake hydrogenase had been knocked out. The study showed that both PsaB paralogs were produced by the cells, but PsaB3 exhibited lower abundance in the isolated heterocysts of the mutant compared with heterocysts of the wild-type strain (Ekman et al., 2011). This variant was named PsaB2 in their studies because the genome of *N. punctiforme* only encodes two paralogs of PsaB. Homology modeling and sequence comparisons showed that this ‘PsaB2’ had a number of unique amino acid changes, including a Gln insertion, next to the F_X_ cluster and a phylloquinone cofactor (Magnuson et al., 2011; Magnuson and Cardona, 2016). These changes are confirmed here for all PsaB3 as delimited in **Supplementary Figure 5**. Later, it was shown that PsaB3 from *Chlorogloeopsis fritschii* PCC 9212, also a heterocystous cyanobacterium, was produced under FRL and incorporated into PSI complexes in parallel with the PsaB2 of FaRLiP (Gan et al., 2014b; Ho and Bryant, 2019). In another study, it was shown that *Leptolyngbya ohadii*, whose genome encodes a VL PsaB and PsaB3 but lacks FaRLiP, constitutively expressed both subunits and incorporated them into PSI tetramers under standard laboratory conditions (Niedzwiedzki et al., 2023). Therefore, it appears as if the expression of PsaB3 is constitutive in those strains that have it. How the photochemical properties of a PsaB3-containing PSI are altered relative to the standard complex and the significance of producing multiple forms of PSI under the same conditions remain to be investigated and resolved.

### 4.3 Structural diversification of FRL-PSI

The second goal of this study was to reveal the diversity of structural characteristics in FRL-PSI, and which of those characteristics were probably exhibited by an ancestral FRL-PSI (**Table 2**). Based on the current data, the Chls in the ETC are all Chl *a*, which would have also been the case for the ancestral form of FRL-PSI. Additionally, they all exhibit a slightly rotated A_0B_ Chl *a*, which would have also been present in the ancestral form. The effects of this rotation on charge separation in FRL-PSI is difficult to predict. A more pronounced rotation of photochemical pigments is observed in PSII relative to the case in the purple bacterial reaction center, where one of the two ‘P’ Chls of the PSII core is shifted by ~20°. This rotation disrupts orbital overlaps between P_D1_ and P_D2_ in PSII relative to P_L_ and P_M_ in the purple bacterial reaction center, substantially decreasing the electronic coupling and preventing the P_D1_P_D2_ pair from generating a low energy trap (Cardona et al., 2012). It is plausible that the rotation we observe in A_0B_ could alter its electronic coupling with A_-1B_ (and potentially its excitonic coupling with the B38/B37 Chl *f* pair). While current sequence and structural data do not support the occurrence of a Chl *f*-binding site at A_-1A_ or A_-1B_ as proposed by Nürnberg et al. (2018) on the basis of spectroscopic measurements, the rotation of A_0B_ suggests that some fine tuning of the charge separation process in FRL-PSI has indeed evolved.

**Table 2:** Chl *f*-containing sites in the current cryo-EM structures and prediction of the ancestral Chl *f* sites based on residue conservation in the ancestral sequence reconstruction.

	A_0B_ rotation	B7 Chl *f*	B37 Chl *f*	B38 Chl *f*	B30 Chl *f*	A21 Chl *f*	A23 Chl *f*	B19 Chl *f*
*F. thermalis* 7521	Y	Y	Y	Y	Y	Y	N	N
*Synechococcus* 7335	Y	Y	Y	Y	Y	Y	N	Y
*H. hongdechloris*	Y	Y	Y	Y	Y	Y	Y	N
**Ancestral**	**Y**	**Y**	**Y**	**N**	**Y**	**N**	**N**	**N**

Bold text corresponds to the inferred ancestral state.

Sites B7, B37, and B30 are most likely highly occupied by Chl *f* molecules in all extant FRL-PSI complexes, which would have been the case also for the ancestral form of FRL-PSI. Notably, the Chl *f* molecules in sites B37 and B30 exhibit altered H-bonding interactions to their 13^1^-keto oxygen atoms that probably tune their spectral properties differently than VL-PSI. Another characteristic shared by extant FRL-PSI complexes, which would have also been found in the ancestral form of FRL-PSI, is the lumenal-side looping region denoted as cluster 3 above. The role of this loop is presently unclear, but it may influence the monomer-monomer interfaces of the FRL-PSI trimer.

The Chl *f* molecules bound in sites B38, A21, A23, and B19 would have evolved later in different lineages. While the current FRL-PSI structural data support the assignment of B38 and A21 being occupied by Chl *f* in *F. thermalis* PCC 7521, *H. hongdechloris*, and *Synechococcus* sp. PCC 7335, our sequence alignments show that they may not bind Chl *f* in certain species for which FRL-PSI structures are not currently available. In sites A23 and B19, there is much more variation among species, which is currently captured by the available structural data.

An important final observation about the structural analysis concerns the locations of FRL-specific residues relative to the Chl *f* molecules. As described above, the majority of FRL-specific residues are clustered nearby sites that are known to contain Chl *f* ubiquitously in extant FaRLiP species (i.e., sites B7, B37, and B30). We have described the residues that appear to directly influence Chl *f* binding and/or energy tuning, but the role of many FRL-specific residues is unclear. Based on the locations of the FRL-specific residues relative to the Chl *f* molecules it seems reasonable to suggest that they play a role in Chl *f* insertion. This is most obvious in **Figures 5**, **6A**, in which from the context of a single FRL-PSI monomer, the FRL-specific residues radiate toward the periphery. One could imagine that during PSI biogenesis, the core PsaA(2) and PsaB(2) subunits would initially assemble with the ETC cofactors, after which peripheral Chl molecules are then inserted. If this were to be the case, it would make sense that FRL-specific residues would guide the insertion of Chl *f* molecules into the sites that are especially important to bind Chl *f*. In other words, seemingly purposeless FRL-specific residues observed in the fully formed FRL-PSI structures might be vital for Chl *f* insertion dynamics during FRL-PSI biogenesis.

## 5 Conclusion

We have shown that FRL-PSI subunits evolved relatively late in the evolution of FRL photoacclimation relative to some of the FRL-PSII subunits, likely arising during the early radiation of the macrocyanobacteria. We provide some evidence suggesting that a degree of HGT could have occurred early on, although this is not unequivocal. Additionally, we have proposed which features of FRL-PSI are common among FaRLiP species, and which are present in only certain species. The former allowed us to hypothesize features of the ancestral form of FRL-PSI, which contained three Chl *f*-binding sites and a rotated Chl *a* molecule in the A_0B_ site of the ETC. The effect of this rotation is unclear, but it warrants further investigation. Collectively, these observations provide a better understanding of how FaRLiP arose from both structural and phylogenetic perspectives.

## Data availability statement

The original contributions presented in the study are included in the article/**Supplementary Material**. Further inquiries can be directed to the corresponding authors.

## Author contributions

CG: Conceptualization, Data curation, Funding acquisition, Investigation, Methodology, Resources, Visualization, Writing – original draft, Writing – review & editing. DAB: Funding acquisition, Writing – review & editing. GB: Funding acquisition, Writing – review & editing. TC: Conceptualization, Data curation, Formal Analysis, Funding acquisition, Investigation, Methodology, Resources, Visualization, Writing – review & editing.
