# Supplementary material for: Molecular diversity and evolution of far-red light-acclimated photosystem I
Source: Front Plant Sci. 2023 Nov 20;14:1289199. doi: 10.3389/fpls.2023.1289199 (PMC10694217; doi:10.3389/fpls.2023.1289199)
Supplement: Supplementary file 2 [file DataSheet_2.docx]

Supplementary Material

Molecular diversity and evolution of far-red light-acclimated photosystem I

Christopher J. Gisriel*, Donald A. Bryant, Gary W. Brudvig, Tanai Cardona*

*** Correspondence:**Christopher J. Gisriel and Tanai Cardona
[christopher.gisriel@yale.edu](mailto:christopher.gisriel@yale.edu), [t.cardona@qmul.ac.uk](mailto:t.cardona@qmul.ac.uk)

**1 Supplementary Data**

**Supplementary Data 1.** Raw phylogenetic data used herein (external).

**Supplementary Data 2.** Jpred4 output on PsaA2 for analysis of the cluster 2 loop (external).

**2 Supplementary Figures**


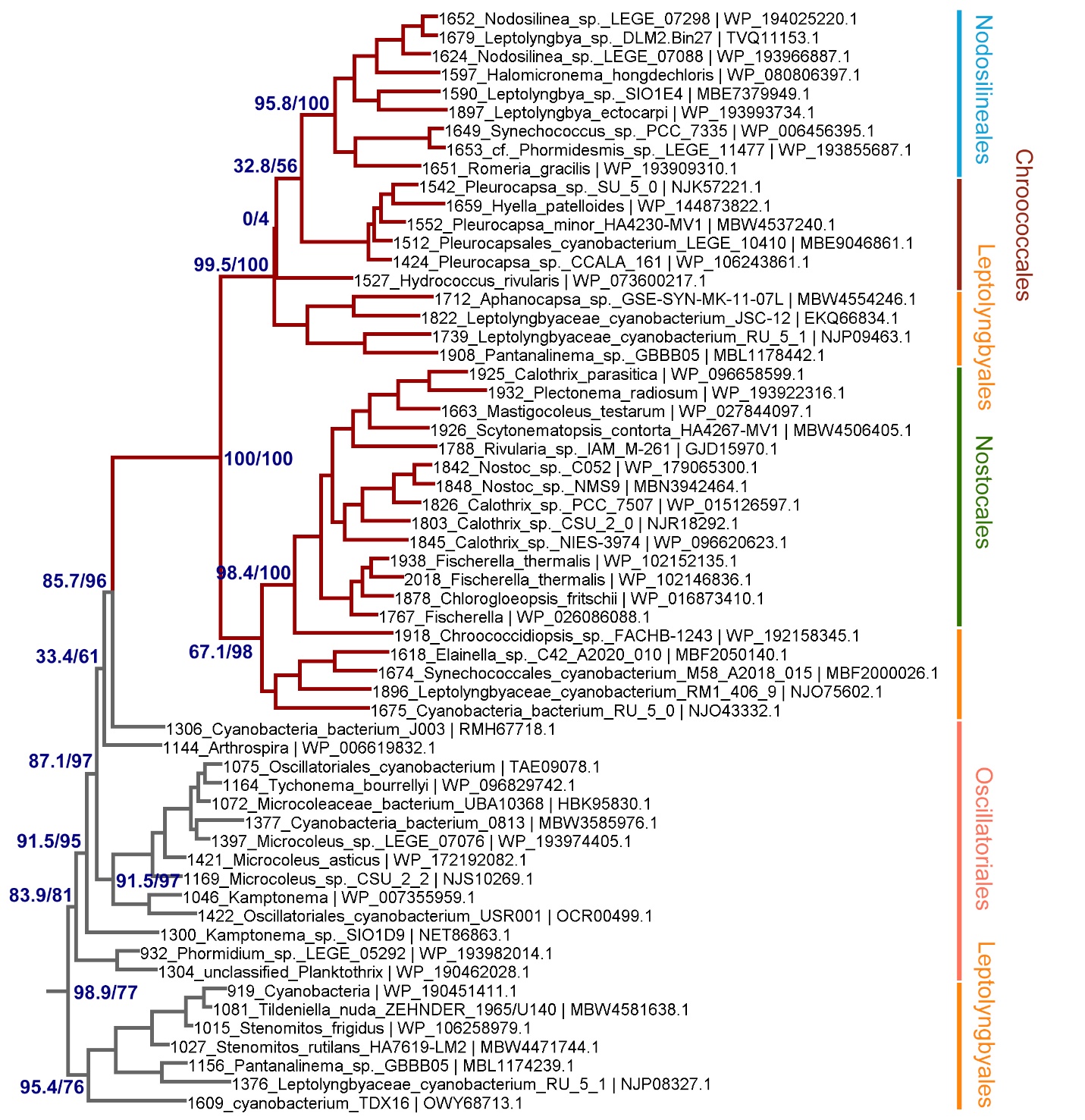


**Supplementary Figure 1.** Close-up on the Maximum Likelihood ML phylogeny of PsaB around the PsaB2 sequences (dark red branches). Grey branches represent the visible light VL forms. Orders are shown to the right. Support values are by the Ultrafast Bootstrap and the Average Likelihood Ratio Test methods, respectively, and these are only shown on selected branches for clarity. The complete tree is found in the Supplementary Data 1.


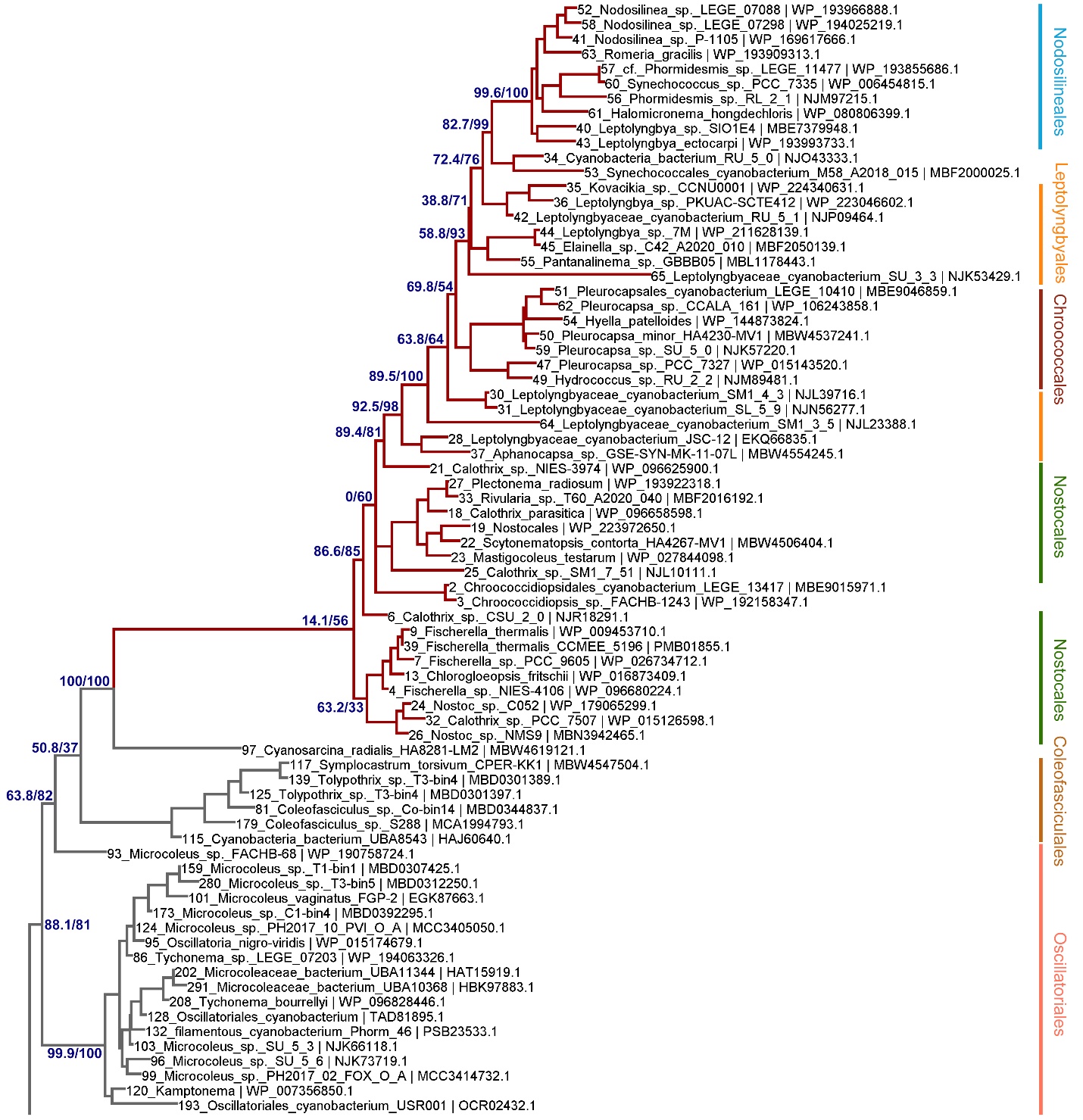


**Supplementary Figure 2.** Close-up on the ML phylogeny of PsaL around the PsaL2 sequences (dark red branches). Grey branches represent the VL forms. Orders are shown to the right. Support values are by the Ultrafast Bootstrap and the Average Likelihood Ratio Test methods, respectively, and these are only shown on selected branches for clarity. The complete tree is found in the Supplementary Data 1. Sequences 139 and 125 are misclassified DNA fragments from metagenomes, but likely from *Coleofasciculus*-like strains. The long branch corresponding to sequence 65 originates from a DNA contig of a metagenomic study. The contig has only four FaRLiP genes, including *psaI2* (encoding sequence 71 in **main text Figure 4**), and *psaJ2* and *psaF2*, which appeared identified as pseudogenes (likely non-functional). The unusually long PsaL2 gene also suggests that the gene may be eroding. In addition, the two genes up- and downstream of this four-gene cluster encoded for proteins with unrelated functions not known to be found within FaRLiP clusters. Therefore, it looks as if this contig comes from a cyanobacterium, which might be in the process of losing FaRLiP. The contig can be found here: accession JAAUUY010000320.1.


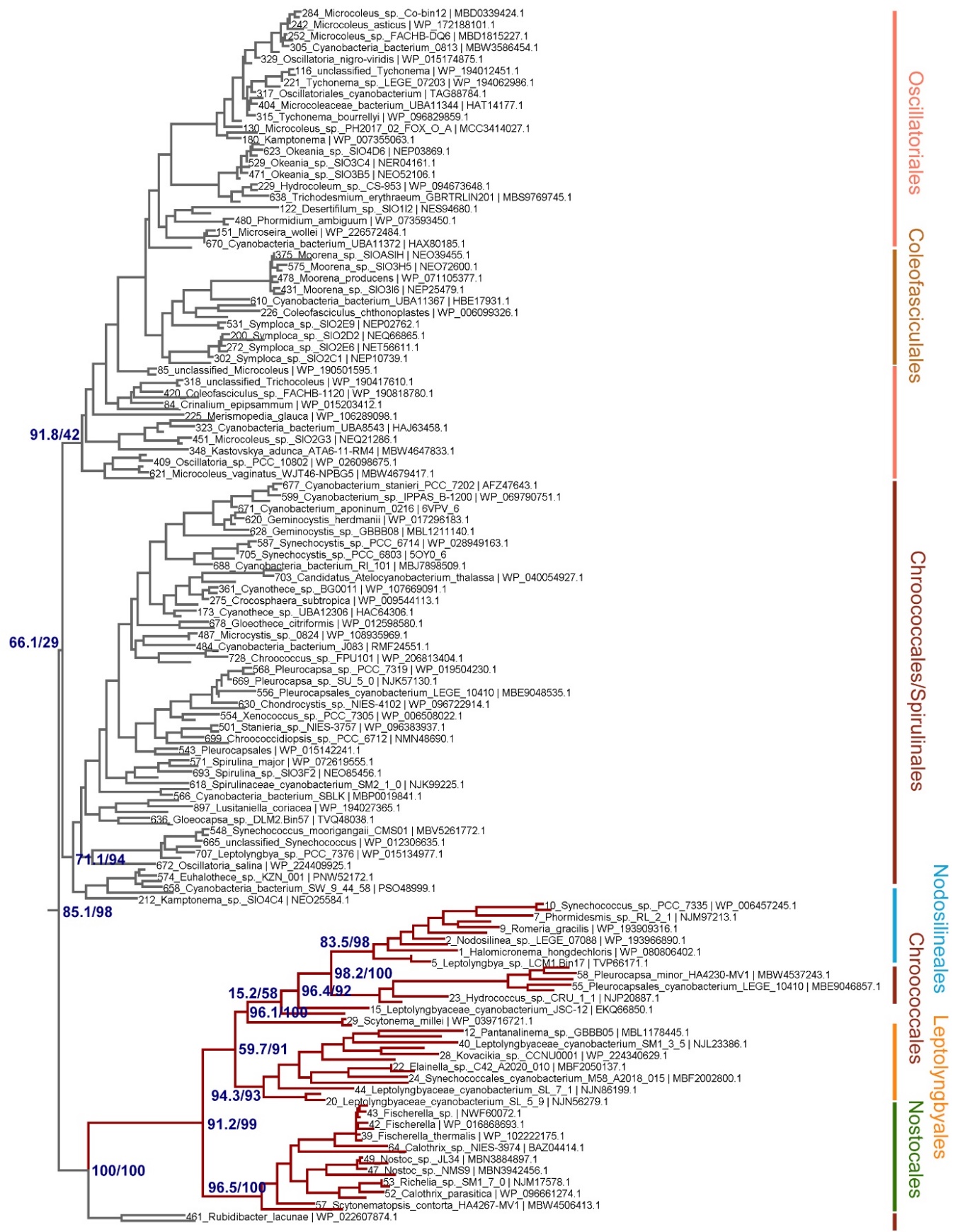


**Supplementary Figure 3.** Close-up on the ML phylogeny of PsaF around the PsaF2 sequences (dark red branches). Grey branches represent the VL forms. Orders are shown to the right. Support values are by the Ultrafast Bootstrap and the Average Likelihood Ratio Test methods, respectively, and these are only shown on selected branches for clarity. The complete tree is found in the Supplementary Data 1.


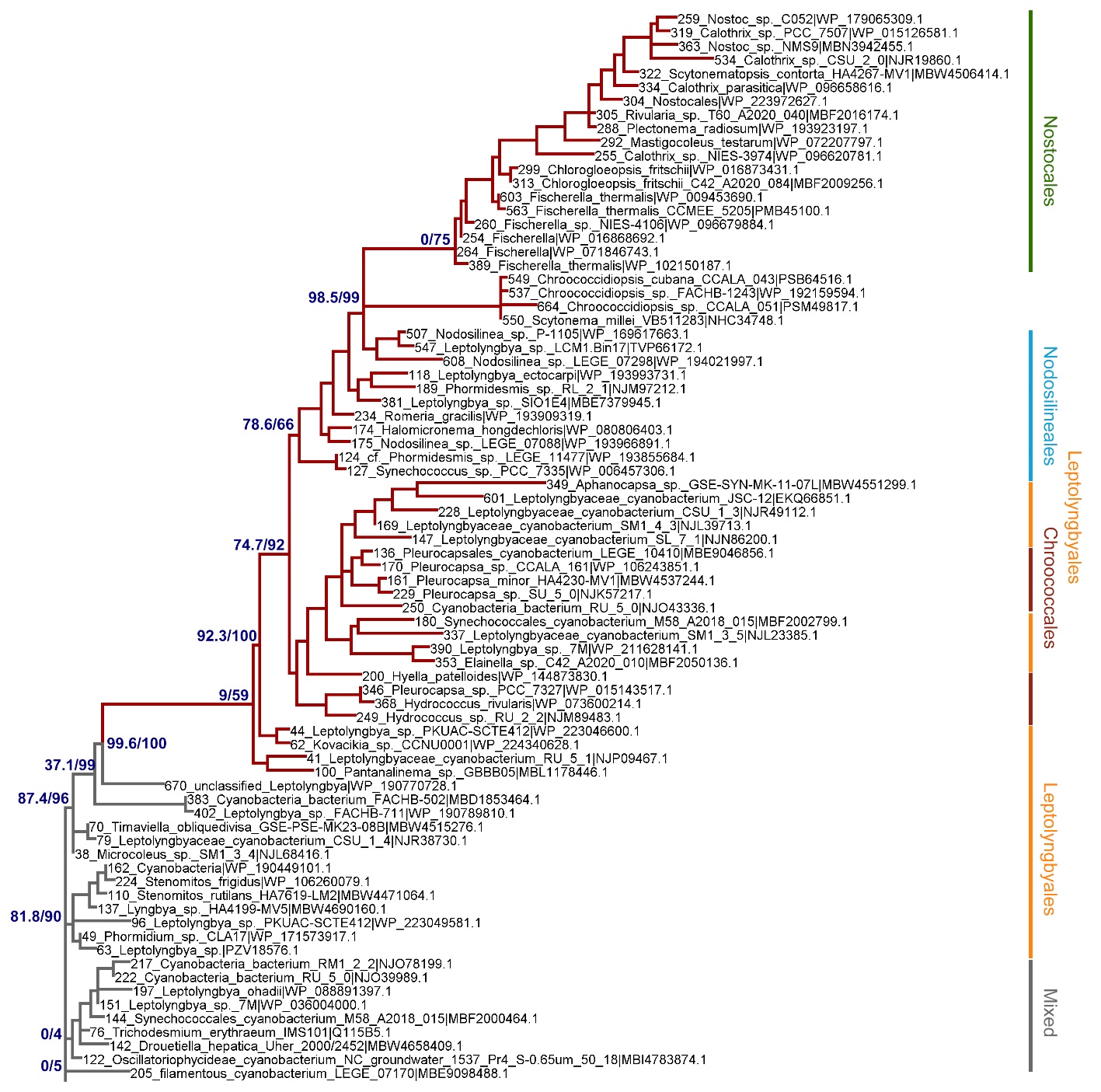


**Supplementary Figure 4.** Close-up on the ML phylogeny of PsaJ around the PsaJ2 sequences (dark red branches). Grey branches represent the VL forms. Orders are shown to the right. Support values are by the Ultrafast Bootstrap and the Average Likelihood Ratio Test methods, respectively, and these are only shown on selected branches for clarity. The complete tree is found in the Supplementary Data 1.


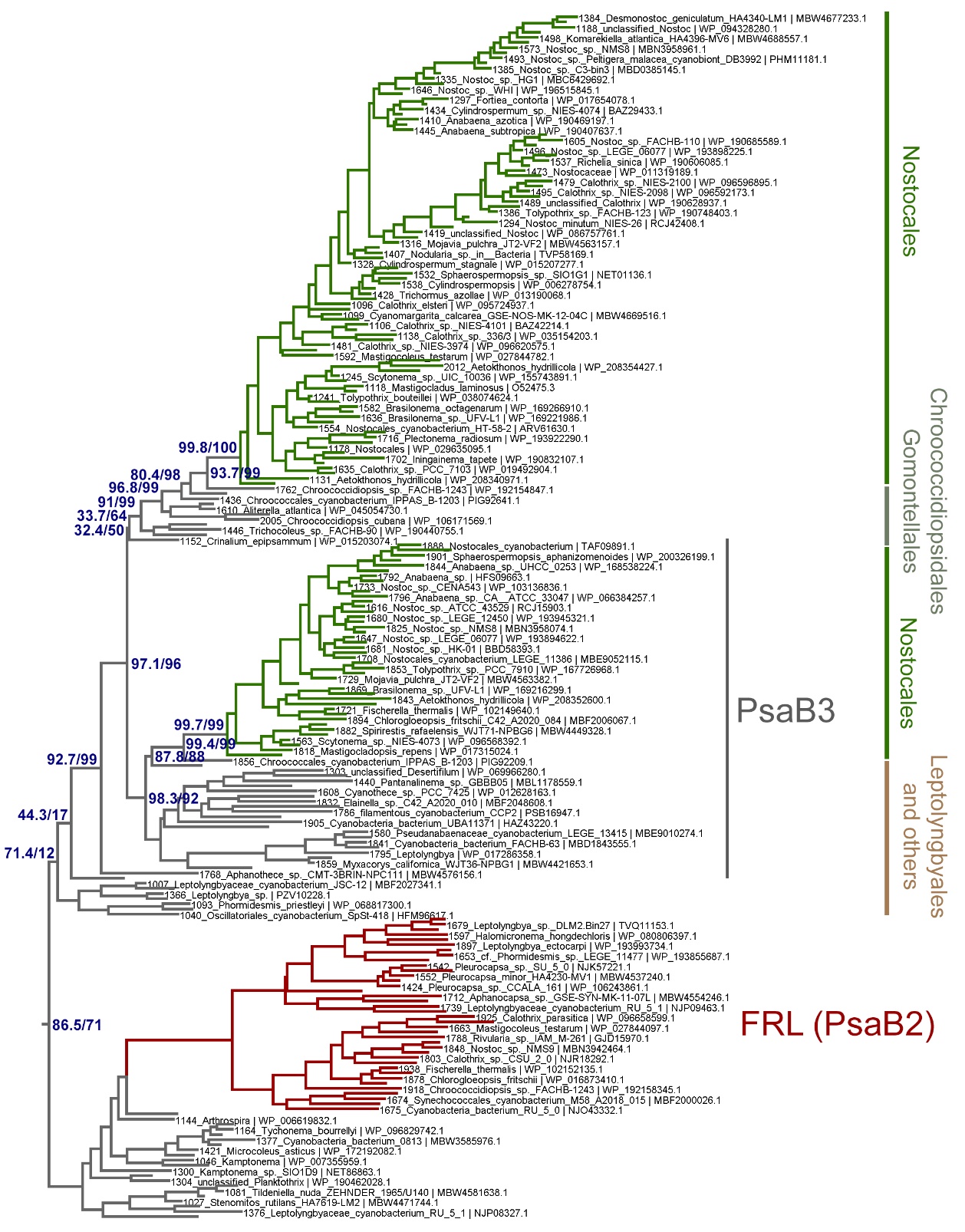


**Supplementary Figure 5**. Close-up on the ML phylogeny of PsaB around a third paralog referred as PsaB3. Grey branches represent the VL forms. The VL sequences found in Nostocales (green branches) are split into two groups, one of which correspond to a previously identified paralog. Orders are shown to the right. Support values are by the Ultrafast Bootstrap and the Average Likelihood Ratio Test methods, respectively, and these are only shown on selected branches for clarity. The complete tree is found in the Supplementary Data 1. Evidence for HGT of PsaB3 can be seen in sequence 1608 from *Cyanothece* sp. PCC 7425, which despite its name, its more closely related to *Thermosynechococcus*/*Acaryochloris* (Acaryochloridales). It might be the case also for sequence 1580, if its taxonomic placement has been correctly determined.

PsaA

FRL Ancestral -MTISPPES-EKKKVVVDNNPVPTSFEKWAKPGHFDRTLARGPKTTTWIWNLHADAHDFD 58

FRL Fischerella thermalis 7521 -MTLTPERE-QEVRVVVDNDPVPTSFQKWSQPGHFDRTLAKGAKTTTWIWNLHANAHDFD 58

FRL Halomicronema hongdechloris MTTSPPEQR-QRVRVEVDQNPNPTSFEKWAKPGHFERSLARGPKTTTWIWDLHADAHDFD 59

FRL Synechococcus 7335 MTASPPKRN-Q-ASAATEQSPIPTSFERWAKPGHFDRTLARGPKTTTWIWNLHADAHDFD 58

FRL Aphanocapsa GSE MTISPPERG-QKVRVTTDQDPVPTSLEKWAQPGHFDRNLAKGPKTTTWIWNLHANAHDFD 59

FRL Chroococcidiopsis thermalis 7203 -MTITPERE-QKVRVVVDNDPVPTSPELWAKPGHFDRTLARGPKTTTWIWNLHANAHDFD 58

FRL Pleurocapsa 7327 MTITPPKPE-QQVRVVVDNNPVPTSFENWAKPGHFDRTLARGPKTTTWIWNLHANAHDFD 59

VL Fischerella thermalis 7521 MTISPPEREEKKARVIVDNDPVPTSFERWAKPGHFDRTLAKGPKTTTWIWNLHALAHDFD 60

VL Halomicronema hongdechloris MTISPP-EPGRKVKVVVDSDPVNTSFERWAKPGHFERSLSKGPKTTTWIWDLHADAHDFD 59

VL Halomicronema hongdechloris MTTTPREREAN-VKVTVDMDPVPTSFEKWAKPGHFDRTLSRGPKSTTWIWNLHADAHDFD 59

VL Synechococcus 7335 MTTTPREREATKAKVVVDKNPTPTSFERWAKPGHFDRTLARGPKTTTWIWNLHADAHDFD 60

VL Aphanocapsa GSE MTISPPERD-KKVKVVVDRDPVPTSFEKWSKPGHFERTLARGPQTTTWIWNLHALVHDFD 59

VL Chroococcidiopsis thermalis 7203 MTISPPEREEKKARVVVDNDPVPTSFELWSKPGHFDRTLSRGPKTTTWIWNLHALAHDFD 60

VL Pleurocapsa 7327 -MTISPERE-AKVRVRVDNNPVPTSFEKWGKPGHFDRTLARGPKTTTWIWNLHANAHDFD 58

Synechocystis 6803 MTISPPERE-AKAKVSVDNNPVPTSFEKWGKPGHFDRTLARGPKTTTWIWNLHANAHDFD 59

T. elongatus MTISPPERE-PKVRVVVDNDPVPTSFEKWAKPGHFDRTLARGPQTTTWIWNLHALAHDFD 59

. .: .* ** : *.:****:*.*::* ::*****:*** .****

FRL Ancestral SHT-SDLEDISRKIFSAHFGHLAVVFVWLSGMYFHGARFSNYTAWLADPTNIKPSAQVVW 117

FRL Fischerella thermalis 7521 THT-SDLEDISRKIFAAHFGHLAVVFIWLSGMYFHGARFSNFEAWMANPTGIKPSAQVVW 117

FRL Halomicronema hongdechloris SHT-TDLEDISRKIFSAHFGHLAVIFLWLSGMYFHGARFSNFSSWMTDPIHIKPSAQVVW 118

FRL Synechococcus 7335 SHT-NDLQDISRKIFSAHFGHLAVVFVWLSGMYFHGARFSNFSSWMADPTHIRPSAQVVW 117

FRL Aphanocapsa GSE THT-SDLQDISRKIFAAHFGHLAVVFIWLSGMYFHGAKFSNFEAWMTNPTGMKPSAQVVW 118

FRL Chroococcidiopsis thermalis 7203 THT-SDLEDISRKIFAAHFGHLAVIFIWLSGMYFHGAKFSNFEAWMANPTGVKPSAQVVW 117

FRL Pleurocapsa 7327 SHT-SDLEDVSRKIFSAHFGHLAVVFVWLSGMYFHGAKFSNYTAWLADPLHIKPSAQVVW 118

VL Fischerella thermalis 7521 THT-SDLEDISRKIFAAHFGHLAVVTLWLSGMIFHGARFSNYEAWLSDPLNVRPSAQVVW 119

VL Halomicronema hongdechloris SHT-SDLEDISRKIFSAHFGHLAVIFIWLSGMYFHGAKFSNYEAWLSNPTGIKPSAQVVW 118

VL Halomicronema hongdechloris SHT-SDLEDISRKIFSAHFGHLAIVFVWLSGMYFHGARFSNYEAWMGDPTGIQPSAQVVW 118

VL Synechococcus 7335 SHT-SDLEDISRKIFSAHFGHLAVVFIWLSGMYFHGARFSNFEAWMSNPVGIKPSAQVVW 119

VL Aphanocapsa GSE THTDSSLEDVSRKIFSAHFGHLAVVFVWLSGMYFHGAKFSNYEAWMTNPTGIKPSAQVVW 119

VL Chroococcidiopsis thermalis 7203 THT-SDLEDISRKIFAAHFGHLAVIFIWLSGMYFHGARFSNYEAWLADPLGVKPSAQVVW 119

VL Pleurocapsa 7327 SQT-SDLEDISRKIFSAHFGHLAVIFVWLSGMYFHGARFSNYEAWLTDPTTIKPSAQVVW 117

Synechocystis 6803 SQT-SDLEDVSRKIFSAHFGHLAVVFVWLSGMYFHGAKFSNYEGWLADPTHIKPSAQVVW 118

T. elongatus THT-SDLEDISRKIFSAHFGHLAVVFIWLSGMYFHGAKFSNYEAWLADPTGIKPSAQVVW 118

::* ..*:*:*****:*******:: :***** ****:***: .*: :* ::*******

FRL Ancestral PIVGQDILNADVGGGFHGIQITSGLFQLWRASGITNEFQLYCTAIGGLVMAALMLFAGWF 177

FRL Fischerella thermalis 7521 PIFGQEILNGDMGGGFHGIQITSGLFQMWRAAGFTNTFQLYCTAIGGLVMAALMLFAGWF 177

FRL Halomicronema hongdechloris PIFGQEILNADMGDGFRGIQITSGLFQMWRGEGFTHEFQLFWTAIGALVMAALMMFAGWF 178

FRL Synechococcus 7335 PLVGQDILNGDMGGGFRGIQITSGLFQMWRGEGFTNEFQLYCTAIGALVMAGLMIFAGWF 177

FRL Aphanocapsa GSE PIFGQEILNADVGGGFHGIQITSGLFQMWRASGITNSYQLYCTAIGGLVMAALMLFAGWF 178

FRL Chroococcidiopsis thermalis 7203 SLVGQDILNADVGGGFHGIQITSGLFQLWRAAGITNTFQLYCTAIGGLVMAAIMLFAGWF 177

FRL Pleurocapsa 7327 PIFGQDILNADVGGGFHGIQITSGLFQLWRASGITNEFQLFCTAIGGLVMAALMLFAGWF 178

VL Fischerella thermalis 7521 PIVGQDILNGDVGGGFHGIQITSGLFQVWRGWGITNSFQLYCTAIGGLVLAGLLLFAGWF 179

VL Halomicronema hongdechloris PIFGQEILNGDVGGGFHGIQITSGLFQMWRANGITNSFELYCTAIGALVMAGLMLFAGWF 178

VL Halomicronema hongdechloris PIFGQEILNADVGGGFQGIQITSGFFQLWRAAGITNSYQLYVTAIGALVMAGLMLFAGWF 178

VL Synechococcus 7335 PVFGQEILNADVGGGFHGIQITSGLFQMWRASGITNSYQLYCTAIGGLVMAGLMLFAGWF 179

VL Aphanocapsa GSE PIVGQGILNGDVGGGFHGIQITSGLFHLWRASGFTNGFQLYCTAIGGLVMAGLMLFAGWF 179

VL Chroococcidiopsis thermalis 7203 SVVGQDILNADVGGGFHGIQITSGFFQIWRGAGITNTFQLYCTAIGGLVMAALMLFAGWF 179

VL Pleurocapsa 7327 PIVGQGILNADVGGGFSGIQITSGFFYLWRAAGFTNNYQLYCTAIGGLVMAALMLFAGWF 177

Synechocystis 6803 PIVGQGILNGDVGGGFHGIQITSGLFYLWRASGFTDSYQLYCTAIGGLVMAALMLFAGWF 178

T. elongatus PIVGQGILNGDVGGGFHGIQITSGLFQLWRASGITNEFQLYCTAIGGLVMAGLMLFAGWF 178

:.** ***.*:*.** *******:* :**. *:*. ::*: ****.**:*.:::*****

FRL Ancestral HYHVRAPKLEWFQNVESMMNHHLAGLLGLGSLGWAGHQIHVSLPINKLLDAGVA------ 231

FRL Fischerella thermalis 7521 HYHKRAPKLEWFQNTQSMLNHHLAGLLGLGSLGWTGHLIHVSLPTNKLLDTGVA------ 231

FRL Halomicronema hongdechloris HYHVRAPKLDWFRNWESMMNHHLAGLLGLGSLGWAGHLIHVALPTNKLLDAGVP------ 232

FRL Synechococcus 7335 HYHVRSPKLEWFQNVQSMLNHHLAGLLGLGSLGWAGHLIHVALPTNKLLDAGVA------ 231

FRL Aphanocapsa GSE HYHKRAPKLEWFLNWEAMMNHHLAGLLGLGCLGWAGHQIHVALPINKLMDAGVA------ 232

FRL Chroococcidiopsis thermalis 7203 HYHKRAPKLEWFQNWEAMMNHHLAGLLGLGCLGWAGHQIHVALPVNKLLDAGVA------ 231

FRL Pleurocapsa 7327 HYHVRAPKLEWFQNVQAMLNHHLAGLLGLGSLGWAGHQIHVALPINQMLDRGVP------ 232

VL Fischerella thermalis 7521 HYHKRAPKLEWFQNVESMLNHHLQVLLGCGSLGWAGHIIHVSAPTNKLLDAGVA------ 233

VL Halomicronema hongdechloris HYHKKAPKLEWFQNVESMMNHHLAGLLGLGCLGYAGQQIHVSLPINACLDAIDAGKPLTV 238

VL Halomicronema hongdechloris HYHKKAPKLEWFQNVESMMNHHLAGLLGLGCLGWAGHQIHVSLPINKLLDSGVA------ 232

VL Synechococcus 7335 HYHKAAPKLEWFQNVESMLNHHLAGLFGLGSLGWAGHQIHVSLPINKLLDAGVA------ 233

VL Aphanocapsa GSE HYHKAAPKLEWFQNVESMMNHHLAGLLGLGSLGWAGHQIHVSLPINKLLDAGVA------ 233

VL Chroococcidiopsis thermalis 7203 HYHKRAPKLEWFQNVESMLNHHLAGLLGLGSLAWAGHQIHVSLPINKLLDAGVA------ 233

VL Pleurocapsa 7327 HYHVRAPKLEWFQNVESMMNHHLAGLLGLGSLAWAGHQIHVSLPINKLLDAGVA------ 231

Synechocystis 6803 HYHVKAPKLEWFQNVESMMNHHLAGLLGLGSLGWAGHQIHVSMPINKLLDAGVA------ 232

T. elongatus HYHKRAPKLEWFQNVESMLNHHLAGLLGLGSLAWAGHQIHVSLPINKLLDAGVA------ 232

*** :***:** * ::*:**** *:* *.*.::*: ***: * * :*

FRL Ancestral -------PEDIPLPHEFIDASSKMAELYPS----FAQGLRPFFTLNWGVYSDFLTFKGGL 280

FRL Fischerella thermalis 7521 -------LKDIPLPHEFILNPSLMNKLYPHADWGFVKGVVPFFTLQWGHFTDFLTFKGGL 284

FRL Halomicronema hongdechloris -------LEDIPLPHEFILNKSLMVDLYPS----FAEGVKPFFTLNWSAYADFLTFKGGL 281

FRL Synechococcus 7335 -------PQDIPLPHEFVLDKALMAELYPS----FAQGIRPFFTLNWATYSDFLTFNGGL 280

FRL Aphanocapsa GSE -------IKDIPLPHEFILNPTLMTELYPKVDWGYVKGVIPFFTFNWGAYSDFLTFNGGL 285

FRL Chroococcidiopsis thermalis 7203 -------IKDIPLPHEFILNTSLMAELYPS----FAKGLVPFFTLQWGQYADFLTFKGGL 280

FRL Pleurocapsa 7327 -------IEKIPLPHEFILNPSLMQQLYPHVNWGFPSGVIPFFTLNWGQYADFLTFKGGL 285

VL Fischerella thermalis 7521 -------VKDIPLPHEFILNSANLINLYPS----FAKGLAPFFTLNWGVYSDFLTFKGGL 282

VL Halomicronema hongdechloris GGKVIDSVAAIPLPHEWILNPSLMTDIYPS----FAEGLKPFFTLNWSVYADFLTFNGGL 294

VL Halomicronema hongdechloris -------PQNIPLPHEFILDKSLMAELYPS----FAQGLKPFFTLNWGVYADFLTFKGGL 281

VL Synechococcus 7335 -------PQDIPLPHEFILDKALMTELYPS----FAQGLKPFFTLNWAAYSDFLTFKGGL 282

VL Aphanocapsa GSE -------AKDIPLPHEFILNPSLMAELYPKVEWGIPQGVVPFFTFNWGAYSDFLTFKGGL 286

VL Chroococcidiopsis thermalis 7203 -------PKDIPLPQEFILNSNLMTELYPS----FAQGLTPFWTLNWGAYADFLTFKGGL 282

VL Pleurocapsa 7327 -------PKDIPLPHEFILDPSKMAELYPS----FAQGIKPFFTLNWGVYSDFLTFKGGL 280

Synechocystis 6803 -------PKDIPLPHEFILEPSKMAELYPS----FAQGLTPFFTLNWGVYSDFLTFKGGL 281

T. elongatus -------AKDIPLPHEFILNPSLMAELYPKVDWGFFSGVIPFFTFNWAAYSDFLTFNGGL 285

****:*:: : .:** .*: **:*::*. ::*****:***

| ||| |

FRL Ancestral NPVTGGLWLSDTAHHHLAIAVLFIIAGHMYRTNWGIGHSMKEILEAHKGPFMLPFLSFIT 340

FRL Fischerella thermalis 7521 NPVTGGLWLTDVAHHHLAIAVMFIIAGHMYRTNWGIGHSIKEMLDDARTPNMLPFLSFIG 344

FRL Halomicronema hongdechloris NPVTGGLWMTDIAHHHVAIAVLFIIAGHFYRTNWGIGHSFRELLDDARTPKMLPIFNFIG 341

FRL Synechococcus 7335 NPVTGGLWMTDIAHHHVAIAVLFIFAGHMYRTNWGIGHSIRTMLEDARHPKMLPFLSFIG 340

FRL Aphanocapsa GSE NPVTGGLWLSDTAHHHLALAVLFIIAGHFYRTNWGIGHTFRELLDDARTPRMLPLFSFIG 345

FRL Chroococcidiopsis thermalis 7203 NPVTGGLWLSDTAHHHLALAVLFIVAGHFYRTNWGIGHSFKEMLDDAKSPNMLPFLNFIG 340

FRL Pleurocapsa 7327 NPATGGLWLSDTAHHHLAIAVLFIIAGHMYRTNWGIGHSIKEMLDDARTPNMLPFLSFIG 345

VL Fischerella thermalis 7521 NPVTGGLWMTDIAHHHLAIAVLFIIAGHQYRTNWGIGHSIKEILENHKGPF--------T 334

VL Halomicronema hongdechloris NPQTGGLWLTDTAHHHLALAVLFIVAGHFYRTNWGIGHSFKEVLEAHKGP--------VT 346

VL Halomicronema hongdechloris NPVSGGLWLSDTAHHHLALAVLFIVAGHMYRTNWGIGHSMKEILEGHKGDPLL-----FG 336

VL Synechococcus 7335 NPVTGGLWLSDTAHHHLAIAVMFLVAGHMYRTNWGIGHSIKQILDGHKGDPLL-----FG 337

VL Aphanocapsa GSE NPVTGGLWLSDTAHHHLAIAVLFIIAGHMYRTNWGIGHSMREILEAHKGPF--------T 338

VL Chroococcidiopsis thermalis 7203 NPVTGGLWLTDQAHHHLAIAVLFIIAGHMYRTNWGIGHSLKEILENHKGPF--------T 334

VL Pleurocapsa 7327 NPVTGGLWLSDTAHHHLAIAVLFIIAGHMYRTNWGIGHSMKEILEAHKGPF--------T 332

Synechocystis 6803 NPVTGGLWLSDTAHHHLAIAVLFIIAGHMYRTNWGIGHSMKEILEAHKGPF--------T 333

T. elongatus NPVTGGLWLSDTAHHHLAIAVLFIIAGHMYRTNWGIGHSLKEILEAHKGPF--------T 337

** :****::* ****:*:**:*:.*** *********::: :*: :

|

FRL Ancestral GEGHKGLYEILTTSWHAQLAINLAMLGSLSIIVAHHMYAMPPYPYLATDYATTLSLFTHH 400

FRL Fischerella thermalis 7521 PVGHKGLFEVLTTSWHAQLSINLAMLGSLSIIIAHHMYAMPPYPYLATDYGTVVSLFTHH 404

FRL Halomicronema hongdechloris PVGHRGLDKIFETSWHANLAIHLVQFGTASLLVAHHMYAMPPYPYLATDYATVTSLFTHH 401

FRL Synechococcus 7335 PVGHRGLFEVLTTSWHAQLSINLAMMGSLSIIVAQHMYSMPPYPYLATDYGTVTSLFTHH 400

FRL Aphanocapsa GSE PVGHKGLDRIFETSWHANLSIHLVQFGTASLLAAHHMYAMPPYPYLATDYATVTSLFTHH 405

FRL Chroococcidiopsis thermalis 7203 PVGHEGLDKIFETSWHANLSIHLVQFGTASLLVAHHMYAMPPYPYLATDYATALSLFTHH 400

FRL Pleurocapsa 7327 PEGHKGLFETLTTSWHAQLSINLAMLGSLSIIVAHHMYAMPPYPYLATDYATVLSLFTHH 405

VL Fischerella thermalis 7521 GDGHKGLYENMTTSWHAQLGTNLAMLGSLTIIVAHHMYAMPPYPYLATDYATQLCIFTHH 394

VL Halomicronema hongdechloris GEGHKGMYEIFTTSWHCQLSWNLAWIGSLSILVAHHMYSMPPYPYIATDYPTQLSLFTHH 406

VL Halomicronema hongdechloris GKGHDGLYENLTTSWHAQLAVNLALLGSLTIIVAHHMYAMPPYPYIATDYPTQLSLFTHH 396

VL Synechococcus 7335 GEGHVGMYEFLTQSWHAQLAINLALGGSVTIIVAQHMYAMPPYPYLATDYGTQLSLFTHH 397

VL Aphanocapsa GSE GEGHKGLYQVLTTSWHAQLALNLAMLGSLTIIVAHHMYSMPPYPYLATDYPTQLSLFTHH 398

VL Chroococcidiopsis thermalis 7203 GDGHRGLFENMTTSWHAQLGTNLAMLGSLTIIVAHHMYAMPPYPYLATDYATQLSIFTHH 394

VL Pleurocapsa 7327 GEGHKGLYEILTTSWHAQLAINLALLGSLSIIVAHHMYAMPPYPYMAIDYATQLACFTHH 392

Synechocystis 6803 GEGHKGLYEILTTSWHAQLAINLALLGSLTIIVAQHMYAMPPYPYQAIDYATQLSLFTHH 393

T. elongatus GAGHKGLYEVLTTSWHAQLAINLAMMGSLSIIVAQHMYAMPPYPYLATDYPTQLSLFTHH 397

** *: . : ***.:*. :*. *: ::: *:***:****** * ** * . ****

| |

FRL Ancestral VWIGGFLIVGAAAHAAIFMVRDYDPAENVNNLLDRVLRHRDAIISHLVWVCQFLGFHSFG 460

FRL Fischerella thermalis 7521 VWIGGFLIVGGAAHAAIYMVRDYDPEQNFNNVLDRVLRHRDAIISHLAWVCQFLGFHSFA 464

FRL Halomicronema hongdechloris VWIAGFCIVGGAAHAAIFLVRDYNPADHVNNVLDRTLRHRDTVVSHLAWVCQFLGFHSFA 461

FRL Synechococcus 7335 MWIGGFLIVGAAAHAGIFMVRDYDPAENVNNVLDRVLRHRDAIISHLVWVCQFLGFHSFA 460

FRL Aphanocapsa GSE MWIAGFCIVGGAAHAAIFMVRDYNPADHVNNVLDRVLRHRDAIISHLAWVCQFLGFHSFA 465

FRL Chroococcidiopsis thermalis 7203 VWIAGFCIVGGAAHAAIFMVRDYDPAHHVNNILDRTLRHRDVIISHLAWVCQFLGFHSFA 460

FRL Pleurocapsa 7327 VWIGGFLIVGAAAHAAIYMVRDYDPAKNVNNVLDRVIRHRDAIISHLAWVCQFLGFHSFA 465

VL Fischerella thermalis 7521 MWIGGFLIVGGAAHAAIFMVRDYDPVVNQNNVLDRVIRHRDAIISHLNWVCIFLGFHSFG 454

VL Halomicronema hongdechloris MWIGGFLIVGAGAHAAIFMVRDYDPATHINNLLDRVIRHRDAIISHLNWVCIFLGFHSFG 466

VL Halomicronema hongdechloris MWIGGFLVVGAGAHAAIFMVRDYDPGVNLDNALDRMIRSRDAIISHLNWVCIFLGFHSFG 456

VL Synechococcus 7335 MWIGGFLVVGAGAHGAIALIRDYDPAKHVNNVLDRVLRVRDAIISHLNWVCIFLGFHSFG 457

VL Aphanocapsa GSE MWIGAFCIVGGAAHGAIYMVRDYDPVVNQNNLLDRVLRHRDAIISHLNWVCIFLGFHSFG 458

VL Chroococcidiopsis thermalis 7203 MWIGAFCIVGGAAHATIFMVRDYDPATNMNNVLDRVLRHRDAIISHLNWVCMFLGFHSFG 454

VL Pleurocapsa 7327 VWIGGFLIVGAGAHAAIFMVRDYDPAKNVDNLLDRVIRHRDAIISHLNWVCIFLGFHSFG 452

Synechocystis 6803 MWIGGFLIVGAGAHGAIFMVRDYDPAKNVNNLLDRMLRHRDAIISHLNWVCIFLGFHSFG 453

T. elongatus MWIGGFLVVGGAAHGAIFMVRDYDPAMNQNNVLDRVLRHRDAIISHLNWVCIFLGFHSFG 457

:**..* :**..**. * ::***:* : :* *** :* **.::*** *** *******.

|| |||||||||

FRL Ancestral IYCHNDTMRAFGRPQDMFSDTGIQLQPIFAQWIQNIHTMAPGN--LEAAQPLGNVFGGLN 518

FRL Fischerella thermalis 7521 MYCHNDTMRAFGRPQDMFSDTGIQLQPVFAQWLQHIHTMTIGNPSLQVAAPLGHAFGGLR 524

FRL Halomicronema hongdechloris MYCHNDTMRAFGRPQDMFSDTGIQLQPIFAQWVQQIQTMAVGA-NLQAAEPLGNVFGGLR 520

FRL Synechococcus 7335 MYCHNDTMRAFGRPQDMFSDTGIQLQPIFAQWVQHIQTMAVGS--AQVAEPLGDALGGIQ 518

FRL Aphanocapsa GSE MYCHNDTMRAFGRPQDMFSDTGIQLQPVFAQWIQHIHTAAVGA--AQAAQPLGDVFGGLH 523

FRL Chroococcidiopsis thermalis 7203 MYCHNDTMRAFGRPQDMFSDTGIQLQPIFAQWIQHIHTAAVGA--AQVAQPLGDVFGGVR 518

FRL Pleurocapsa 7327 MYCHNDTMRAFGRPQDMFSDTGIQLQPVFAQWIQHVHTAAVGV--GQAAQPLGNVFGGLR 523

VL Fischerella thermalis 7521 LYVHNDTMRALGRPQDMFSDTAIQLQPVFAQWVQNLHTLAPGA----------------- 497

VL Halomicronema hongdechloris LYVHNDTMRAFGRPQDMFSDTGIQLQPVFAQWVQNLHAAAA------------------- 507

VL Halomicronema hongdechloris LYIHNDTMRALGRPQDMFSDSAIQLQPVFAQWVQNLHAAAA------------------- 497

VL Synechococcus 7335 LYIHNDTMQALGRPQDMFSDTAIQLQPVFAQWIQSLHTAAPSI----------------- 500

VL Aphanocapsa GSE LYVHNDTMRAFGRPQDLFSDTGIQLQPVFAQWIQNLHTLAPGT----------------- 502

VL Chroococcidiopsis thermalis 7203 LYIHNDTMQALGRPQDMFSDTAIQLQPVFAQWVQNLHTLAPGS----------------- 497

VL Pleurocapsa 7327 LYVHNDTMRAFGRPQDMFSDTGIQLQPIFAQWVQNIHALAPGN----------------- 496

Synechocystis 6803 LYIHNDTMRALGRPQDMFSDTAIQLQPIFAQWVQHLHTLAPGA----------------- 497

T. elongatus LYVHNDTMRAFGRPQDMFSDTGIQLQPVFAQWVQNLHTLAPGG----------------- 501

:* *****:*:*****:***:.*****:****:* ::: :

|| | |

FRL Ancestral NIALGGLGTTAPGLSEPVSYAFGGGVVAVGGKVAMMPITLGTADFLIHHIHAFTIHVTVL 578

FRL Fischerella thermalis 7521 NLELTGLGTAAPNLHDPVSYAFGGGVVAVGGKVAMMPITLGTADFLIHHIHAFTIHVTVL 584

FRL Halomicronema hongdechloris NIDLAGVGVTAPGLGGPVSHAFGGGVVAIGDKIAMMPIQLGTADFLIHHIHAFTIHVTVL 580

FRL Synechococcus 7335 NIALSGVGTTAPGVASPASYAFGGGLVAVGGKVAMMPISLGTADFLIHHIHAFTIHVTVL 578

FRL Aphanocapsa GSE NIQLSGLGTTAPHIMGPVSYAWGGGVVAVAGKVAMMPIPLGTADFMIHHIHAFTIHVTAL 583

FRL Chroococcidiopsis thermalis 7203 GIELSGLGTTAPGIGAPVSYAWGGGMVAVGGKVAMMPIALGTADFLIHHIHAFTIHVTVL 578

FRL Pleurocapsa 7327 NIELSGLGTTAPGLSEPVSYAFGGGVVAVGSKIAMMPITLGTADFLIHHIHAFTIHVTVL 583

VL Fischerella thermalis 7521 ---------TAPNALEPVSYAFGGGILAVGGKVAMMPIALGTADFMIHHIHAFQIHVTTL 548

VL Halomicronema hongdechloris -------GGTAPNAAAGVSPAFGGDILAVVGKVAMMPITLGTADFLVHHIHAFTIHVTVL 560

VL Halomicronema hongdechloris -------GSTAPTALAGVSPAFGGDAVAVAGKVAMMPITLGTADFMVHHIHAFTIHVTVL 550

VL Synechococcus 7335 -------SGTAPNALAPVSYAFGGDVVAVGGKVAMMPITLGTADFMVHHIHAFTIHVTVL 553

VL Aphanocapsa GSE ---------TAPNAVEPVSYAFGGGVLAVGGKVAMMPIALGTADFMVHHIHAFTIHVTVL 552

VL Chroococcidiopsis thermalis 7203 ---------TAPNALEPVSYAFGGGVLAVGGKVAMMPIALGTADFMIHHIHAFQIHVTVL 548

VL Pleurocapsa 7327 ---------TAPNALAPASYAFGGDVVAVGGKVAMMPIALGTADFLVHHIHAFTIHVTVL 546

Synechocystis 6803 ---------TAPNALATASYAFGGETIAVAGKVAMMPITLGTADFMVHHIHAFTIHVTAL 547

T. elongatus ---------TAPNAAATASVAFGGDVVAVGGKVAMMPIVLGTADFMVHHIHAFTIHVTVL 551

** .* *:** :*: .*:***** ******::****** ****.*

FRL Ancestral ILLKGVLYARSSRLIPDKAELGFRFPCDGPGRGGTCQVSAWDHVFLGLFWMYNSLSIVIF 638

FRL Fischerella thermalis 7521 VLLKGVLFARSSRLIPDKANLGFRFPCDGPGRGGTCQVSAWDHVFLGLFWMYNSLSMVIF 644

FRL Halomicronema hongdechloris VLLKGVLFSRNSRLIPDKGELGFRFPCDGPGRGGTCQVSAWDHVFLGLFWMYNSLSIVIF 640

FRL Synechococcus 7335 VLLKGVLFARNSRLIPDKSELGFRFPCDGPGRGGTCQVSAWDHVFLGLFWMYNSIAMVIF 638

FRL Aphanocapsa GSE VLLKGVLFARNSRLIPDKASLGFRFPCDGPGRGGTCQVSAWDHVFLGLFWMYNSFSMIIF 643

FRL Chroococcidiopsis thermalis 7203 VLFKGVLFARGSRLVPDKANLGFRFPCDGPGRGGTCQVSAWDHVFLGLFWMYNSLSMVVF 638

FRL Pleurocapsa 7327 VLLKGVLYARNSRLIPDKSELGFRFPCDGPGRGGTCQVSAWDHVFLGLFWMYNSLSIVIF 643

VL Fischerella thermalis 7521 ILLKGFLFARSSRLIPDKANLGFRFPCDGPGRGGTCQVSGWDHVFLGLFWMYNTISIAIF 608

VL Halomicronema hongdechloris ILLKGVLFARNSRLIPDKGELGFRFPCDGPGRGGTCQVSGWDHVFLGLFWMYNSLSIVIF 620

VL Halomicronema hongdechloris ILLKGVLFARNSRLIPDKGELGFRFPCDGPGRGGTCQVSGWDHVFLGLFWMYNSLSIVIF 610

VL Synechococcus 7335 ILLKGVLYARNSRLIPDKSELGFRFPCDGPGRGGTCQVSAWDHVFLGLFWMYNSISIVIF 613

VL Aphanocapsa GSE ILLKGVLFARSSRLVPDKANLGFRFPCDGPGRGGTCQVSGWDHVFLGLFWMYNCISIVIF 612

VL Chroococcidiopsis thermalis 7203 ILLKGFLFARNSRLIPDKANLGFRFPCDGPGRGGTCQVSGWDHVFLGLFWMFNTISIAVY 608

VL Pleurocapsa 7327 ILLKGVLYARSSRLIPDKSELGFRFPCDGPGRGGTCQVSGWDHVFLGLFWMYNSLSIVIF 606

Synechocystis 6803 ILLKGVLYARSSRLVPDKANLGFRFPCDGPGRGGTCQVSGWDHVFLGLFWMYNSLSIVIF 607

T. elongatus ILLKGVLFARSSRLIPDKANLGFRFPCDGPGRGGTCQVSGWDHVFLGLFWMYNCISVVIF 611

:*:**.*::*.***:***..*******************.***********:* ::: ::

FRL Ancestral HFFWKMQSDVWGTVDADGTVNHITAGNFAQSSITINGWLRDFLWAQASQVITSYNSALSA 698

FRL Fischerella thermalis 7521 HFFWKMQSDVWGTVGADGVVTHITGGNFATSSITNNGWLRDFLWAQSTQVITSYNTSLSA 704

FRL Halomicronema hongdechloris HFFWKMQSDVWGTVGADGTISHITGGNFAQASITNNGWLRDFLWAQASQVIGSYGSALSA 700

FRL Synechococcus 7335 HFFWKMQSDVWGAVDANGTVSHITGGNFAQSSITINGWLRDFLWAQATQVISSYGSALSA 698

FRL Aphanocapsa GSE HFFWKMQSDVWGTVSADGTISHITGGNFAVSSLTNNGWLRDFLWAQASQVITSYNSALSA 703

FRL Chroococcidiopsis thermalis 7203 HFSWKMQSDVWGTVDSDGIVTHLTGGNFATSSITNNGWLRDFLWAQSAQVIQSYNSSLSA 698

FRL Pleurocapsa 7327 HFFWKMQSDVWGTVDADGSITHITLGNWAQSSITNNGWLRDFLWAQATQAITSYGGALSA 703

VL Fischerella thermalis 7521 HFSWKMQSDVWGTVDAAGNVSHVTGGNFAQSAITINGWLRDFLWAQATQVINSYGSALSA 668

VL Halomicronema hongdechloris HFSWKMQSDVWGSVSPDGSVSHITAGNFAQSAITINGWLRDFLWAQASQVIGSYGSALSA 680

VL Halomicronema hongdechloris HFSWKMQSDVWGSVSPDGSVSHITAGNFAQSAITINGWLRDFLWAQASQVIGSYGSALSA 670

VL Synechococcus 7335 HFSWKMQSDVWGTVSDNGTVSHITGGNFAASATTINGWLRDFLWAQASQVINTYGSALSA 673

VL Aphanocapsa GSE NFSWKMQSDVWGTVAPDGTVSHITGGNFAQSALTINGWLRDFLWAQASQVIGSYGSALSA 672

VL Chroococcidiopsis thermalis 7203 HFSWKMQSDVWGTVDPDGTINHITAGNWALSATTINGWLRDFQWAQAAQVIQSYGSALSA 668

VL Pleurocapsa 7327 HFSWKMQSDVWGTVAPDGTVTHVTLGNWAQSAITINGWLRDFLWAQAAQVINSYGSALSA 666

Synechocystis 6803 HFSWKMQSDVWGTVSPDGSVTHVTLGNFAQSAITINGWLRDFLWAQAANVINSYGSALSA 667

T. elongatus HFSWKMQSDVWGTVAPDGTVSHITGGNFAQSAITINGWLRDFLWAQASQVIGSYGSALSA 671

:* *********:* * :.*:* **:* :: * ******* ***:::.* :*. :***

FRL Ancestral YGLMFLAGHFVFAFSLMFLFSGRGYWQELIESIVWAHNKLKIAPAIQPRALSITQGRAVG 758

FRL Fischerella thermalis 7521 YGLMFLGGHFIFGFSLMFLFSGRGYWQELIESIVWAHNKLKVAPAIQPRALSIIHGRAVG 764

FRL Halomicronema hongdechloris YGLFFLAGHFIFGFSLMFLFSGRGYWQELIESIVWAHNKLKITTAIQPRALSITQGRAVG 760

FRL Aphanocapsa GSE YGLLFLGGHFVFGFSLMFLFSGRGYWQELIESIVWAHNKLKVAPVIQPRALSITHGRAVG 763

FRL Chroococcidiopsis thermalis 7203 YGLMFLAGHFIFGFSLMFLFSGRGYWQELIESIVWAHNKLKVAPAIQPRALSIVHGRAVG 758

FRL Synechococcus 7335 YGLMFLAGHFVFAFSLMFLFSGRGYWQELIESIVWAHNKLRITTAIQPRALSITQGRAVG 758

FRL Pleurocapsa 7327 YGLLFLGGHFIFGFSLMFLFSGRGYWQELIESIVWAHNKLKITPAIQPRALSIIHGRAVG 763

VL Fischerella thermalis 7521 YGLMFLGAHFIWAFSLMFLFSGRGYWQELIESIVWAHNKLKVAPAIQPRALSIIQGRAVG 728

VL Halomicronema hongdechloris YGLLFLGAHFVWAFSLMFLFSGRGYWQELIESIVWAHNKLKVAPAIQPRALSITQGRAVG 740

VL Halomicronema hongdechloris YGLLFLGAHFVWAFSLMFLFSGRGYWQELIESIVWAHNKLKVAPAIQPRALSIVQGRAVG 730

VL Synechococcus 7335 YGLMFLGAHFVWAFSLMFLFSGRGYWQELIESIVWAHNKLKVAPAIQPRALSITQGRAVG 733

VL Aphanocapsa GSE YGLLFLGAHFVWAFSLMFLFSGRGYWQELIESIVWAHNKLKVAPAIQPRALSIIQGRAVG 732

VL Chroococcidiopsis thermalis 7203 YGLLFLGAHFVWAFSLMFLFSGRGYWQELIESIVWAHNKLKVAPTVQPRALSIIQGRAVG 728

VL Pleurocapsa 7327 YGIMFLAGHFVFAFSLMFLFSGRGYWQELIESIVWAHNKLKVAPAIQPRALSIIQGRAVG 726

Synechocystis 6803 YGIMFLAGHFVFAFSLMFLFSGRGYWQELIESIVWAHNKLNVAPAIQPRALSIIQGRAVG 727

T. elongatus YGLLFLGAHFIWAFSLMFLFSGRGYWQELIESIVWAHNKLKVAPAIQPRALSIIQGRAVG 731

**::**..**::.***************************.:: .:******* :*****

FRL Ancestral VAHYLLGGIVTTWAFFLARMAAIG 782

FRL Fischerella thermalis 7521 VAHYLLGGIVTTWAFFLARMTAFG 788

FRL Halomicronema hongdechloris VAHYLLGGIVTTWAFFLARMAAIG 784

FRL Synechococcus 7335 AAHYLLGSIVTTWAFFLARMAAIG 782

FRL Aphanocapsa GSE VAHYLLGGIVTTWAFFLARMAALG 787

FRL Chroococcidiopsis thermalis 7203 VAHYLLGGIVTTWAFFLARMSAIG 782

FRL Pleurocapsa 7327 VAHYLLGGIVTTWAFFLARMAAIG 787

VL Fischerella thermalis 7521 VAHYLLGGIATTWAFFHAHILSIG 752

VL Halomicronema hongdechloris VAHFLLGGIATTWAFFLARIIAVG 764

VL Halomicronema hongdechloris VAHFLLGGIATTWAFFLARIIAVG 754

VL Synechococcus 7335 VAHYLLGGIATTWAFFLARMLSVG 757

VL Aphanocapsa GSE VAHYLLGGIATTWAFFLARIISVG 756

VL Chroococcidiopsis thermalis 7203 VAHYLLGAIVTIWAFFEARILSVG 752

VL Pleurocapsa 7327 VAHYLLGGIVTTWAFFHARTLSF- 749

Synechocystis 6803 VAHYLLGGIVTTWAFFLARSLSIG 751

T. elongatus VAHYLLGGIATTWAFFLARIISVG 755

.**:***.*.* **** *: :.

PsaB

FRL Ancestral MATKFPKFSQDLQQDPTTRRIWYAIATAHDFESHDGMTEENLYQKIFASHFGHLAIIFLW 60

FRL Fischerella thermalis 7521 MATKFPKFSQDLANDPTTRRIFYAIATAHDFESHDGMTEENLYQRIFASHFGHLAIIFLW 60

FRL Halomicronema hongdechloris MATKFPKFSQDLQRDPTTRRLFYAIATAHDFESHDGMSEENLYQRIFASHFGHLAIIFLW 60

FRL Synechococcus 7335 MATKFPKFSQELQQDPTTRRIFYSLATAHDFESHDGMTEESLYQRIFASHFGHLAIIFLW 60

FRL Aphanocapsa GSE MATKFPKFSQDLAQDPTTRRIFYAIATAHDFETHDGMTEENLYQKIFSSHFGHLAIIFLW 60

FRL Chroococcidiopsis thermalis 7203 MATKFPKFSQDLAQDPTTRRIWYAMATAHDFELHDGMTEENLYQKIFASHFGHLAIIFLW 60

FRL Pleurocapsa 7327 MATKFPKFSQDLQQDPTTRRIWYAIATAHDFESHDGMTEENIYQRIFASHFGHIAIIFLW 60

VL Fischerella thermalis 7521 MATKFPKFSQDLAQDPTTRRIWYAMAMGNDFESHDGMTEENLYQKIFATHFGHLAIIFLW 60

VL Halomicronema hongdechloris MATKFPKFSQDLASDPTTRRIWYGIATAHDFESHDGMTEENLYQKIFASHFGHLAIIFLW 60

VL Synechococcus 7335 MATKFPKFSQALAQDPTTRRIWYGIATAHDFETHDGMTEENLYQKIFASHFGHLAIIFLW 60

VL Aphanocapsa GSE MATKFPKFSQDLAQDPTTRRIWYAIATAHDFESHDGMTEENLYQKIFASHFGHLAIIFLW 60

VL Chroococcidiopsis thermalis 7203 MATKFPKFSQDLAQDPTTRRIWYGIATAHDFESHDGMTEENLYQKIFATHFGHIAIIFLW 60

VL Chroococcidiopsis thermalis 7203 MATKFPKFSQDLAQDPTTRRIWYGIATAHDFESHDGMTEENLYQKLFATHFGHLAIIFLW 60

VL Chroococcidiopsis thermalis 7203 MATKFPKFSQDLAQDPTTRRLWYGIAQAHDFESHDGMTEENLYQKIFGTHFAQVAIIFLW 60

VL Pleurocapsa 7327 MATKFPKFSQDLQQDPTTRRIWYGIATAHDFESHDGMTEENLYQKIFASHFGHIAIIFLW 60

Synechocystis 6803 MATKFPKFSQDLAQDPTTRRIWYGIATAHDFETHDGMTEENLYQKIFASHFGHIAIIFLW 60

T. elongatus MATKFPKFSQDLAQDPTTRRIWYAIAMAHDFESHDGMTEENLYQKIFASHFGHLAIIFLW 60

********** * ******::*.:* .:*** ****:**.:**::*.:**.::******

| |

FRL Ancestral TSGILFHVAWQGNFEQWIKDPLNVRPIAHAIWDAQFGPPAIEAFTQAGASNPVDICYSGV 120

FRL Fischerella thermalis 7521 ASGILFHVAWQGNFEVWIKDPVHVRPIAHAIWDAQFGSGAIKAFTQAGARNPVDICYSGV 120

FRL Halomicronema hongdechloris ISGILFHVAWQGNFEQWIQDPLNNSPIAHAIWDAQFGPPAIAAYTQAGAMNPVDICYSGV 120

FRL Synechococcus 7335 TSGILFHVAWQGNFEAWIKDPLNISPIAHAIWDPQFGPAAMDAFTPAGAGNPVNFCYSGV 120

FRL Aphanocapsa GSE ASGTLFHVAWQGNFELWIKDPLTVSPIAHAIWDAQFGPATIEAYTQAGAHNPVDICYSGV 120

FRL Chroococcidiopsis thermalis 7203 ASGVLFHVAWQGNFEQWIKDPLNVRPIAHAIWDAQFGPPAIEAFTRAGATNPVDICYSGV 120

FRL Pleurocapsa 7327 TSGILFHVAWQGNFEQWVKDPLTVRPIAHAIWDAQFGAPAIQAYTQAGASNPVDICTSGV 120

VL Fischerella thermalis 7521 ASSLLFHVAWQGNFEQWIKDPLHIRPIAHAIWDPHFGKPAIEAFTQGGASNPVNIAYSGV 120

VL Halomicronema hongdechloris TSGNLFHVAWQGNFQQWVKDPLNISPIAHAIWDPQFGQSAVEAFSQAGANYPVDIAYSGV 120

VL Synechococcus 7335 TSGNLFHVAWQGNFPQWTQDPLNVKPIAHAIWDPHFGQPAVDAFSQAGSTSPVNIAYSGV 120

VL Aphanocapsa GSE TSGSLFHVAWQGNFEQWIKDPLNIRPIAHAIWDPQFGKGAIDAFTQAGASGPVDIAYSGV 120

VL Chroococcidiopsis thermalis 7203 ASSLLFHVAWQGNFEQWIKDPLHVRPIAHAIWDPQFGKAAVDAFTQGGASYPVDISYSGV 120

VL Chroococcidiopsis thermalis 7203 ASSLLFHVAWQGNFEQWIKDPLHVRPIAHAIWDPQFGKAAVDAFTQGGASYPVNIAYSGV 120

VL Chroococcidiopsis thermalis 7203 TSSLLFHVAWQGNFEQWIKDPLHVRPIAHAIWDPHFGKAAVDAFTQGGVNYPVNIAYSGV 120

VL Pleurocapsa 7327 TSGTIFHVAWQGNFEQWIKDPLNVRPIAHAIWDPQFGKAAVDAFTQGGASYPVDISYSGV 120

Synechocystis 6803 TSGTLFHVAWQGNFEQWIKDPLNIRPIAHAIWDPHFGEGAVNAFTQAGASNPVNIAYSGV 120

T. elongatus VSGSLFHVAWQGNFEQWVQDPVNTRPIAHAIWDPQFGKAAVDAFTQAGASNPVDIAYSGV 120

*. :********* * :**: ******** :** :: *:: .* **::. ***

FRL Ancestral YHWWYTIGMRTNNDLYVGAIFLILLAAVFLFAGWLHLQPRFRPSLSWFKNAESRLNHHLA 180

FRL Fischerella thermalis 7521 YHWWYTIGLRTNTELYVGALFLILLAAVFLFAGWLHLQPRYRPNLGWFKNSEARLNHHLA 180

FRL Halomicronema hongdechloris YHWWYTIGMRTNNDLFMGSIFLLLLSSVMLYAGWLHLQPRFRPGLAWFKNAESRLNHHLA 180

FRL Synechococcus 7335 YHWWYTIGLRTNGDLFAGAMFLLLLAAVMLYAGWLHLQPRYRPSLAWFKNAESRLNHHLA 180

FRL Aphanocapsa GSE YHWWYTIGMRTNNDLFVGSLFLILLASFMLFAGWLHLQPKFRPALSWFKNAESRLNHHLA 180

FRL Chroococcidiopsis thermalis 7203 YHWWYTIGMRTNNELYVGAIFLLLLAALFLFAGWLHLQPRYRPTLGWFKSAEPRLNHHLA 180

FRL Pleurocapsa 7327 YHWWYTIGMRTNNDLYAGAIFLILLAAVFLYAGWLHLQPRFRPSLSWFKNAESRLNHHLA 180

VL Fischerella thermalis 7521 YHWWYTIGMRTNGDLYMGSIFLLVLSSLFLFAGWLHLQPKFRPSLAWFKMAESRLNHHLA 180

VL Halomicronema hongdechloris YHWWYTIGMRTAGDLYGGALFLMIMAAVFLFAGWLHLQPRFRPSLAWFKNAESRMNHHLA 180

VL Synechococcus 7335 YHWWYTIGMRTNGDLYAGAVGLLIFAAVMLFAGWLHLQPKFRPSLSWFKNAESRLNHHLA 180

VL Aphanocapsa GSE YHWWYTIGMRTNSDLYMGAITLLVFSSLFLFAGWLHLQPKFRPSLAWFKNAESRLNHHLS 180

VL Chroococcidiopsis thermalis 7203 YHWWYTIGMRTNNDLYMGSVFLLVLASVFLLAGWLHLQPKFRPGLAWFKSAESRLNHHLA 180

VL Chroococcidiopsis thermalis 7203 YHWWYTIGMRTNNDLYMGSVFLLLLASLFLFAGWLHLQPKFRPSLSWFKSAEPRLNHHLA 180

VL Chroococcidiopsis thermalis 7203 YHWWYTIGMRTNGDLYMGAVFLLLLASLFLYAGWLHLQPKFRPSLSWFKSAEPRLNHHLA 180

VL Pleurocapsa 7327 YHWFYTIGMRTNGDLYQGSIFLLILSALFLFAGWLHLQPKFRPSLSWFKNAESRLNHHLA 180

Synechocystis 6803 YHWFYTIGMTTNQELYSGAVFLLVLASLFLFAGWLHLQPKFRPSLAWFKNAESRLNHHLA 180

T. elongatus YHWWYTIGMRTNGDLYQGAIFLLILASLALFAGWLHLQPKFRPSLSWFKNAESRLNHHLA 180

***:****: * :*: *:: *:::::. * ********::** *.*** :* *:****:

FRL Ancestral GLFGVSSLAWAGHLIHVAIPESRGQHVGWDNFLSTPPHPAGLAPFFTGNWGAYAQNPDTA 240

FRL Fischerella thermalis 7521 GLFGVSSLAWAGHLVHVAIPESRGQHVGWDNFLSTPPHPAGLWAFFTGNWGAYAQNPDTA 240

FRL Halomicronema hongdechloris GLFGVSSLAWTGHLVHVALPESRGQHVGWDNFLSIRPHPEGLAPLFTGNWGAYAQNPDTA 240

FRL Synechococcus 7335 GLFGVSSLAWTGHLVHVAIPESRGQHVGWDNFLSMPPHPEGLKPFFTGNWGAYALNPDTS 240

FRL Aphanocapsa GSE GLFGVSSLAWTGHLVHVAIPESRGQHVGWDNLLSAAPHPGGLLPFFTGNWTAYAQNPDTA 240

FRL Chroococcidiopsis thermalis 7203 GLFGVSSLAWAAHLIHVAIPESRGQHVGWDNFLFTPPHPAGLGAFFTGNWSAYAQNPDTA 240

FRL Pleurocapsa 7327 GLFGVSSLAWAGHLIHVAIPESRGQHVGWDNFLTTKPHPAGLGPFFTGNWGVYAQNPDTA 240

VL Fischerella thermalis 7521 GLFGVSSLAWTGHLVHVAIPESRGQHVGWDNFLSTLPHPAGLQPFFTGNWGVYAENPDTA 240

VL Halomicronema hongdechloris GLFGVSSLAWAGHLIHVAIPESRGQHVGWDNFLFTPPHPAGLTPFFTGNWGVYAQNPDTA 240

VL Synechococcus 7335 GLFGVSSLAWTGHLVHVAIPESRGVHVGWDNFLTMKPHPAGLQPFFTGNWGAYAQNPDTA 240

VL Aphanocapsa GSE ALFGVSSLAWAGHLIHVAIPESRGQHVGWDNFLSTMPHPAGLAPFFTGNWAVYAQNPDTA 240

VL Chroococcidiopsis thermalis 7203 GLFGVSSLAWTGHLVHVAIPEARGQHVGWSNFLTTPPHPEGLTPFFTGKWAAYAANPDTA 240

VL Chroococcidiopsis thermalis 7203 GLFGVSSLAWTGHLVHVAIPESRGQHVGWSNFLTTPPHPDGLQPFFSGNWGAYAANPDTA 240

VL Chroococcidiopsis thermalis 7203 GLFGVSSLAWAGHLIHVAIPESRGQHVGWSNFLTTPPHPEGLRPFFTGDWGAYAVSPDTA 240

VL Pleurocapsa 7327 GLFGVSSLAWTGHLVHVAIPESRGQHVGWDNFLSTAPHPAGLLPFFTGNWGVYAQNPDTA 240

Synechocystis 6803 GLFGVSSLAWAGHLVHVAIPEARGQHVGWDNFLSTPPHPAGLMPFFTGNWGVYAADPDTA 240

T. elongatus GLFGVSSLAWAGHLIHVAIPESRGQHVGWDNFLSTMPHPAGLAPFFTGNWGVYAQNPDTA 240

.*********:.**:***:**:** ****.*:* *** ** :*:*.* .** .***:

FRL Ancestral EHVFGTSQGAGTAILTFLGGFHPQTESLWLTDMAHHHLAIAVIFIIAGHMYRTNWGIGHN 300

FRL Fischerella thermalis 7521 EHVFSTSQGAGTAILTFLGGFHPQTQSLWLTDMAHHHLAIAVVLIIAGHMYRTNWRIGHS 300

FRL Halomicronema hongdechloris EHAFGTAQGAGSAILTFLGGFHPQTESLWLTDMAHHHLAIAVIFIVAGHMYRTNFGIGHN 300

FRL Synechococcus 7335 EHLFNTSQGAGTAILTFLGGFHPQTESLWLTDMAHHHLAIAVIFIIAGHMYRTNFGIGHS 300

FRL Aphanocapsa GSE EHLFGTSTGAGTAILTFLGGFHPQTESLWLTDMAHHHLAIAVLFIVAGHMYRTNWGIGHN 300

FRL Chroococcidiopsis thermalis 7203 QHVFNSSQGAGTAILTFLGGFHPQTQSLWLTDMAHHHVAIAVLFIIAGHMYRTNWGIGHS 300

FRL Pleurocapsa 7327 EHIFNTSQGAGTAILTFLGGFHPQTESLWLTDMAHHHLAIAVIFIIAGHMYRTNWGIGHN 300

VL Fischerella thermalis 7521 SHVFGTSQGAGTAILTFLGGFHPQTESLWLTDMAHHHLAIAVLFIVAGHMYRTNFGIGHS 300

VL Halomicronema hongdechloris SHVFGSSTGAGSAILTFLGGFHPQTESLWLTDMAHHHLAIAVLFIVAGHMYRTNFGIGHN 300

VL Synechococcus 7335 EHVFGTSQGAGDAILTFLGGFHPQTQSLWLTDMAHHHLAIAVLFIVAGHMYRTNFGIGHS 300

VL Aphanocapsa GSE QQVFGTSQGSGTAILTFLGGFHPQTESLWLTDMAHHHLAIAVLFIVAGHMYRTNFGIGHN 300

VL Chroococcidiopsis thermalis 7203 NHVFGTSQGAGTAILTFLGGFHPQTQSLWLTDIAHHHLAIAVLFIVAGHMYRTNFGIGHS 300

VL Chroococcidiopsis thermalis 7203 NHVFGTSQGAGTAILTFLGGFHPQTQSLWLTDMAHHHLAIAVLFIVAGHMYRTNFGIGHS 300

VL Chroococcidiopsis thermalis 7203 NHVFGTSEGAGTAILTFLGGFHPQTQSLWLTDMAHHHLAIAVIFIIAGHQYRTNFGIGHS 300

VL Pleurocapsa 7327 KHVFGTSEGAGTAILTFLGGFHPQTESLWLTDMAHHHLAIAVIFIIAGHMYRTNWGIGHS 300

Synechocystis 6803 GHIFGTSEGAGTAILTFLGGFHPQTESLWLTDIAHHHLAIAVIFIIAGHMYRTNWGIGHS 300

T. elongatus SHVFGTAQGAGTAILTFLGGFHPQTESLWLTDMAHHHLAIAVLFIVAGHMYRTQFGIGHS 300

: *.:: *:* *************:******:****:****::*:*** ***:: ***.

FRL Ancestral IKEMMDAKQGRKVEGFFIAPRFNLPHQGIYETYNNSLHFQLGWHLACLGVITSLVAQHMY 360

FRL Fischerella thermalis 7521 IKEMMDSKTFF---GRKVEGPFNLPHQGLYETVNNSLHFQLSLALACLGVASSLTAQHMY 357

FRL Halomicronema hongdechloris IKEMTEALQGPGRSGFFIAPRTGRGHKGIYDTYNNSLHFQLGWHLACLGVITSLVAQHMY 360

FRL Synechococcus 7335 IKEMTESLQGPGWTGFFIAPNTGRGHKGIYDAYNNSLHFQLGWHLACLGVVTSLVAQHMY 360

FRL Aphanocapsa GSE IKEMLEALQGPGPKGFFIAPNTGRGHQGIYDTYNESLHFQLGWHLACLGVITSLVAQHMY 360

FRL Chroococcidiopsis thermalis 7203 IKEMLNSKSFF---GAKVEGPFNLPHQGLYDTINNSLHFQLSFALAALGVASSLTAQHMY 357

FRL Pleurocapsa 7327 IKEMLDALQGPGWRGFFIAPRTGRGHLGIFEDYNNSLHFQLGWHLACLGVITSLVAQHMY 360

VL Fischerella thermalis 7521 IKEMMDAKT-F--FGKPVEGPFNMPHQGIYETYNNSLHFQLGWHLACLGVITSLVAQHMY 357

VL Halomicronema hongdechloris MKEIMNAHNPP--QGTPFGGMIGEGHKGMYDTYNNSLHFQLGWHLACLGVITSLVAQHMY 358

VL Synechococcus 7335 IREILNTHRPP--EGTPLGGALGAGHKGLYDTLNNSLHFQLALALASLGVITSLVAQHMY 358

VL Aphanocapsa GSE IKEMLDSKKGL--LSPNSEGQFNLPHQGLYDTMNNSLHFQLAFALAALGVITSLVAQHMY 358

VL Chroococcidiopsis thermalis 7203 IKEMMNAKT-F--FGVPVEGPFNMPHQGIYDTYNNSLHFQLGWHLACLGVITSLVAQHMY 357

VL Chroococcidiopsis thermalis 7203 IKEMLNAKK-F--FGASTEGQFNLPHQGLYDTINNSLHFQLSLALAALGTITSLVAQHMY 357

VL Chroococcidiopsis thermalis 7203 IKEMLNAKN-F--FGIETEGQFNLPHQGLYDTYNNSLHFQLSIHLAALGTALSLVAQHMY 357

VL Pleurocapsa 7327 IKEILNAHNPP--SGTPFGGLLGEGHKGLYDTINNSLHFQLGLALASLGTITSLVAQHMY 358

Synechocystis 6803 IKEILNAHKGP---------LTGAGHTNLYDTINNSLHFQLGLALASLGVITSLVAQHMY 351

T. elongatus IKEMMDAK-DF--FGTKVEGPFNMPHQGIYETYNNSLHFQLGWHLACLGVITSLVAQHMY 357

::*: :: . * .::: *:******. **.**. **.*****

FRL Ancestral SMPPYAFIARDYTTMAALYTHHQYIAGFLMVGAFAHGAIFLVRDYDPEQNKGNVLDRVLQ 420

FRL Fischerella thermalis 7521 SMPPYAFIAKDFTTMAALYTHHQYIAGFLMVGAFSHAAIFWIKDYDPEQNKGNVLERVLK 417

FRL Halomicronema hongdechloris AMPPYAFMARDYTTMSALYTHHQYIAGFLMIGAFAHGAIFLIRDYDPEANRDNVLARMLA 420

FRL Synechococcus 7335 AMPPYAFIARDYTTTAALYTHHQYIAGFLMLGAFAHGGIFLIRDYDPVANENNVLARVLD 420

FRL Aphanocapsa GSE SMPPYAFIAKDFTTMSALYTHHQYIAGFLMVGAFSHGAIFLVRDYDPELNKDNVLARVLA 420

FRL Chroococcidiopsis thermalis 7203 SMPPYAFIGQDFTTQAALYTHHQYIAGFLMVGAFSHAGIFWIRDYDPEQNKGNVLDRMLR 417

FRL Pleurocapsa 7327 SMPPYAFISRDYTAMSALYTHHQYIAGFLMVGAFAHGAIFMVRDYDPELNRDNVLARVLN 420

VL Fischerella thermalis 7521 SLPPYAFIAKDYTTQAALYTHHQYIAIFLMLGAFAHGAIFWVRDYDPEQNKGNVLERVLK 417

VL Halomicronema hongdechloris SLPPYAFLAKSYTTQAALYTHHQYIAGFIMVGAFAHGAIFLIRDYDPASNSNNVLDRVLQ 418

VL Synechococcus 7335 ALPPYAFIAQDFTTQAALYTHHQYIAGFIMMGAFAHGAIFLIRDYDPAANENNVLARVLD 418

VL Aphanocapsa GSE AMPPYAFINQDHTTSAALYTHHQYIAGFIMVGAFAHGAIFLVRDYDPVQNKGNVLDRVLQ 418

VL Chroococcidiopsis thermalis 7203 SLPPYAFMAQDYTTQAALYTHHQYIAGFIMLGAFAHGAIFWVRDYDPEQNKGNVLERVLK 417

VL Chroococcidiopsis thermalis 7203 AMPPYAFIGQDFTTQAALYTHHQYIACALMLGAFAHAAIFWVRDYDPEQNKGNVLDRVLK 417

VL Chroococcidiopsis thermalis 7203 AMPPYAFIGQDFTTQAALYTHHQYIAIAFMIGAFAHAGIFWVRDYDPEQNKGNVLDRVLK 417

VL Pleurocapsa 7327 SMPPYAFLAKDYTTQAALYTHHQYIAGFLITGAFAHGAIFWVRDYDPEANKNNVLARILA 418

Synechocystis 6803 SLPSYAFIAQDHTTQAALYTHHQYIAGFLMVGAFAHGAIFFVRDYDPVANKDNVLARMLE 411

T. elongatus SLPPYAFIAQDHTTMAALYTHHQYIAGFLMVGAFAHGAIFLVRDYDPAQNKGNVLDRVLQ 417

::* ***: :..*: :********** :: ***:*..** ::**** * .*** *:*

| |

FRL Ancestral HKEAIISHLSWVSLFLGFHTLGLYVHNDVEVAFGAPEKQILIEPVFAQFIQAAHGKALYG 480

FRL Fischerella thermalis 7521 HKEAIIAHLSWVSLFLGFHTLGLYVHNDVEVAFGAADKQILIEPVFAQFIQSANGKILYG 477

FRL Halomicronema hongdechloris HKEAIISHLSWVSLFLGFHTLGLYVHNDCEVALGSPEKQILIEPVFAQWTQAFHGKALYG 480

FRL Synechococcus 7335 HKEAIISHLSWVSLFLGFHTLALYVHNDCEVAFGSPDKQILVEPVFAQWIQAVHGKALYG 480

FRL Aphanocapsa GSE HKEAIISHLSWVSMFLGFHTLGLYVHNDVEVAFGAADKQILIEPVFAQFIQSANGKVLYG 480

FRL Chroococcidiopsis thermalis 7203 HKEAIISHLSWVSLFLGFHTLGLYVHNDVEVAFGAAEKQVLIEPVFAQFIQAAHGKALYG 477

FRL Pleurocapsa 7327 HKEAIISHLSWVSLFLGFHTLGIYVHNDCEVAFAAPEKQILIEPVFAQWIQAAHGKTLYG 480

VL Fischerella thermalis 7521 HKEAIISHLSWVSLFLGFHTLGLYVHNDVVVAFGTPEKQILIEPVFAQFIQASHGKVLYG 477

VL Halomicronema hongdechloris HKEAIISHLSWVSLFLGFHTLGLYVHNDVMVAFGTPEKQILVEPVFAQWIQAAHGKLLYG 478

VL Synechococcus 7335 HKEAIISHLSWVSLFLGFHTLGLYVHNDVVVAFGTPEKQILVEPVFAQWVQAASGKALYG 478

VL Aphanocapsa GSE HKEAIISHLSWVSLFLGFHTLGLYVHNDVVVAFGTPEKQILIEPVFAQFIQAAHGKVLYG 478

VL Chroococcidiopsis thermalis 7203 HKEAIISHLSWVSLFLGFHTLSLYVHNDVVVAFGTPEKQILIEPVFAQFIQAAHGKVLYG 477

VL Chroococcidiopsis thermalis 7203 HKEAIISHLSWVSLFLGFHTLGLYVHNDVVVAFGTPEKQILIEPVFAQFIQGAHGKVLYG 477

VL Chroococcidiopsis thermalis 7203 HKEAIISHLSWVSLFLGFHTLGIYVHNDVVVAFGTPEKQILIEPVFAQFIQASHGKVLYG 477

VL Pleurocapsa 7327 HKEAIISHLSWVSLFLGFHTLGLYVHNDVVVAFGTPEKQILIEPVFAQWIQAAHGKALYG 478

Synechocystis 6803 HKEALISHLSWVSLFLGFHTLGLYVHNDVVVAFGTPEKQILIEPVFAQWIQATSGKALYG 471

T. elongatus HKEAIISHLSWVSLFLGFHTLGLYVHNDVVVAFGTPEKQILIEPVFAQFIQAAHGKLLYG 477

****:*:******:*******.:***** **:.: :**:*:******: *. ** ***

|

FRL Ancestral FNTLLSNPDSIASTAWPNYGNVWLPGWLDAINNGTNSLFLTIGPGDFLVHHAIALGLHVT 540

FRL Fischerella thermalis 7521 FHTLLSNPDSIAFTAWPNHANVWLPGWLDAINDGTNSLFLTIGPGDFYVHHAIALGLHVT 537

FRL Halomicronema hongdechloris INSLLSNPDSVASTAWPNYGNVWLSGWLEAVNNGANSLFLTIGPGDLLVHHAIALGLHVT 540

FRL Synechococcus 7335 ISSLLSNPDSIASTAWPNHANVWLPGWLEAINNGTNSLFLAIGPGDFLVHHAIALGLHVT 540

FRL Aphanocapsa GSE MNTLLSNPDSIASTAWPNYGNVWLPGWLEAINNGTNSLFLTIGPGDFLVHHAIALGLHVT 540

FRL Chroococcidiopsis thermalis 7203 FNTLLSNPDSIASTAWPNHANVWLPGWLDAVNNTTNSLFLTIGPGDFYVHHAIALGLHVT 537

FRL Pleurocapsa 7327 LSTLLSNPDSIASTAWPNYGNVWLPGWLDAINSGTNSLFLPIGPGDFLVHHAIALGLHVT 540

VL Fischerella thermalis 7521 LNVLLSNPDSVAYTAYPNYGNVWLSGWLDAINSGTNSLFLTIGPGDFLVHHAFALAIHTT 537

VL Halomicronema hongdechloris FDTLLSNPGSIASTAWPNYGNVWLSGWLDAINSGDNSLFLTIGPGDFLVHHAIALGLHTT 538

VL Synechococcus 7335 FDTLLSNPDSIATTAWPNGGNVWLPGWLDAINSGSNSLFLAIGPGDFLVHHAIALGLHTT 538

VL Aphanocapsa GSE FDTLLSNPDSIAATAWPNYGNVWLPGWLDAINNGANSLFLTIGPGDFLVHHAIALGLHTT 538

VL Chroococcidiopsis thermalis 7203 FDTLLSNPDSIATTAWPNHGNVWLPGWLGAINAGTNSLFLTIGPGDFLVHHAFALALHTT 537

VL Chroococcidiopsis thermalis 7203 FDTLLSNPDSVAST----AGAAWLPNWLDAINNGTNSLFLTIGPGDFLVHHAFALAIHTT 533

VL Chroococcidiopsis thermalis 7203 FNTLLSNPDSIAST----AGATYLPGWYEAINNTTNSLFLTIGPGDFLVHHAFALAIHTT 533

VL Pleurocapsa 7327 FNTLLSNPDSIAYTAYPNYGNVWLPGWLDAINSGTNSLFLTIGPGDFLVHHAIALGLHTT 538

Synechocystis 6803 FDVLLSNPDSIAST----TGAAWLPGWLDAINSGTNSLFLTIGPGDFLVHHAIALGLHTT 527

T. elongatus FDTLLSNPDSIASTAWPNYGNVWLPGWLDAINSGTNSLFLTIGPGDFLVHHAIALGLHTT 537

: *****.*:* * . .:* .* *:* ***** *****: ****:**.:*.*

FRL Ancestral TLILVKGALDARGSKLMPDKKDFGYAFPCDGPGRGGTCDISAWDSFYLAMFWMLNTLGWV 600

FRL Fischerella thermalis 7521 TLILVKGALDARGSKLMPDKKDFGYAFPCDGPGRGGTCDISAWDASYLAVFWMLNTLGWV 597

FRL Halomicronema hongdechloris TLILVKGALDARGSKLMPDKKDFGYSFPCDGPGRGGTCDISAWDAFYLATFWMLNTLGWV 600

FRL Synechococcus 7335 TLILVKGALDARGSKLMPDKKDFGYAFPCDGPGRGGTCDISAWDSVYLATFWMLNTLGWV 600

FRL Aphanocapsa GSE TLILVKGALDARGSRLMPDKKDFGYAFPCDGPGRGGTCDISAWDSSYLSVFWMLNTMGWV 600

FRL Chroococcidiopsis thermalis 7203 TLVLVKGALDARGSKLMPDKKDFGYAFPCDGPGRGGTCDISAWDASYLAVFWMLNTLGWV 597

FRL Pleurocapsa 7327 TLILVKGALDARGSKLMPDKKDFGYAFPCDGPGRGGTCDISAWDAFYLAMFWMLNTLGWL 600

VL Fischerella thermalis 7521 TLVLVKGALDARGSKLMPDKKDFGYAFPCDGPGRGGTCDISAWDAFYLATFWALNTVGWV 597

VL Halomicronema hongdechloris TLILVKGALDARGSKLMPDKKDFGYSFPCDGPGRGGTCDISAWDAFYLAVFWMLNTIGWV 598

VL Synechococcus 7335 TLILVKGALDGRGSKLMPDKKDFGYSFPCDGPGRGGTCDISGWDSFYLAMFWMLNTIGWV 598

VL Aphanocapsa GSE TLILVKGALDARGSKLMPDKKDFGYAFPCDGPGRGGTCDISAWDSFYLAMFWMLNTIGWI 598

VL Chroococcidiopsis thermalis 7203 TLVLVKGALDARGSKLMPDKKDFGYAFPCDGPGRGGTCDISAWDSFYLAAFWMLNTIGWV 597

VL Chroococcidiopsis thermalis 7203 VLVLVKGALDARGSKLMPDKKDFGYAFPCDGPGRGGTCDISAWDSFYLAAFWVLNTAGWV 593

VL Chroococcidiopsis thermalis 7203 VLVLVKGALDARGSKLMPDKKDFGYAFPCDGPGRGGTCDISAWDSFYLAAFWGLNTAGWV 593

VL Pleurocapsa 7327 TLILVKGALDARGSKLMPDKKDFGYSFPCDGPGRGGTCDISAWDAFYLALFWTLNTLGWL 598

Synechocystis 6803 ALILIKGALDARGSKLMPDKKDFGYSFPCDGPGRGGTCDISAWDAFYLAMFWMLNTLGWL 587

T. elongatus TLILVKGALDARGSKLMPDKKDFGYAFPCDGPGRGGTCDISAWDAFYLAMFWMLNTIGWV 597

.*:*:*****.***:**********:***************.**: **: ** *** **:

FRL Ancestral TFYWHWKHLAIWQGNVAQFNESSTYLMGWFRDYLWLNSAQLINGYNPYGTNNLAVWAWMF 660

FRL Fischerella thermalis 7521 TFYWHWKHLSIWQGNVAQFNESSTYLMGWFRDYLWANSAQLINGYNPYGTSNLAVWAWMF 657

FRL Halomicronema hongdechloris TFYWHWKHLSVWSGNVAQFNESSTYLMGWFRDYLWANSAQLINGYSPAGTNSLAVWAWMF 660

FRL Synechococcus 7335 TFYWHWKHLAIWSGNVAQFNEGSTYLMGWFRDYLWLNSAQLINGYNPYGTNNLAIWAWIF 660

FRL Aphanocapsa GSE TFYWHWKHLAIWSGTLAQFNESSTYLMGWFRDYLWLYSGPLVNGYNPFGTSNLAVWAWMF 660

FRL Chroococcidiopsis thermalis 7203 TFYWHWKHLAIWEGNIAQFNESSTYLMGWFRDYLWLHSAQLINGYNPYGTNSLAIWSWMF 657

FRL Pleurocapsa 7327 TFYWHWKHLTLWSGNVAIFNENSTYLMGWFRDYLWANSAQLINGYNPAGTNNLAVWAWMF 660

VL Fischerella thermalis 7521 TFYWHWKHLGIWQGNVAQFNESSTYLMGWFRDYLWANSAQLINGYNPYGMNNLSVWAWMF 657

VL Halomicronema hongdechloris TFYWHWKHLAIWQGNVAQFNESSTYLMGWLRDYLWLNSSQLINGYNPYGMNNLAVWAWMF 658

VL Synechococcus 7335 TFYWHWKHLAIWSGNVAQFNESSNYLMGWLRDYLWLNSSQLINGYNPYGMNNLAVWAWMF 658

VL Aphanocapsa GSE TFYWHWKHLGIWEGNVAIFNESSTYLMGWFKDYLWLNSSQLINGYNPYGTNNLSVWAWMF 658

VL Chroococcidiopsis thermalis 7203 TFYWHWKHLGIWQGNVAQFNESSTYLMGWLRDYLWLYSAQLINGYNPYGMNNLSVWSWMF 657

VL Chroococcidiopsis thermalis 7203 TFYWHWKHLGIWQGNVAQFNESSTYLMGWLRDYLWLYSAQLINGYNPYGMNNLSVWAWMF 653

VL Chroococcidiopsis thermalis 7203 TFYWHWKHLGIWQGNVAQFNESSTYLMGWLRDYLWLYSAQLINGYNPYGMNNLSVWAWMF 653

VL Pleurocapsa 7327 TFYWHWKHLCVWQGNVAQFNENSTYLMGWFRDYLWANSAQLINGYNPYGVNNLSVWAWMF 658

Synechocystis 6803 TFYWHWKHLGVWSGNVAQFNENSTYLMGWFRDYLWANSAQLINGYNPYGVNNLSVWAWMF 647

T. elongatus TFYWHWKHLGVWEGNVAQFNESSTYLMGWLRDYLWLNSSQLINGYNPFGTNNLSVWAWMF 657

********* :*.*.:* ***.*.*****::**** *. *:***.* * ..*::*:*:*

|| | | | |||

FRL Ancestral LFGHLAWAVSFMFLITWRGYWQELIETLMWAHENTPLS-FGYPKDKPVALSIVQARLVGL 719

FRL Fischerella thermalis 7521 LFGHLAWAVSFMFLITWRGYWQELIETLAWAHEQTPLS-FGYWRDKPVALSIVQARLVGL 716

FRL Halomicronema hongdechloris LFGHLAWAVSFMFLITWRGYWQELIETLMWAHENTPLS-FGYPKDKPVALSIVQARLVGL 719

FRL Synechococcus 7335 LFGHLVWAISFMFLITWRGYWQELIETLMWAHENTPLS-FGYPKDKPVALSIVQARLVGL 719

FRL Aphanocapsa GSE LFGHLAWAVSFMFLITWRGYWQELIETLLWAHENTPLS-FGYPKDKPVALSIVQARLVGL 719

FRL Chroococcidiopsis thermalis 7203 LWGHLAWAVSFMFLITWRGYWQELIETLVWAHEKTPLS-FGYWRDKPVALSIVQARLVGL 716

FRL Pleurocapsa 7327 LFGHLAWAVSFMFLITWRGYWQELIETLMWAHENTPLS-FGYPKDKPVALSIVQARLVGL 719

VL Fischerella thermalis 7521 LFGHLVWATGFMFLISWRGYWQELIETLVWAHERTPIANLVRWKDKPVALSIVQARLVGL 717

VL Halomicronema hongdechloris LLGHLVWATGFMFLISWRGYWQELIETLVWAHERTPLANLVRWKDKPVALSIVQARLVGL 718

VL Synechococcus 7335 LFGHLVWATGFMFLISWRGYWQELIETIVWAHERTPLANLVRWKDKPVAMSIVQGRLIGL 718

VL Aphanocapsa GSE LFGHLVWATGFMFLISWRGYWQELIETLVWAHERTPLANLVRWKDKPVALSIVQARVVGL 718

VL Chroococcidiopsis thermalis 7203 LLGHLVWATGFMFLISWRGYWQELIETLVWAHERTPLANLIRWKDKPVALSIVQARVVGL 717

VL Chroococcidiopsis thermalis 7203 LLGHLIWATGFMFLISWRGYWQELIETLVWAHERTPLANLIRWKDKPVALSIVQARIVGL 713

VL Chroococcidiopsis thermalis 7203 LFGHLVWATGFMFLISWRGYWQELIETLVWAHERTPLANLIRWKDKPVAMSIVQGRLVGL 713

VL Pleurocapsa 7327 LFGHLVWATGFMFLISWRGYWQELIETIVWAHERTPLANLVRWKDKPVALSIVQARLVGL 718

Synechocystis 6803 LFGHLVWATGFMFLISWRGYWQELIETIVWAHERTPLANLVRWKDKPVALSIVQARLVGL 707

T. elongatus LFGHLVWATGFMFLISWRGYWQELIETLVWAHERTPLANLVRWKDKPVALSIVQARLVGL 717

* *** ** .*****:***********: ****.**:: : :*****:****.*::**

|

FRL Ancestral THFTVGYIATYGAFLIASTASKFG- 743

FRL Fischerella thermalis 7521 THFTVGYIATYGAFLIASTASKFGQ 741

FRL Halomicronema hongdechloris THFTVGYIATYGAFLIASTSSRFP- 743

FRL Synechococcus 7335 VHFTVGYIATYGAFLIASTGSRFP- 743

FRL Aphanocapsa GSE THFTVGYIATYGAFLIGATASLYG- 743

FRL Chroococcidiopsis thermalis 7203 THFTVGYIATYGAFLIASTAGKFG- 740

FRL Pleurocapsa 7327 THFTVGYIATYGAFLIASTASRFG- 743

VL Fischerella thermalis 7521 VHFSVGYVLTYAAFLIASTAGKFG- 741

VL Halomicronema hongdechloris AHFSVGYIITYAAFLIASTSSRFG- 742

VL Synechococcus 7335 AHFTVGYVLTYAAFLIASTSSRFG- 742

VL Aphanocapsa GSE AHFTVGYVLTYAAFLIASTASRYG- 742

VL Chroococcidiopsis thermalis 7203 AHFAVGYVLTYAAFVIASTAGKFG- 741

VL Chroococcidiopsis thermalis 7203 GHFAAGYILTYAAFLIASTAGKFG- 737

VL Chroococcidiopsis thermalis 7203 VHFAVGYILTYGAFLIASTAGKFG- 737

VL Pleurocapsa 7327 AHFTVGYVLTYAAFLIASTAGKFG- 742

Synechocystis 6803 AHFTVGYVLTYAAFLIASTAGKFG- 731

T. elongatus AHFSVGYILTYAAFLIASTAAKFG- 741

**:.**: **.**:*.:*.. :

PsaF

||| | |

FRL Ancestral MRKMMRRLFAQLFAG-VLWFSILPPAAAEATSSGNTTLVPCSESPAFQERMKNAPDSLGD 59

FRL Fischerella thermalis 7521 ----MKRIFALILAI-FIWFSAVSTAL-----AENTTLVPCYKSPAFVERMKNAPDS--- 47

FRL Halomicronema hongdechloris ----MRALFAIVIMS-FVWFNAIPPAIAA----GDTHLVPCQESTAFMENLQNAPKS--- 48

FRL Synechococcus 7335 MHKTIRKFFSLLLAA-FVWLSVVSPAVAASEGYTDTHLVPCASSPAFNERMQNAPEG--- 56

FRL Aphanocapsa GSE ----MKRWFGLILAI-ALWFSLVPNAL-----AANTTLVPCKDSSAFLNRQKKAPDS--- 47

FRL Chroococcidiopsis thermalis 7203 ---MRRLLFTLAIAF-VIWGSTIPTAS-----AANSTLVPCSKSPAFQARMKNAPDT--- 48

FRL Pleurocapsa 7327 ----MKRLLALVLIL-AIWFTLVPAAW-----AQTSHLVPCKDSPAYQERMKKAPDN--- 47

VL Fischerella thermalis 7521 ----MRRLFALILAI-CLWFNFAPAANAL-----GADLVPCSESSAFAQRAQVARNTTAD 50

VL Halomicronema hongdechloris ----MRRLFALVLGI-ISWFGIALPAA-----ADVAGLTPCSESAAFQQRAANAATEA-- 48

VL Synechococcus 7335 ----MRRLFALALVL-CLSLGFAAPATAGIAGDDVAGLVPCNESAAFQKRAAAAPTDE-- 53

VL Aphanocapsa GSE ----MRRLFALVLVC-LLWVGFAPSASAD-----VAGLVPCKDSPAFQKRAAAARNTTDD 50

VL Chroococcidiopsis thermalis 7203 ----MRRLFALILVIGGLWFTFAPPAQAQ-----QVNLVRCSDSPAFVQRAQAARNTTAD 51

VL Pleurocapsa 7327 ----MKHLLALILIV-TLWFNFAPPASAD---GSVAGLTPCSETPAFKQKSKNFLNTTAD 52

Synechocystis 6803 ----MKHLLALLLAF-TLWFNFAPSASAD----DFANLTPCSENPAYLAKSKNFLNTTND 51

T. elongatus ----MRRFLALLLVL-TLWLGFTPLASAD-----VAGLVPCKDSPAFQKRAAAAVNTTAD 50

: : : * *. * .. *: .

| | | | || | |

FRL Ancestral PLYYTKPFKAYSQSNLLCGEDGLPHLPLDGRRSRAVDVVIPIALFLYIAGWIGWSGRSYL 119

FRL Fischerella thermalis 7521 -YYTTKPLKAYS--QLLCGEDGLPRIALD-RLSLAVDVAIPIAIFLYTAGFIGWSGRSYL 103

FRL Halomicronema hongdechloris -YYFDQPYQAYSK-NLLCGEDGLPHLQLR--LDRAVDIAIPFVIFFYFAGFVGWSGRAYL 104

FRL Synechococcus 7335 -YYFDTPYQSYAA-NLLCGAEGLPHQQLR--FDRAIDVLIPFGIFFYVAGFIGWSGRAYL 112

FRL Aphanocapsa GSE -YYFRQPYQAYSE-YLVCGEDGLPHLPLD-RLDRAVDVAIPIGLFLYIAGFIGWSGRSYL 104

FRL Chroococcidiopsis thermalis 7203 -YYFNKPFKAYAK-YELCGTDGLPHLPLD-RLDRATDVLVPIGLFLYVAGFIGWSGRSYL 105

FRL Pleurocapsa 7327 -YYFNKPGKAYAE-YLLCGEDGLPHLALS--FDRAGDIAIAFGIFFYFTGFVGWSGRSYL 103

VL Fischerella thermalis 7521 PQSGQKRFERY--SQAYCGPEGLPHLIVDGRLDRAGDFLIPSILFLYIAGWIGWVGRAYL 108

VL Halomicronema hongdechloris ---AKARFDFYGSSNLLCGDDGLPHLIVDGDLSHVGEFLIPSILFLYIAGWIGWAGRSYL 105

VL Synechococcus 7335 ---AKARFEFYGNTSLLCGPEGLPHLVVDGDLAHAGEFLIPSLLFLLIAGWIGWAGRSYV 110

VL Aphanocapsa GSE PASGQKRFERY--SQALCGPEGLPHLIIDGRRGREGDFLIPGVLFLYITGWIGWVGRAYI 108

VL Chroococcidiopsis thermalis 7203 PQSGEKRFERY--AQAMCGPEGLPHLVVDGRLDRAGDFLIPSIMFLYIAGQIGWAGRAYL 109

VL Pleurocapsa 7327 PKSGQKRAERY--AQALCGPEGYPHLIVDGRWDHMGDFFIPSILFLYIAGQIGWAGRAYL 110

Synechocystis 6803 PNSGKIRAERY--ASALCGPEGYPHLIVDGRFTHAGDFLIPSILFLYIAGWIGWVGRSYL 109

T. elongatus PASGQKRFERY--SQALCGEDGLPHLVVDGRLSRAGDFLIPSVLFLYIAGWIGWVGRAYL 108

. * ** :* *: : :. : :*: :* :** **:*:

| |

FRL Ancestral IAIRKS-GKNPEEKEIFIDVPLAIQCMAKGLLWPLLALKEFLSGEITAKDEEIPISPR 176

FRL Fischerella thermalis 7521 QAIKKQ-DK-AEEKEVFIDVPLFISCMVMALFWPMAVIKELLAGELVAKDEEIPISVR 159

FRL Halomicronema hongdechloris INSKK--ASKPEEMEIFINVPLAIQSFIQGLLWPLAAFRELTAKELTAKDSELSVSPR 160

FRL Synechococcus 7335 ISSNR--NSKPEETEIFIDVALAIKSFVQGLLWPLLAVKELTTGELTAPVSEVSVSPR 168

FRL Aphanocapsa GSE IAARK--TSNPEEKEIFIDIGLAIQSLAKGLFWPVAALNELISGKLTSKEKDIYVSPR 160

FRL Chroococcidiopsis thermalis 7203 RATKS--ASDPEMKEIFIDLPLALQSISKAVLWPLLALQEFLSGDLTARDEEIPISPR 161

FRL Pleurocapsa 7327 QVSNQ--SKNPEQMEIFIDIPLAIQSFAKGLLWPVLAFQELVTGQLTAKDSEIPVSPR 159

VL Fischerella thermalis 7521 QTIKKQ-GGDVEQKEIQIDVPLALPIMLSGFAWPAAAIKELLSGELTAKDEEIPISPR 165

VL Halomicronema hongdechloris IAVRS--EKKPEEKEIIIDVPLALKCSLSGFAWPLTAFRDIASGEMFAKDTEIPVSPR 161

VL Synechococcus 7335 IAVRS--EKSPEEKEIVIDVPLAIKCSLSGATWPLLAFKEITSGEMFAKKEEITVSPR 166

VL Aphanocapsa GSE IAARK--SDNPAQKEIVIDVPLAIKCVAQGPLWPLLALKELTSGELLAKDGDVTVAPR 164

VL Chroococcidiopsis thermalis 7203 QATKKKYASETEIKEVIIDVPLAFQSMVSAFAWPLAAVKELLSGELTAKDEEIPISPR 167

VL Pleurocapsa 7327 IAIRD--EKDAEMKEIIIDVPLALKQMIAAFAWPLLAVKEFLAGTLTAKDSEIPVSPR 166

Synechocystis 6803 IEIRE--SKNPEMQEVVINVPLAIKKMLGGFLWPLAAVGEYTSGKLVMKDSEIPTSPR 165

T. elongatus IAVRN--SGEANEKEIIIDVPLAIKCMLTGFAWPLAALKELASGELTAKDNEITVSPR 164

. *: *:: * : . ** .. : : : :: : *

PsaI

| || |

FRL Ancestral -MADLTQMTGSYATSYLSWIFIPLITYILPFPVFALLFLWIEREPGLEKEVESSQQIINR 59

FRL Fischerella thermalis 7521 MMVDMTQLTGDYAASWLPWIMIPLVFYILPFPVFAILFLWIQKEASEEIKETDNNLA-E- 58

FRL Halomicronema hongdechloris -MADMTQLTGAYAAPWLPWIMIPLIFYILPFPIFAIIFLWIEREGNGVNDMGGEPMK-S- 57

FRL Synechococcus 7335 -MVDATQLEGAYAAAWLPWIMIPMITYILPFPIFAIAFLWIEREGGEGGLDIDVMGS-N- 57

FRL Aphanocapsa GSE -MVDLTQLTGDYAASWLPWIMIPLIFYILPFPVVAIVFLWIEQETTEEEV---------- 49

FRL Chroococcidiopsis thermalis 7203 -MVDMTQLTGSYAASWLPWIMIPLIFYILPFPVFALIFIWIEKEAGTADEEV-------- 51

FRL Pleurocapsa 7327 -MVDMTQLTGSYAASWLPWIMIPLVFYILPFPVFALLFLWIEKEGSQEESPREQFQN-S- 57

VL Fischerella thermalis 7521 ----MASLLASYPASFLSPILVYSIGWIVPIVVFSFMLIYIEREDIA------------- 43

VL Halomicronema hongdechloris -------MDGTYAASWLPWLLIPVVTWLMPAVVMGLLFFYIESDA--------------- 38

VL Synechococcus 7335 -----------MSASFLPTILVPTVGLVFPAIAMAALFLYIERGQATTGGESAPWGQVSE 49

VL Aphanocapsa GSE -------MTGSYAASFLPWILIPIVCWLMPVVVMGLLFIHIESDAA-------------- 39

VL Chroococcidiopsis thermalis 7203 ----------MFSASFLPSILVPLTVLVFPSVAMALLFLYIEREDPSGI----------- 39

VL Pleurocapsa 7327 -------MTGTYAASYLPWILIPIVCWLLPAVVMGLLFIYIESEA--------------- 38

Synechocystis 6803 -------MDGSYAASYLPWILIPMVGWLFPAVTMGLLFIHIESEGEG------------- 40

T. elongatus -------MMGSYAASFLPWIFIPVVCWLMPTVVMGLLFLYIEGEA--------------- 38

: :* ::: :.* .. :: *:

FRL Ancestral QTKQALEAAPVIEDLDLSTS-- 79

FRL Fischerella thermalis 7521 -----IGELEVPNS-------- 67

FRL Halomicronema hongdechloris -----DGNYPV----------- 63

FRL Synechococcus 7335 -----AMS---NEAMGRDISS- 70

FRL Aphanocapsa GSE ---------------------- 49

FRL Chroococcidiopsis thermalis 7203 ---------------------- 51

FRL Pleurocapsa 7327 -----VERVNVIEQFGVTEQQG 74

VL Fischerella thermalis 7521 ---------------------- 43

VL Halomicronema hongdechloris ---------------------- 38

VL Synechococcus 7335 DSQTDVV--------------- 56

VL Aphanocapsa GSE ---------------------- 39

VL Chroococcidiopsis thermalis 7203 ---------------------- 39

VL Pleurocapsa 7327 ---------------------- 38

Synechocystis 6803 ---------------------- 40

T. elongatus ---------------------- 38

PsaJ

| | | |||||

FRL Ancestral -------MQMQYFLKYLTSAPVMATLTMVILAGVLIELNRFFPGLQYGTYFHALQVP 50

FRL Fischerella thermalis 7521 -------MEARYLFRYLSSAPVVATLALIIISVILIVLNYLFPGLQYGTFFHSLP-- 48

FRL Halomicronema hongdechloris ---------MSYFVKYLTSAPVMATLALVILSVVMIELNHIFPGLQYGTYFHVAP-- 46

FRL Synechococcus 7335 ---------MKYFAKYLTSAPIMATVALVSLSVVLIELNHFFPGLQYGTYFHSVP-- 46

FRL Aphanocapsa GSE ---------MSYFGKYLTSAPVISLITLVAISVFLIALNYYYPGLQYGTYFKPLP-- 46

FRL Chroococcidiopsis thermalis 7203 ---------MQHVFKYLTLAPVMATFTMVALSVVLIMLQIWFPGLQYGTYFKPTP-- 46

FRL Pleurocapsa 7327 ---------MQSFFKYLTLAPVMAILSLVILFVVFIELNYFYPGLQYGTYFHSLP-- 46

VL Fischerella thermalis 7521 ------MDNQSPFFKFLSTAPVITTIWLFITAGILIEFNRFFPDL----LFHPLP-- 45

VL Halomicronema hongdechloris ---------MDNLVKYLSTAPVVAAIWMAITAGVLIEFNRFFPDL----LFHP---- 40

VL Synechococcus 7335 --------MSSNLLKYLSTAPVIATVWMVITAGILIEFNRFFPDL----LLHP---- 41

VL Aphanocapsa GSE ---------MKYFVTFLSTAPVITAIWLTITAGILIEFNRFYPDL----LFHPL--- 41

VL Chroococcidiopsis thermalis 7203 MQKQNENEQKHYLQRYLSLAPVLAVVAVSVAFTTWAIFNYFFPDL----LFHPMP-- 51

VL Chroococcidiopsis thermalis 7203 -------MQQKYFLQYLSLAPVLLFAWLAETAVWLIVFNYFFPDL----LFHPLP-- 44

VL Pleurocapsa 7327 ------------MAKFLSLAPVILFVWLAETAVWLIVFNNIYPDL----LFHPLG-- 39

Synechocystis 6803 ---------MDGLKSFLSTAPVMIMALLTFTAGILIEFNRFYPDL----LFHP---- 40

T. elongatus ---------MKHFLTYLSTAPVLAAIWMTITAGILIEFNRFYPDL----LFHPL--- 41

. :*: **:: : :: :*.* ::

PsaL

FRL Ancestral ------------SAKMSNTTDTDIIKPYKGDPFLGNLSTPINDSPLARAFINNLPAYRKG 48

FRL Fischerella thermalis 7521 ------------MSNTVDTVDNDIIKPFKGDPCLGNLSTPINDSPLAKAFINNLPAYRKG 48

FRL Halomicronema hongdechloris ---MTNTET---STWVDAYDQKDIIQPYRGNPELGNLATPVNSSNLVKTYINNLPAYRPG 54

FRL Synechococcus 7335 ------------MSASDAYISDDPIQPYQGNPQLGNLATPINSSNLAKAFINNLPAYRPG 48

FRL Aphanocapsa GSE -----------------------MVQPYKNDPFRGNLSTPINDSSLAKAFIRNLPAYRPG 37

FRL Chroococcidiopsis thermalis 7203 ---MTNTANPSVESKLNYRLDTDPVQPYKGDPFNSNFSTAITDSPLARAFINNLPAYRKG 57

FRL Pleurocapsa 7327 ---MTTTET---QSLPQAYSELDPIKPYQNDPWKGNLSTPFNDSPIVRAYIRNLPAYRPG 54

VL Fischerella thermalis 7521 ---MAQAV-DASKNLPSDPRNREVVFPATRDPQIGNLETPINSSALTKWFINNLPAYRPG 56

VL Halomicronema hongdechloris -------------------MTNQVVKPYLDEPELGHLSTPISDSAFVRSFIGNLPAYRKG 41

VL Halomicronema hongdechloris --------------------MTDSI-RPAGDPQIGNLATPINSSSVTKTFIGNLPAYRPG 39

VL Synechococcus 7335 -----------------MPASSNFIKPYEGDPQIGNLETPLNSSGLSKAFLENLPAYRTG 43

VL Aphanocapsa GSE MATFVEKI-EQSNDNPRDPRNREVVFAA-DSPFNGNLATPINSSPLAKAFINSLPAYRPG 58

VL Chroococcidiopsis thermalis 7203 ---MAQAI-DASKNRPGDPRNQEVVFPAGRDPQNSNLETPVNSSGLVKWFINNLPAYRPG 56

VL Pleurocapsa 7327 -------------------------MKLAGSPYTGNLSTPVNSSDFTLFLLKNLPIYRAG 35

VL Pleurocapsa 7327 -----------------MADYKQVVQPYNGDPFTGHLATPISASDFTKAFIGNLPAYRPG 43

Synechocystis 6803 -----------------MAESNQVVQAYNGDPFVGHLSTPISDSAFTRTFIGNLPAYRKG 43

T. elongatus -------------------MAEELVKPYNGDPFVGHLSTPISDSGLVKTFIGNLPAYRQG 41

.* .:: * .. * . : .** ** *

| | |||| | | | | | |

FRL Ancestral LTPFMRGLEIGMAHGYFLVGPEVVVGPLRESAHGANLSGLITAIYIAVSACLGISIFAIT 108

FRL Fischerella thermalis 7521 LTPFMRGLEIGMAHGYFLVGPEVVIGPLRESAHGANLSGLITAIYIAVSACLGISIFAIT 108

FRL Halomicronema hongdechloris LTPFLRGLEIGMAHGYFLVGPEVVVGPLRETAHGANLSGLITAIYITVSACLGISIFALA 114

FRL Synechococcus 7335 LTPFLRGLEIGMAHGYFLVGPEVVFGPLKEGSHGANLSGLITAIYITVSACLGISIFALA 108

FRL Aphanocapsa GSE LSPFMRGLEIGMAHGYFLVGPEIVVGPLRETAHGANLSGLITAIYITASACLGISLFAMT 97

FRL Chroococcidiopsis thermalis 7203 LTPFMRGLEIGMAHGYFLVGPEVVVGPLRETAHGANLSGLITAIYIAVSACLGISIFAIT 117

FRL Pleurocapsa 7327 LTPFMRGLEIGMAHGYFLVGPQVVVGPLRETAHGANLSGLICAIYIAVSAVLGISIFALA 114

VL Fischerella thermalis 7521 ITPFRRGLEVGMAHGYWIFGPFAKLGPLRNTV-NADLAGLLSTIGLLVILTIALSLYANS 115

VL Halomicronema hongdechloris MAPITRGLEIGLAHGYFLVGPEIIVGALRDYAPAPYLGGLVTAIAIVLLGTTGMGAHGLV 101

VL Halomicronema hongdechloris LSPQRRGLEIGMAHGYLLYGPFALLGPLRDSD-IPGLAGLLGAAALVVILTACLSIYSGA 98

VL Synechococcus 7335 LSAQRRGLEVGMAHGYLLYGPFALLGPLRDTD-VLGITGLLSAIGLVLILTVCLSIYGGA 102

VL Aphanocapsa GSE LTPLRRGLEVGIVHGYWLVGPFFKFNPFRFTD-YGSLVALISTISLILISTLAISLYAGS 117

VL Chroococcidiopsis thermalis 7203 ITDMRRGLEVGMAHGYWVLGPFTKLGPLRDTD-VANIAGLISTLGMVAIMTATMALYSAS 115

VL Pleurocapsa 7327 LSTLARGLEIGMAHGYFILGPFVKLGPLRDTE-QANLIGLISACTLIVIMTICLSIYGTV 94

VL Pleurocapsa 7327 LSPLLRGLEIGMAHGYFIIGPWVLLGPLRDSE-FASLGGLISGLAIILIATACLASYGLV 102

Synechocystis 6803 LSPILRGLEVGMAHGYFLIGPWTLLGPLRDSE-YQYIGGLIGALALILVATAALSSYGLV 102

T. elongatus LSPILRGLEVGMAHGYFLIGPWVKLGPLRDSD-VANLGGLISGIALILVATACLAAYGLV 100

:: ****:*:.*** : ** .. :: : .*: : :. ..

|| | | |||| | |

FRL Ancestral TFQGNPRG---AYNSYSKDQLRPLRTREEWSQLNGGIFLGAMGGAIFAYFLLENFDALDA 165

FRL Fischerella thermalis 7521 TFQGNPKG---SYSSYSKDSLRPLRTREEWSQLNGGIFLGAMGGAIFAYLLLENFDALDA 165

FRL Halomicronema hongdechloris TFQGDPRG---AYNSNSPDRLRPLRSKDGWFQLSGGILLGSMGGAIFAYVLLENFGDLDA 171

FRL Synechococcus 7335 TFQGDPRG---TYNSHSRDRLRPLRKKEDWYQLSGGILMGSLGGAIFAYALLENFELLDS 165

FRL Aphanocapsa GSE TFQGNPKG---AYNSNCKDELRPLKAKEEWFQLNGGVFLGAMGGAIFAYLLLENFVDLDA 154

FRL Chroococcidiopsis thermalis 7203 TFQGDPRG---AYGSTSKDSLRPLRNRDEWYQLNGGIFLGAMGGAVFAYLLLENFDALDS 174

FRL Pleurocapsa 7327 MFQNNPKG---SYNSYSKDDLRPLRSKEDWFQLAGGVFLGSMGGAIFAYLLLENFDDLDA 171

VL Fischerella thermalis 7521 NPPEPV-----ASVTA-PHPSDAFHTKEGWSNFGSAFLIGGIGGAVTAYFLTANFGLIQG 169

VL Halomicronema hongdechloris SLKPVAES---------SPKTDALMTSEGWSEMTAGFFLGGMSGAFMAYFLLSHFSEIDA 152

VL Halomicronema hongdechloris GVNKGV-----SEATTPFDPPEALSHDEGWSEFAGGFLIGGIGGATFAYLLAANMPLLLS 153

VL Synechococcus 7335 DVSSEI-----SRNTLPYQPPEALSTDEGWSEFAGSFLIGGIGGAIFAYFLSANLPLLLG 157

VL Aphanocapsa GSE NPPPPA-----ATITT-PNPPIALANAEGWNKYATGFLIGGVGGAIVAFAIVANIDVFGN 171

VL Chroococcidiopsis thermalis 7203 NPPQPV-----ATTTTGGQVPSTFKSPESWNNYISGFLIGGVGGAVFAYFVLTNIAIIKN 170

VL Pleurocapsa 7327 SFKKELSKDRLTYSTSVPNVPDSLKTVDGWSQFSGAFLVGGVGGAIFAYLLLDNLGVLQT 154

VL Pleurocapsa 7327 SFQGGRAI---------GTGDASLKSSEGWSQFTAGFFVGGMGGAFTAYFLLENLGVIDA 153

Synechocystis 6803 TFQGEQGS------------GDTLQTADGWSQFAAGFFVGGMGGAFVAYFLLENLSVVDG 150

T. elongatus SFQKGGSS------------SDPLKTSEGWSQFTAGFFVGAMGSAFVAFFLLENFSVVDG 148

: : * : ..::*.:..* *: : :: .

|

FRL Ancestral ILRGAVNVS--------- 174

FRL Fischerella thermalis 7521 ILRGAVNAS--------- 174

FRL Halomicronema hongdechloris ILRGAVNVSQWLGGGVMG 189

FRL Synechococcus 7335 ILRGAVNVG--------- 174

FRL Aphanocapsa GSE ILRGAVNIN--------- 163

FRL Chroococcidiopsis thermalis 7203 ILRGGVNVN--------- 183

FRL Pleurocapsa 7327 ILRGAVNVSQQWLPWVTG 189

VL Fischerella thermalis 7521 FFG--------------- 172

VL Halomicronema hongdechloris IFRGFVN----------- 159

VL Halomicronema hongdechloris VVAGGHS----------- 160

VL Synechococcus 7335 SIAGA------------- 162

VL Aphanocapsa GSE F-RNLFGL---------- 178

VL Chroococcidiopsis thermalis 7203 VFGGLFS----------- 177

VL Pleurocapsa 7327 IATGKI------------ 160

VL Pleurocapsa 7327 IMRGLVNN---------- 161

Synechocystis 6803 IFRGLFN----------- 157

T. elongatus IMTGLFN----------- 155

**Supplementary Figure 6.** Multiple sequence alignment of the calculated ancestral FRL subunit sequences, PSI subunits that are FRL-specific, PSI subunits that are VL-specific, and PSI subunits that are from species that do not perform FaRLiP. Positions assigned as FRL-specific are highlighted in yellow and correspond to the spheres in **Fig. 3**. The top sequence in purple font is the calculated ancestral sequence (see Materials and Methods). Sequences with red font corresponds to FRL-specific sequences from extant cyanobacteria. Positions are labeled with a vertical line if they are FRL-specific, have an ancestral residue consistent with the FRL-specific residues, and have a posterior probability >90% (these criteria suggest that the residue was present in the ancestral FRL sequence). The organism names correspond to *Fischerella thermalis* PCC 7521 (*Fischerella thermalis* 7521), *Halomicronema hongdechloris*, *Synechococcus* sp. PCC 7335 (*Synechococcus* 7335), *Aphanocapsa* sp. GSE-SYN-MK-11-07L (*Aphanocapsa* GSE), *Chroococcidiopsis thermalis* 7203 (*Chroococcidiopsis thermalis* PCC 7203), *Pleurocapsa* sp. PCC 7327 (*Pleurocapsa* 7327), *Synechocystis* sp. PCC 6803 (*Synechocystis* 6803), and *Thermosynechococcus elongatus* (*T. elongatus*). There are FRL-PSI structures for *Fischerella thermalis* 7521 (PDB 7LX0), *Halomicronema hongdechloris* (PDB 6KMX, although the Chl *f*-containing sites were reassigned in Gisriel et al., 2021), and *Synechococcus* 7335 (PDB 7S3D). There is a VL-PSI structure from *Halomicronema hongdechloris* (PDB 6KMW). Clustal Omega sequence conservation identifiers are shown below the alignment.


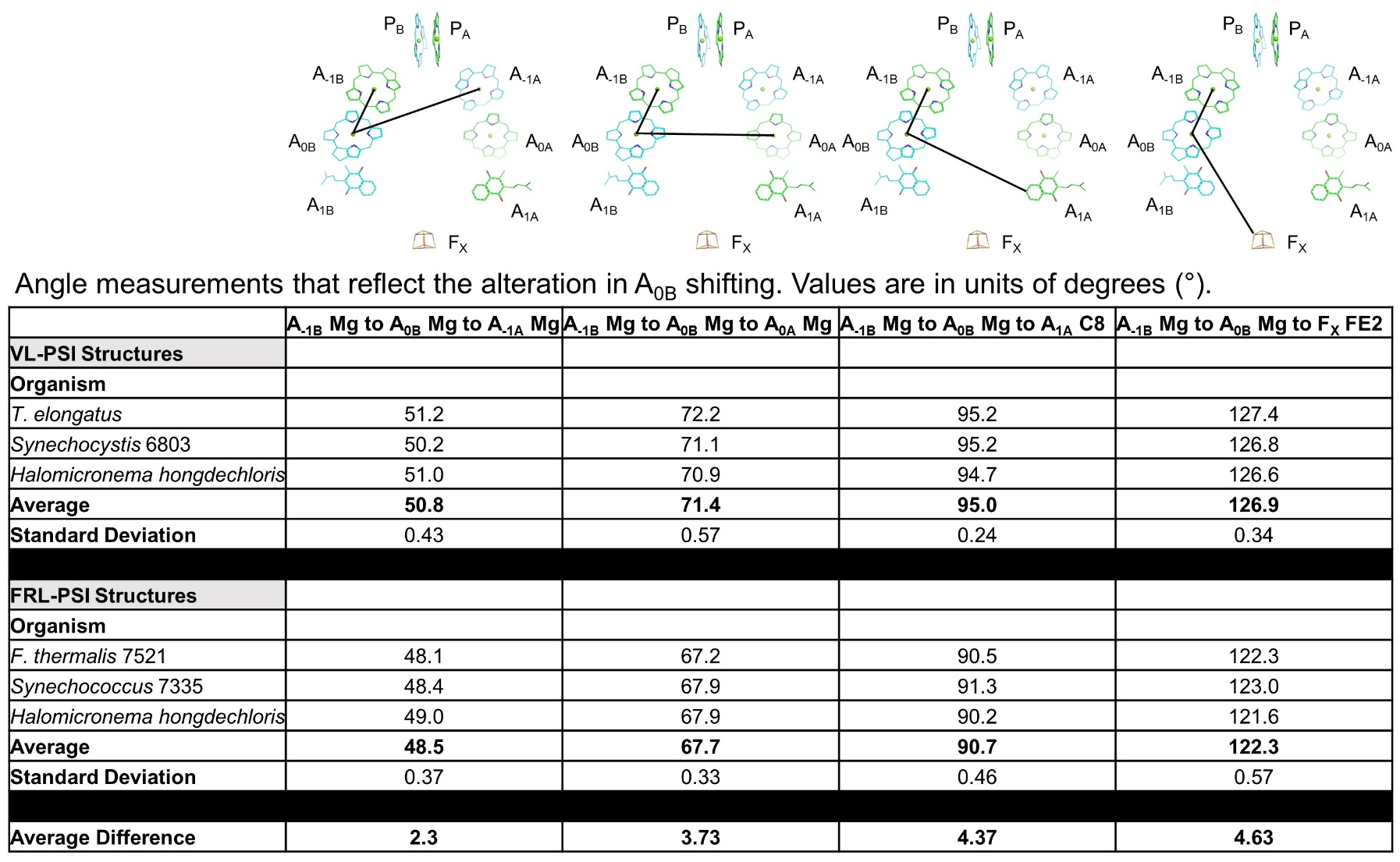


**Supplementary Figure 7.** Angles measured to confirm the rotation of the Chl *a* molecule in site A_0B_ of FRL-PSI structures. The corresponding values are shown in **Supplementary Table 2**.


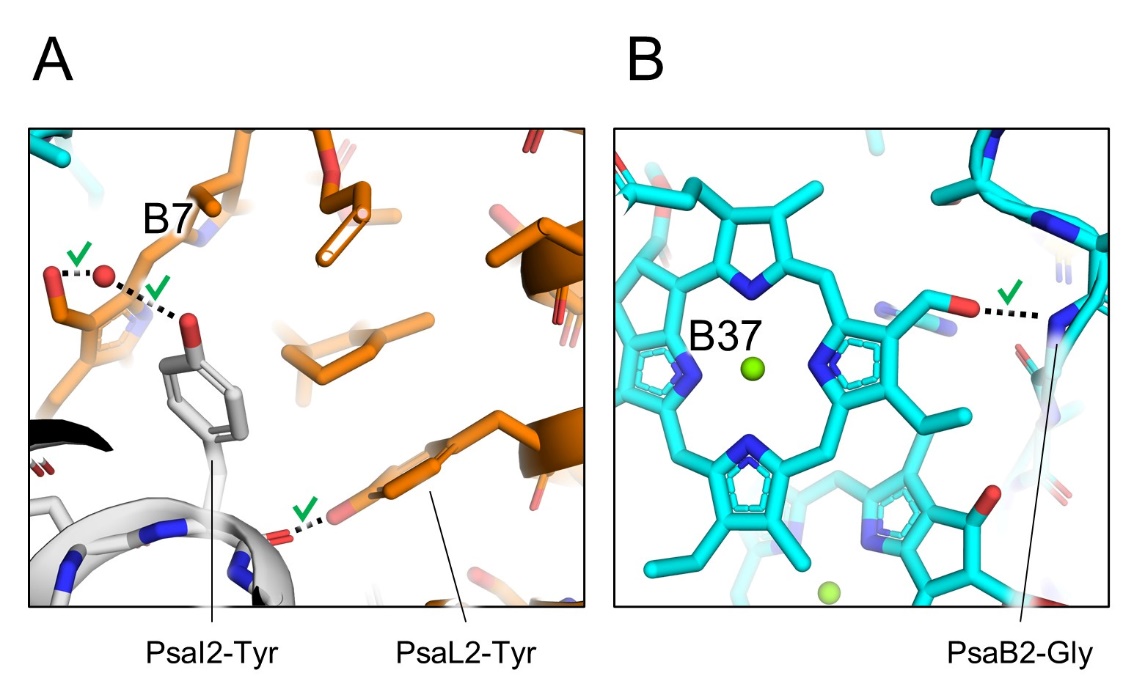


**Supplementary Figure 8.** FRL-specific interactions of cluster 1 that interacts with the formyl moieties of the Chl *f* molecules in sites B7 and B37. **A** The structure of FRL-PSI from *H. hongdechloris* is shown with FRL-specific H-bonding interactions near the C2 formyl moiety of B7. **B** The structure of FRL-PSI from *F. thermalis* PCC 7521 is shown with the FRL-specific H-bonding interaction with the C2 formyl moiety of B37. In both panels, FRL-specific residues are labeled and their H-bonding interactions that are specific to FRL-PSI are shown as dashed lines with a green check.

**
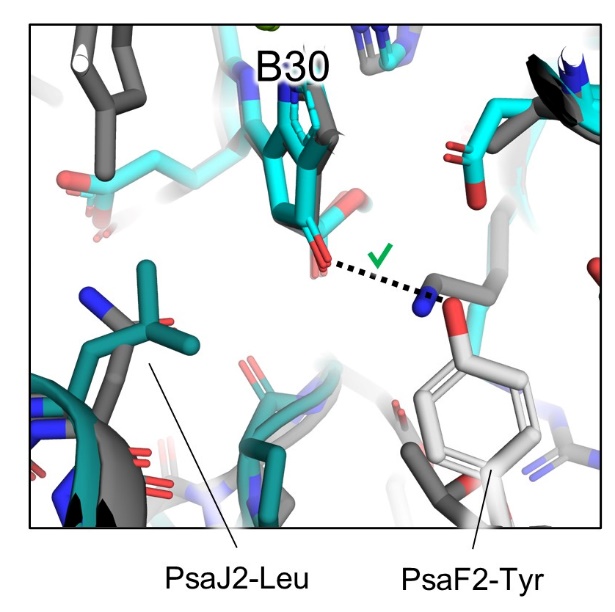
**

**Supplementary Figure 9.** Vicinity of the 13^1^-keto oxygen of the Chl *f* in site B30. The superposition of FRL-PSI from *F. thermalis* PCC 7521 (colors) and PSI from the non-FaRLiP *T. elongatus* (grey) is shown. The FRL-specific H-bonding interaction is shown, and additionally the replacement of an Asn sidechain found in VL structures with a Leu sidechain found in FRL structures.

**
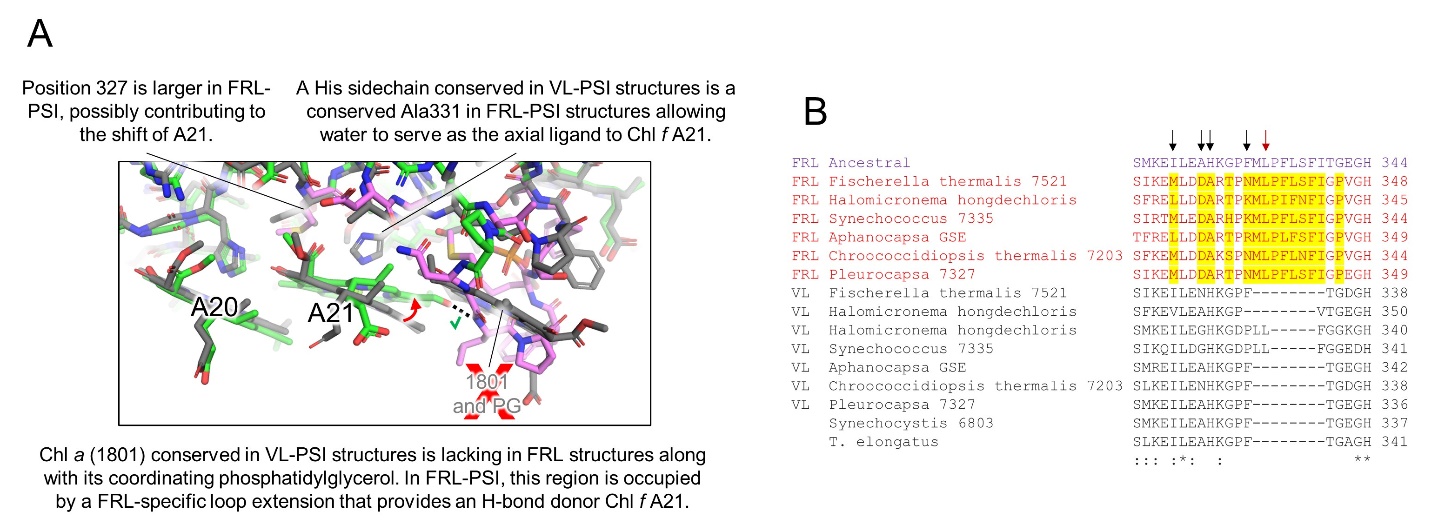
**

**Supplementary Figure 10.** FRL-specific features in the vicinity of cluster 4. **A** A superposition of the FRL-PSI structure from *F. thermalis* PCC 7521 (colored) and the PSI structure from the non-FaRLiP *T. elongatus* (grey) is shown. The FRL-specific loop is shown in pink. Important features described in the text are labeled. **B** Corresponding sequence alignment.

**3 Supplementary Tables**

**Supplementary Table 1.** Number of FRL-specific residues in FRL-PSI subunits.

|  | **Mean number of residues in FRL sequences*** | **Number of FRL-specific residues** | **Number of ancestral residues with PP>0.9 that are consistent with FRL-specific residues** |
| --- | --- | --- | --- |
| **PsaA2** | 785 | 48 (6.1%) | 22 (46%) |
| **PsaB2** | 742 | 14 (1.9%) | 14 (100%) |
| **PsaF2** | 161 | 24 (1.4%) | 16 (67%) |
| **PsaI2** | 62 | 10 (16.1%) | 4 (40%) |
| **PsaJ2** | 46 | 8 (17.4%) | 8 (100%) |
| **PsaL2** | 179 | 26 (14.5%) | 23 (88%) |

*Rounded to the nearest whole number

**Supplementary Table 2.** Angle measurements that show the rotation of A_0B_ in FRL-PSI relative to other PSI structures. The angles correspond to those shown in **Supplementary Figure 9**. Values are in units of degrees (°).

|  | **A_-1B_ Mg to A_0B_ Mg to A_-1A_ Mg** | **A_-1B_ Mg to A_0B_ Mg to A_0A_ Mg** | **A_-1B_ Mg to A_0B_ Mg to A_1A_ C8** | **A_-1B_ Mg to A_0B_ Mg to F_X_ FE2** |
| --- | --- | --- | --- | --- |
| **VL-PSI Structures** |  |  |  |  |
| **Organism** |  |  |  |  |
| *T. elongatus* | 51.2 | 72.2 | 95.2 | 127.4 |
| *Synechocystis* 6803 | 50.2 | 71.1 | 95.2 | 126.8 |
| *H. hongdechloris* | 51.0 | 70.9 | 94.7 | 126.6 |
| **Average** | **50.8** | **71.4** | **95.0** | **126.9** |
| **Standard Deviation** | 0.43 | 0.57 | 0.24 | 0.34 |
|  |  |  |  |  |
| **FRL-PSI Structures** |  |  |  |  |
| **Organism** |  |  |  |  |
| *F. thermalis* 7521 | 48.1 | 67.2 | 90.5 | 122.3 |
| *Synechococcus* 7335 | 48.4 | 67.9 | 91.3 | 123.0 |
| *H. hongdechloris* | 49.0 | 67.9 | 90.2 | 121.6 |
| **Average** | **48.5** | **67.7** | **90.7** | **122.3** |
| **Standard Deviation** | 0.37 | 0.33 | 0.46 | 0.57 |
|  |  |  |  |  |
| **Average Difference** | **2.3** | **3.73** | **4.37** | **4.63** |
