# Supplementary material for: Molecular diversity and evolution of far-red light-acclimated photosystem I
Source: Front Plant Sci. 2023 Nov 20;14:1289199. doi: 10.3389/fpls.2023.1289199 (PMC10694217; doi:10.3389/fpls.2023.1289199)
Supplement: Supplementary file 3 [file Table_1.docx]

Jpred4 output for PsaA2 focusing on the missing FRL-specific loop

QUERY : NNVLDRVLRHRDAIISHLAWVCQFLGFHSFAMYCHNDTMRAFGRPQDMFSDTGIQLQPVFAQWLQHIHTMTIGNPSLQVAAPLGHAFGGLRNLELTGLGTAAPNLHDPVSYAFGGGVVAVGGKVAMMPITLGTADFLIHHIHAFTIHVTVLVLLKGVLFARS : QUERY

[UniRef90_D3EPK7](http://www.ebi.ac.uk/ebisearch/search.ebi?db=allebi&query=UniRef90_D3EPK7) : DNLLDRMIRHRDAIISHLNWVCMFLGFHSFGLYVHNDTMRALGRPQDMFSDSAIQLQPIFAQWLQNIHSIAPTG--------------------------TAPHALATASYAFGGDTIAVANKVAMMPITLGTADFMIHHVHAFTIHVTVLILLKGVLYARS : [UniRef90_D3EPK7](http://www.ebi.ac.uk/ebisearch/search.ebi?db=allebi&query=UniRef90_D3EPK7)

[UniRef90_K9SF66](http://www.ebi.ac.uk/ebisearch/search.ebi?db=allebi&query=UniRef90_K9SF66) : NNVLDRLLRHRDAIISHLNWVCIFLGFHSFGLYIHNDTMRAFGRPQDMFSDTGIQLQPIFAQWIQNIHANTI--------------------------ASTAPYAGDSVSPIFGGDIVALGGKIAMAPMTLGTADFMVHHIHAFTIHVTVLILLKGVLFSRN : [UniRef90_K9SF66](http://www.ebi.ac.uk/ebisearch/search.ebi?db=allebi&query=UniRef90_K9SF66)

[UniRef90_B8PV03](http://www.ebi.ac.uk/ebisearch/search.ebi?db=allebi&query=UniRef90_B8PV03) : DNVLDRIIRHRDAIISHLNWVCIFLGFHSFGLYIHNDTMRALGRSQDMFSDTAIQLQPVFAQWIQNIHTLAP--------------------------SNTSPSALATSSYVFGGDIVAINNKIALMPIKLGTADFMVHHIHAFTIHVCVLILVKGFLFSRN : [UniRef90_B8PV03](http://www.ebi.ac.uk/ebisearch/search.ebi?db=allebi&query=UniRef90_B8PV03)

[UniRef90_G8IVY1](http://www.ebi.ac.uk/ebisearch/search.ebi?db=allebi&query=UniRef90_G8IVY1) : NNLLDRVLRHRDAIVSHLNWVCIFLGFHSFGLYIHNDTMSALGRPQDMFSDTAIQLQPIFAQWIQNTHALAPG--------------------------STAPGATASTSLTWGGDLXXXXXXXXXXPIPLGTADFLVHHIHAFTIHVTVLILLKGVLFARS : [UniRef90_G8IVY1](http://www.ebi.ac.uk/ebisearch/search.ebi?db=allebi&query=UniRef90_G8IVY1)

[UniRef90_UPI00034537F4](http://www.ebi.ac.uk/ebisearch/search.ebi?db=allebi&query=UPI00034537F4) : NNVLDRTLRIRDTIISHLNWVCLFLGFHAFGMYVHNDTMQALGRPQDMFSDTAIQLRPIFAQFIQGIHTATAATIGDTVVN-------------------TAPMAQAGVSPIFGGDVVAVAGKVSMMPMTLGTADFLVHHIHAMTIHITILILLKGVLFARS : [UniRef90_UPI00034537F4](http://www.ebi.ac.uk/ebisearch/search.ebi?db=allebi&query=UPI00034537F4)

[UniRef90_B6CWT6](http://www.ebi.ac.uk/ebisearch/search.ebi?db=allebi&query=UniRef90_B6CWT6) : NNLLDRVVRHRDAIISHLNWVCIFLGFHSFGLYIHNDTMRALGRAPDMFSDTGIPLRPIFAQFIQNIH--------------------------LVAPTNTAPNALTTASYIFGGDIVAIGSKIAIMPMKLGTADFMVHHIHAFTIHVTVLILLKGVLY--- : [UniRef90_B6CWT6](http://www.ebi.ac.uk/ebisearch/search.ebi?db=allebi&query=UniRef90_B6CWT6)

[UniRef90_Q9AL93](http://www.ebi.ac.uk/ebisearch/search.ebi?db=allebi&query=UniRef90_Q9AL93) : DNLLDRVIRHRDAIISHLNWVCIWLGFHSFGLYIHNDTMRALGRPQDMFSDSAIQLQPIFAQGIQSIQAAVAG-------------------------SAQAPWVGAATSPVWGGDTIAVGGKVAMSAIPLGTADFMVHHIHAFTIHVTVLILLKGVLYARN : [UniRef90_Q9AL93](http://www.ebi.ac.uk/ebisearch/search.ebi?db=allebi&query=UniRef90_Q9AL93)

[UniRef90_D9IXP5](http://www.ebi.ac.uk/ebisearch/search.ebi?db=allebi&query=UniRef90_D9IXP5) : DNVLDRVLKHRDTIISHLNWVCIFLGFHSFGLYIHNDTMRALGRSRDMFSDSAISLQPIFAQWVQHLH--------------------------SSAASATAPNTLSTPSYIFGGETIALAGKVAVMPMTLGTADFLVHHIHAFTIHVTALILLKGVLYARS : [UniRef90_D9IXP5](http://www.ebi.ac.uk/ebisearch/search.ebi?db=allebi&query=UniRef90_D9IXP5)

[UniRef90_UPI00046EFE0B](http://www.ebi.ac.uk/ebisearch/search.ebi?db=allebi&query=UPI00046EFE0B) : DNVLDRVLKARDALISHLNWVCIWLGFHSFGLYVHNDTMRAWGRPQDMFSDTGIQLQPIFAQWVQ-------------------------QVNSSLSVASTAPNALSGVSEVFNGSMVAVGGKVAVAPIPLGTADFLVHHIHAFTIHVTVLILLKGVLFARN : [UniRef90_UPI00046EFE0B](http://www.ebi.ac.uk/ebisearch/search.ebi?db=allebi&query=UPI00046EFE0B)

[UniRef90_B0M299](http://www.ebi.ac.uk/ebisearch/search.ebi?db=allebi&query=UniRef90_B0M299) : NNLLDRVIRHRDAIISHLNWVCIFLGLHSFGLYIHNDTISALGRPKDMFSDSAIQIQPIFAQFIQRIHSVAP--------------------------QMTAPSEVFPNSVVWGGNLVNVGGKVAMIPISLGTADFIVHHIHAFTIHVTVLILLKGVLFARS : [UniRef90_B0M299](http://www.ebi.ac.uk/ebisearch/search.ebi?db=allebi&query=UniRef90_B0M299)

[UniRef90_B0C474](http://www.ebi.ac.uk/ebisearch/search.ebi?db=allebi&query=UniRef90_B0C474) : NNVLDRMLRHRDTIISHLNWVCIFLGFHSFGLYIHNDNMRSLGRPQDMFSDTAIQLQPIFSQWVQNLQANVAGTIR-------------------------APLAEGASSLAWGGDPLFVGGKVAMQHVSLGTADFMIHHIHAFQIHVTVLILIKGVLYARS : [UniRef90_B0C474](http://www.ebi.ac.uk/ebisearch/search.ebi?db=allebi&query=UniRef90_B0C474)

[UniRef90_Q06J42](http://www.ebi.ac.uk/ebisearch/search.ebi?db=allebi&query=UniRef90_Q06J42) : NNLLDRIICQRDAIISHLNWACIFLGLHSFGLYIHNDTMSALGRSDDMFSDTAIQLQPIFSQFIQRIHYMTIE--------------------------STAPYVSHGTSPAWGGDIVAINGKIAMMPISLGTSDFMVHHIHAFTIHVTVLILLKGVLFSRN : [UniRef90_Q06J42](http://www.ebi.ac.uk/ebisearch/search.ebi?db=allebi&query=UniRef90_Q06J42)

[UniRef90_Q85FM0](http://www.ebi.ac.uk/ebisearch/search.ebi?db=allebi&query=UniRef90_Q85FM0) : DNVLARMLEHKEAIISHLSWASLFLGFHTLGLYVHNDVMLAFGTPEKQ-----ILIEPVFAQWIQSAHGKVSYGFDVLLSSVDSPASNAGRGLWLP------------------GWLDAVNSSNNSLFLTIGPGDFLVHHAIALGLHTTTLILVKGALDAR- : [UniRef90_Q85FM0](http://www.ebi.ac.uk/ebisearch/search.ebi?db=allebi&query=UniRef90_Q85FM0)

[UniRef90_UPI000303611B](http://www.ebi.ac.uk/ebisearch/search.ebi?db=allebi&query=UPI000303611B) : -NVLDRVLQHKEAIISHLSWVSLFLGFHTLGLYVHNDVVVAFGTPEKQ-----ILIEPVFAQFIQASHGKVLYGMDLLLSDPNSIA------------TTAWPNHGA---VWLPGWLDAINSGANSLFLTIGPGDFLVHHAIALGLHTTTLILVKGALDAR- : [UniRef90_UPI000303611B](http://www.ebi.ac.uk/ebisearch/search.ebi?db=allebi&query=UPI000303611B)

[UniRef90_A7M901](http://www.ebi.ac.uk/ebisearch/search.ebi?db=allebi&query=UniRef90_A7M901) : NDLLDRVLRHRDAIISHLNWVCMFLGFHSFGLYIHNDTMSALGRPQDMFSDTAIQLQPIFAQWIQKIHTF------------------------------LTPDLAANPGLSWSG-VVAVGGKIALAPISLGTADFLVHHIHAFTIHVTVLILLKGVLFARS : [UniRef90_A7M901](http://www.ebi.ac.uk/ebisearch/search.ebi?db=allebi&query=UniRef90_A7M901)

[UniRef90_Q2WGC4](http://www.ebi.ac.uk/ebisearch/search.ebi?db=allebi&query=UniRef90_Q2WGC4) : NNLLDRVLRHRDAIISHLNWACIFLGFHSFGSYIHNDTMSALGRPQDMFPDTAMRLQPTFAQRVQNAHASAP--------------------------VLTAPAANTATSLAWGGGVLAMGGKVVLSAIPLGTADFLVHHIHAFTIHATVLIPPKGVPFARS : [UniRef90_Q2WGC4](http://www.ebi.ac.uk/ebisearch/search.ebi?db=allebi&query=UniRef90_Q2WGC4)

[UniRef90_C3W1X0](http://www.ebi.ac.uk/ebisearch/search.ebi?db=allebi&query=UniRef90_C3W1X0) : NNLLDRVLRHRDAIVSHLNWVCIFLGFHSFGLYIHNDTMSALGRPQDMFSDTAIQLQPIFAQWIQNTHASAPG--------------------------STAPGATASTSLTWGGDLVTVGSKVALLPIPLGTADFLVHHIHAFTIHV-------------- : [UniRef90_C3W1X0](http://www.ebi.ac.uk/ebisearch/search.ebi?db=allebi&query=UniRef90_C3W1X0)

[UniRef90_Q7NFT6](http://www.ebi.ac.uk/ebisearch/search.ebi?db=allebi&query=UniRef90_Q7NFT6) : NNLLDRVLRHRDAIISHLNWVTLFLGFHSFGLYVHNDTMQALGRPRDMFADFAIPLQPVFAQWIQNIHAAAPGGATAPWVGGTSP-------TWYTGALSSAATLQANQVLAL------ANDKISISPIHLGTADFMVHHIFALCIHVTVLILLKGVLFARS : [UniRef90_Q7NFT6](http://www.ebi.ac.uk/ebisearch/search.ebi?db=allebi&query=UniRef90_Q7NFT6)

[UniRef90_Q2WGC5](http://www.ebi.ac.uk/ebisearch/search.ebi?db=allebi&query=UniRef90_Q2WGC5) : -NVLARVLEHKEAIISHLSWASLFLGFHTLGLYVHNDVMLAFGTPEKQ-----ILIEPVFAQWIQSAHGKASYGFDVLLSSPNDPAFNAGR------------------SIWLPGRLDAIDNNSNSLFLTIGPGDFPVHHAIASGLHTTTLILSKGALDAR- : [UniRef90_Q2WGC5](http://www.ebi.ac.uk/ebisearch/search.ebi?db=allebi&query=UniRef90_Q2WGC5)

[UniRef90_A2C4V3](http://www.ebi.ac.uk/ebisearch/search.ebi?db=allebi&query=UniRef90_A2C4V3) : DNVLDRILKARDALISHLNWACMFLGFHSFGLYIHNDVMRALGRPADMFSDTGIQLQPVFAQWIQNIHNSAAGSTTLAGANVNLQPGL--------------------VSEVFNGSVSQVGGKIGIAPIPLGTADFMIHHIHAFTIHVTLLILLKGVLFARS : [UniRef90_A2C4V3](http://www.ebi.ac.uk/ebisearch/search.ebi?db=allebi&query=UniRef90_A2C4V3)

[UniRef90_K9U1I6](http://www.ebi.ac.uk/ebisearch/search.ebi?db=allebi&query=UniRef90_K9U1I6) : -NVLDRVLKHKEAIISHLSWVSLFLGFHTLGIYVHNDVVVAFGTPEKQ-----ILIEPVFAQFIQASHGKVLYGFNTLLSNPDSIASTAGAT-------------------YLPGWYEAINNTTNSLFLTIGPGDFLVHHAFALAIHTTVLVLVKGALDAR- : [UniRef90_K9U1I6](http://www.ebi.ac.uk/ebisearch/search.ebi?db=allebi&query=UniRef90_K9U1I6)

[UniRef90_B9VL88](http://www.ebi.ac.uk/ebisearch/search.ebi?db=allebi&query=UniRef90_B9VL88) : -NVLARILDHKEAIISHLSWASLFLGFHTLGIYVHNDVMLAFGTPEKQ-----ILIEPLFAQWIQGAHGQNIYGFNVLLSNDQN------------------PAALAGQTLWLPGWLSAINNPTNSLFLIIGPGDFLVHHAIALGLHVTTLILVKGALDGR- : [UniRef90_B9VL88](http://www.ebi.ac.uk/ebisearch/search.ebi?db=allebi&query=UniRef90_B9VL88)

[UniRef90_Q06J40](http://www.ebi.ac.uk/ebisearch/search.ebi?db=allebi&query=UniRef90_Q06J40) : DNVLARFLENREVIISHLSWVCLFLGFHTLGLYVHNDVMLAFETPEKQ-----ILIEPIFAQYIQAAQGKSSYDFNVLLSSSVSDA-------------YLIPTQSANKGIWLSGWLNSINNNTNSLFIEIGPGDFLVHHAIALGLHTTTLILVKGALDAR- : [UniRef90_Q06J40](http://www.ebi.ac.uk/ebisearch/search.ebi?db=allebi&query=UniRef90_Q06J40)

[UniRef90_A9BCK8](http://www.ebi.ac.uk/ebisearch/search.ebi?db=allebi&query=UniRef90_A9BCK8) : DNVLDRMLKARDAIISHLNWVCMWLGFHSFGLYIHNDVMRALGRPQDMFSDTGIQLQPFLAQWVQNLQQSAVG------------------TGQLVGAGNLPGNVL---SEVFNGNVIEVGGKVAIGPIPLGTADLMIHHVHAFTIHVTLLILLKGVLYSRS : [UniRef90_A9BCK8](http://www.ebi.ac.uk/ebisearch/search.ebi?db=allebi&query=UniRef90_A9BCK8)

[UniRef90_D1J7A4](http://www.ebi.ac.uk/ebisearch/search.ebi?db=allebi&query=UniRef90_D1J7A4) : DNVLARMLEHKEAIISHLSWVSLFLGFHTLGLYIHNDTVVAFGQPEKQ-----ILVEPVFAQFIQAASGKAVYGFDLLLSSKESPASAAGSEIWLP------------------GWINAINNDKNDLFLTIGPGDFLIHHAIALGLHTTTLILVKGALDAR- : [UniRef90_D1J7A4](http://www.ebi.ac.uk/ebisearch/search.ebi?db=allebi&query=UniRef90_D1J7A4)

[UniRef90_B9TU98](http://www.ebi.ac.uk/ebisearch/search.ebi?db=allebi&query=UniRef90_B9TU98) : -NVLSRVLDHKEAIISHLSWACLFLGFHTLGLYIHNDVMLAFGTPEKQ-----ILIEPLFAQWIQAAHGKSIYGFDVFLSSNLSPAYNASQTLWLPGWLSAINGASGPGT----------------LFLIIGPGDFLVHHAIALGLHTTTLILVKGALDAR- : [UniRef90_B9TU98](http://www.ebi.ac.uk/ebisearch/search.ebi?db=allebi&query=UniRef90_B9TU98)

[UniRef90_B9TUA0](http://www.ebi.ac.uk/ebisearch/search.ebi?db=allebi&query=UniRef90_B9TUA0) : NNVLARILDHKEAIISHLSWVSLFLGFHTLGLYCHNDVMQAFGTPEKQ-----ILIEAIFAQFIQAAHGKSLYGFQVFLSSSD------------------ALTTLAAQNIWLPGWLEAINDESNSLFFAIGPGDFLVHHAIALGLHTTVLILVKGALDAR- : [UniRef90_B9TUA0](http://www.ebi.ac.uk/ebisearch/search.ebi?db=allebi&query=UniRef90_B9TUA0)

[UniRef90_W6MDD2](http://www.ebi.ac.uk/ebisearch/search.ebi?db=allebi&query=UniRef90_W6MDD2) : DNVLARMLEHKEAIISHLSWVTLFLGFHTLGLYVHNDVMQAFGTPEKQ-----ILIEPVFAQWIQSAQGKVAYAANSVSGDP----------FSLLLASSDSVAYSNSNAIYLPGWLSAINNPNNSLFLTIGPGDFLVHHAIALGLHTTTLILVKGALDAR- : [UniRef90_W6MDD2](http://www.ebi.ac.uk/ebisearch/search.ebi?db=allebi&query=UniRef90_W6MDD2)

[UniRef90_B0C475](http://www.ebi.ac.uk/ebisearch/search.ebi?db=allebi&query=UniRef90_B0C475) : NNVLARALEHKEAIISHLSWVSMFSGFHTLGVYVHNDTVVAFGTPEKQ-----ILVEPIFAQWIQAAHGKLLLGFETLLSNPNGLA-------------YNPPNISPDVFV--PGWVEAMNNPVIGPFMSQGPGDFLVHHGIAFSLHVTVLICVKGCLDAR- : [UniRef90_B0C475](http://www.ebi.ac.uk/ebisearch/search.ebi?db=allebi&query=UniRef90_B0C475)

[UniRef90_A8G724](http://www.ebi.ac.uk/ebisearch/search.ebi?db=allebi&query=UniRef90_A8G724) : -NVLDRILKARDALISHLNWVCMWLGFHSFGLYIHNDTMRALGRPQDMFSDKAIQLQPVFAQWVQNIQSSGIGTTLL---------------------------EGNGVSQVFNGDTITIGGKIAMKAIPLGTADLMIHHIHAFQIHVCVLILLKGVLYSRN : [UniRef90_A8G724](http://www.ebi.ac.uk/ebisearch/search.ebi?db=allebi&query=UniRef90_A8G724)

[UniRef90_I6NIT4](http://www.ebi.ac.uk/ebisearch/search.ebi?db=allebi&query=UniRef90_I6NIT4) : -----RILNHKEAIISHLSWVTLFLGFHTLGLYVHNDVMQAFGTPEKQ-----ILIEPVFAQWIQSAHGKGLYGFDFLLSLSTSNASN------------------ASESIWLPGWLNAINDQSGSLFLQIGPGDFLV-HAIALGLHTTTLILVKGALDAR- : [UniRef90_I6NIT4](http://www.ebi.ac.uk/ebisearch/search.ebi?db=allebi&query=UniRef90_I6NIT4)

[UniRef90_UPI00046F268C](http://www.ebi.ac.uk/ebisearch/search.ebi?db=allebi&query=UPI00046F268C) : NNVLARMLEHKEALISHLSWASLFLGFHTLGLYVHNDVVVAFGTPEKQ-----ILVEPVFAQFIQASHGKVMYGFDVLLANANSAATMASQNIPGPHY-----------------WLDAINGSTDV-FLPIGPGDFLVHHAIALGLHTTTLILVKGALDAR- : [UniRef90_UPI00046F268C](http://www.ebi.ac.uk/ebisearch/search.ebi?db=allebi&query=UPI00046F268C)

[UniRef90_E1CID8](http://www.ebi.ac.uk/ebisearch/search.ebi?db=allebi&query=UniRef90_E1CID8) : -NVLARILIHKEVLISHLSWVSLFLGFHTLGLYVHNDVIQAFGTPERQ-----ILIEPIFAQWIQSAQGKVRYGFNYFLSSPVNPASMASQNLWLPY------------------WLTAINDTSTSLFLPIGPGDFLVHHAISLGVHVTTLILVKGALDAR- : [UniRef90_E1CID8](http://www.ebi.ac.uk/ebisearch/search.ebi?db=allebi&query=UniRef90_E1CID8)

[UniRef90_C7EDU6](http://www.ebi.ac.uk/ebisearch/search.ebi?db=allebi&query=UniRef90_C7EDU6) : DNVLGRMLEHKEAIISHLSWVSLFLGFHTLGVYVHNDVVMAFGHPERQ-----ILIEPIFAQWIQAASGKMMYGLSFLLSDPNSAASLAADSMPGDH-----------------FWMNAINDKSNSLFLPIGSADLLVHHAIALGLHTTTLILVKGALDAR- : [UniRef90_C7EDU6](http://www.ebi.ac.uk/ebisearch/search.ebi?db=allebi&query=UniRef90_C7EDU6)

[UniRef90_D9IXP4](http://www.ebi.ac.uk/ebisearch/search.ebi?db=allebi&query=UniRef90_D9IXP4) : -NVLARVLAHKETIISHLSWVSLFLGFHTLGLYVHNDVVVAFGQAEKQ-----ILVEPVFAQLIQASSGKLVYNLQWWLSDPTLAAGDSLLTPISSAP-------------WIDGWLKQVNSNANSIFLEIGPGDFLVHHAIALGLHTTTLILVKGALDAR- : [UniRef90_D9IXP4](http://www.ebi.ac.uk/ebisearch/search.ebi?db=allebi&query=UniRef90_D9IXP4)

[UniRef90_Q7NFT5](http://www.ebi.ac.uk/ebisearch/search.ebi?db=allebi&query=UniRef90_Q7NFT5) : NNVLARMLEHKEALISHLSWVSLFLGFHTLGLYVHNDVMLAFGRPEDQL-----LIEPVFAQFVQVQSGKIIEGIPALFGGPGVTAPGEFLT----------------------GWLGSVNANNSPIFLPIGPGDFLVHHAIALGLHTTTLILVKGALDAR- : [UniRef90_Q7NFT5](http://www.ebi.ac.uk/ebisearch/search.ebi?db=allebi&query=UniRef90_Q7NFT5)

[UniRef90_M5DDL6](http://www.ebi.ac.uk/ebisearch/search.ebi?db=allebi&query=UniRef90_M5DDL6) : NNLLDRVLRHRDAIISHLNWVCIFLGFHSFGLYIHNDTMSALGRPQDMFSDTAIQSQPIFAQWVQNTHASAPNP--------------------------TAPNATAGTSLTWGGDLXXXXXXXXXXXXXXXXXXXXXXXXHAFTIHVTVLILLKGVSFARS : [UniRef90_M5DDL6](http://www.ebi.ac.uk/ebisearch/search.ebi?db=allebi&query=UniRef90_M5DDL6)

[UniRef90_I6NMH7](http://www.ebi.ac.uk/ebisearch/search.ebi?db=allebi&query=UniRef90_I6NMH7) : NNLLDRVLRHRDSIIVHLNWVCIFLGTHAFGFYIHNDTMRALGRPQDMFSDKAIQLQPIFAQWIQNIHLLAPG--------------------------TTAPNALATTSYAFGGEVVEVGGKIAMMPIKLGTADFMVHH---------------------- : [UniRef90_I6NMH7](http://www.ebi.ac.uk/ebisearch/search.ebi?db=allebi&query=UniRef90_I6NMH7)

[UniRef90_A0A023PLZ6](http://www.ebi.ac.uk/ebisearch/search.ebi?db=allebi&query=UniRef90_A0A023PLZ6) : -NVLARMLEHKEAIISHLSWVSLFLGFHTLGIYVHNDVVVAFGQPEKQ-----ILVEPLFAEWIQAASGKTLYNFDLLLASSTSSASVASSQLWLP------------------GWLSAINDGKNSLFLPIGPGDFLVHHAIALGLHTTTLILVKGALDAR- : [UniRef90_A0A023PLZ6](http://www.ebi.ac.uk/ebisearch/search.ebi?db=allebi&query=UniRef90_A0A023PLZ6)

[UniRef90_B9TUA3](http://www.ebi.ac.uk/ebisearch/search.ebi?db=allebi&query=UniRef90_B9TUA3) : NNVLSRVLEHKEAIISHLSWVSLFLGFHTLSLYVHNDVVVAFGTPEKQ-----ILIEPVFAQFVQAASGKALYGMDTLLSNPDSIAHTAGR---------------------LATWLVRSNSGTNSLFLTIGPGDFLVHHAIALGLHTTTLILVKGALDAR- : [UniRef90_B9TUA3](http://www.ebi.ac.uk/ebisearch/search.ebi?db=allebi&query=UniRef90_B9TUA3)

[UniRef90_A2CC73](http://www.ebi.ac.uk/ebisearch/search.ebi?db=allebi&query=UniRef90_A2CC73) : DNVLDRMFKARDAIISHLNWVCMFLGFHSFGLYIHNDSMRALGRSQDMFSDSAIQLQPVLAQWIQSLWASSIG------------------TSSVVGTTTGLPGA---VSDVFNGGVVAVGGKVALMAIPLGTADLMIHHIHAFTIHVTCLILLKGVLFARS : [UniRef90_A2CC73](http://www.ebi.ac.uk/ebisearch/search.ebi?db=allebi&query=UniRef90_A2CC73)

[UniRef90_M0UB87](http://www.ebi.ac.uk/ebisearch/search.ebi?db=allebi&query=UniRef90_M0UB87) : NDLLDRVLRHRDAIISHLNWAW---------------------RPQDMFSDTAIQLQPIFAQWVQNTHALAPG--------------------------ITAPGATASTSLTWGGGLVAVGGKVALLPIPLGTADFLVHHIHAFTIHVTVLILLKGVLFARS : [UniRef90_M0UB87](http://www.ebi.ac.uk/ebisearch/search.ebi?db=allebi&query=UniRef90_M0UB87)

[UniRef90_UPI000248498A](http://www.ebi.ac.uk/ebisearch/search.ebi?db=allebi&query=UPI000248498A) : -NALSRLLEHKEAIISHLSWVSMFLGFHTLDLYVHNDVVVAFGTPEKQ-----ILPEPIFAEWVQAAHGKLLLGLDSLLSNPQSIA------------STAWPNY---GDVWLPGWLDAVNGA-NTPFLNIGPGDFLVHHGIAFSIHVTVLICVKGCLDAR- : [UniRef90_UPI000248498A](http://www.ebi.ac.uk/ebisearch/search.ebi?db=allebi&query=UPI000248498A)

[UniRef90_Q9TJ81](http://www.ebi.ac.uk/ebisearch/search.ebi?db=allebi&query=UniRef90_Q9TJ81) : -NLLDRVLLHRTTLIAHLNWVCIFLGTHSFGLYAHNDTMRALGRPQDTFSDGAISLSPIFAKWIQALHAEAPG--------------------------TTAPNALAVSSLCFLDGTIAVNGRIAIQPIFLGTADFMIHHIHAFQIHVATLILLKGVLFARS : [UniRef90_Q9TJ81](http://www.ebi.ac.uk/ebisearch/search.ebi?db=allebi&query=UniRef90_Q9TJ81)

[UniRef90_B7T4F0](http://www.ebi.ac.uk/ebisearch/search.ebi?db=allebi&query=UniRef90_B7T4F0) : -NVLDRIIKSRDAIISHLNWVCIFLGFHSFGLYIHNDTMRALGRPNDLFSDTAIQLQPVFAQSIQRLHAS-----------------------------------LYNVSDVFGGTTTMVGGKIANAPFTLGTADFMIHHIHAFQIHVVALILIKGVLYSRN : [UniRef90_B7T4F0](http://www.ebi.ac.uk/ebisearch/search.ebi?db=allebi&query=UniRef90_B7T4F0)

[UniRef90_Q05KM1](http://www.ebi.ac.uk/ebisearch/search.ebi?db=allebi&query=UniRef90_Q05KM1) : ---------------------------HSFGLYIHNDTMSALGRPQDMFSDTAIQLQPVFAQWIQNTHFLAP--------------------------QLTAPNALAATSLTWGGDLVAVGGKVAMMPISLGTSDFMVHHIHAFTIHVTVLILLKGVLFSRS : [UniRef90_Q05KM1](http://www.ebi.ac.uk/ebisearch/search.ebi?db=allebi&query=UniRef90_Q05KM1)

[UniRef90_A2CC72](http://www.ebi.ac.uk/ebisearch/search.ebi?db=allebi&query=UniRef90_A2CC72) : DNVLARVLETKEALISHLSWVCMLLGFHTLALYLHNDVVIAFGTPEKQ-----ILVEPIFAQFIQAASGKVMYGLDVLLANANSAPSLA--------------AAGMPGDHYWMDLINASPEVSNFMPI--GPGDFLVHHGIALGLHTTALILIKGALDAR- : [UniRef90_A2CC72](http://www.ebi.ac.uk/ebisearch/search.ebi?db=allebi&query=UniRef90_A2CC72)

[UniRef90_K4KB92](http://www.ebi.ac.uk/ebisearch/search.ebi?db=allebi&query=UniRef90_K4KB92) : NDLLDRVLRHRDAIVSHLNWVCIFLGFHSFGLYIHNDTMSALGRPQDMFSDTAIQLQPVFAQWIXXXXXXXXXXXXXXXXXXXXXXXXXXXXXXXXXXXX-------------------------XXXXPLGTADFLVHHIHAFTIHVTVLILLKGVLFARS : [UniRef90_K4KB92](http://www.ebi.ac.uk/ebisearch/search.ebi?db=allebi&query=UniRef90_K4KB92)

[UniRef90_L0BA64](http://www.ebi.ac.uk/ebisearch/search.ebi?db=allebi&query=UniRef90_L0BA64) : ----------------HLSWVSLFLGFHTLGIYVHNDVVQAFGTPEKQ-----ILIEPVFAQWIQAAHGKTLYGFDLLLSSSTSPAF------------------TASQSLWLPGWVDAINNNTNSLFLTIGPGDFLIHHAIALGLHTTTLILVKGALDAR- : [UniRef90_L0BA64](http://www.ebi.ac.uk/ebisearch/search.ebi?db=allebi&query=UniRef90_L0BA64)

[UniRef90_Q52W90](http://www.ebi.ac.uk/ebisearch/search.ebi?db=allebi&query=UniRef90_Q52W90) : NNLLDRVIRHRDAIISHLNWVSIFLGFHSFGLYIHNDTMRALGRPQDMFSDTAIQLQPIFAQWIQGLHAYAPGQ--------------------------TAPNALAPASYAFGGEIVSVAGKVAILPISLG------------------------------ : [UniRef90_Q52W90](http://www.ebi.ac.uk/ebisearch/search.ebi?db=allebi&query=UniRef90_Q52W90)

[UniRef90_Q8MAH8](http://www.ebi.ac.uk/ebisearch/search.ebi?db=allebi&query=UniRef90_Q8MAH8) : NNVLDRVIRHRDAIISHLNWVCIFLGFHSFGLYIHNDTMRALGRSQDMFSDTAVSLQPIFAQWIQNLHTLAPGN--------------------------TAPNILTTASYAFGGETIAVNGKIAMMPIQLG------------------------------ : [UniRef90_Q8MAH8](http://www.ebi.ac.uk/ebisearch/search.ebi?db=allebi&query=UniRef90_Q8MAH8)

[UniRef90_Q9RDV0](http://www.ebi.ac.uk/ebisearch/search.ebi?db=allebi&query=UniRef90_Q9RDV0) : -NVLARVLETKEALISHLSWVTMLLGFHTLGIYVHNDVVVAFGNPEKQ-----ILVEPVFAQAIQAFSGKVMYGINALLANANSSATLAANSM--------------PGNHYWMDMINRQDALTNFLPI--GPADFLVHHAIALGLHTTALILIKGALDAR- : [UniRef90_Q9RDV0](http://www.ebi.ac.uk/ebisearch/search.ebi?db=allebi&query=UniRef90_Q9RDV0)

[UniRef90_E1CID6](http://www.ebi.ac.uk/ebisearch/search.ebi?db=allebi&query=UniRef90_E1CID6) : NNVLDRVIRHRDSIIAHLNWVCIFLGLHSFGLYIHNDTMSALGRPQDMFTDTAIQLQPVFAQFVQNTH--------------------------LVAPQLTAPGADLATSLSWGGDIVAVGGKVAMMPISLGTADFLVHHI--------------------- : [UniRef90_E1CID6](http://www.ebi.ac.uk/ebisearch/search.ebi?db=allebi&query=UniRef90_E1CID6)

[UniRef90_Q8MAG2](http://www.ebi.ac.uk/ebisearch/search.ebi?db=allebi&query=UniRef90_Q8MAG2) : NNLLDRVLRHREAIISHLNWVCIFLGMHSFGLYIHNDTMRALGRSKDMFSDSAIKLEPVFAKWIQSFHTIANQG--------------------------TAPNALATTSYAFGGDVVSVNGKVAMMPIYLG------------------------------ : [UniRef90_Q8MAG2](http://www.ebi.ac.uk/ebisearch/search.ebi?db=allebi&query=UniRef90_Q8MAG2)

[UniRef90_UPI0003D2784A](http://www.ebi.ac.uk/ebisearch/search.ebi?db=allebi&query=UPI0003D2784A) : NDLLDRVLRHRDAIISHLNWACIFLGFHSFGLYIHNDTMSALGRPQDMFSDTAIQLQPVFAQWIQNTHALAPG--------------------------ATAPGATASTSLTWGGDLVAVG----------------------------------------- : [UniRef90_UPI0003D2784A](http://www.ebi.ac.uk/ebisearch/search.ebi?db=allebi&query=UPI0003D2784A)

[UniRef90_G9BYW1](http://www.ebi.ac.uk/ebisearch/search.ebi?db=allebi&query=UniRef90_G9BYW1) : NNLLDRVLRHREAIISHLNWVCIFLGLHSFGLYIHNDTMRALGRPQDMFSDTAISLQPVFAKAIQNFHLLAPG--------------------------TTAPNALTTASYAFGGDTVSIGNKIAMTPITLG------------------------------ : [UniRef90_G9BYW1](http://www.ebi.ac.uk/ebisearch/search.ebi?db=allebi&query=UniRef90_G9BYW1)

[UniRef90_T2JWX0](http://www.ebi.ac.uk/ebisearch/search.ebi?db=allebi&query=UniRef90_T2JWX0) : ---------------------SLFLGFHTLGLYVHNDVVVAFGTPEKQ-----ILIEPVFAQFVQAASGKALYGFDTLLSNPDSLATGGF----------VAP-------VYLQGWLDAINSGSNSLFLSIGPGDFLVHHAIALGLHTTTLILVKGALDAR- : [UniRef90_T2JWX0](http://www.ebi.ac.uk/ebisearch/search.ebi?db=allebi&query=UniRef90_T2JWX0)

[UniRef90_G9BYT6](http://www.ebi.ac.uk/ebisearch/search.ebi?db=allebi&query=UniRef90_G9BYT6) : NNLLDRMIRHRDAIISHLNWVCIFLGTHSFGLYVHNDTMRALGRSQDMFSDKAIQLQPIFAQFVQN--------------------------VNLSAPGNTAPNQLATASYAFGGDVLAINGKIAMMPITLG------------------------------ : [UniRef90_G9BYT6](http://www.ebi.ac.uk/ebisearch/search.ebi?db=allebi&query=UniRef90_G9BYT6)

[UniRef90_Q9XQV3](http://www.ebi.ac.uk/ebisearch/search.ebi?db=allebi&query=UniRef90_Q9XQV3) : NSIIQQLLAHRDIIMGHLIYVTIALGMHAFGIYIHNDTLQALGRPEDIFSDNSIQLKPLFAVWVQSLPSLFLLNTLSGDAAVTGIP---------------------------GFGLEVLDGKVVTMTQELGTADFMVHHIHAFTIHCTLLILMKGVLYSRS : [UniRef90_Q9XQV3](http://www.ebi.ac.uk/ebisearch/search.ebi?db=allebi&query=UniRef90_Q9XQV3)

[UniRef90_K3Z2T0](http://www.ebi.ac.uk/ebisearch/search.ebi?db=allebi&query=UniRef90_K3Z2T0) : DNVLARMLDHKEAIISHLSWASLFLGFHTLGLYVHNDVMLAFGTPEKQ-----ILIEPIFAQWIQSAHGKTTYG----------------------------------RSIWLPGWLNAVNENSNSLFLTIGPGDFLVHHAIAL-----------GCLDAR- : [UniRef90_K3Z2T0](http://www.ebi.ac.uk/ebisearch/search.ebi?db=allebi&query=UniRef90_K3Z2T0)

[UniRef90_Q8MAG7](http://www.ebi.ac.uk/ebisearch/search.ebi?db=allebi&query=UniRef90_Q8MAG7) : NNLLDRVIRHRDAIISHLNWICIFLGFHSFGLYIHNDTMRALGRTQDMFSDTAIQLKPVFAQWVQNIHTVAPGN--------------------------TSPNSLATASYAFGGDIISVG----------------------------------------- : [UniRef90_Q8MAG7](http://www.ebi.ac.uk/ebisearch/search.ebi?db=allebi&query=UniRef90_Q8MAG7)

[UniRef90_Q8HDB7](http://www.ebi.ac.uk/ebisearch/search.ebi?db=allebi&query=UniRef90_Q8HDB7) : --------------------------------YVHNDVMEAFGTPEKQ-----ILIEPVFAQWIQAAHGKALYGFDFLLSSTNSSAFSNSQ------------------SLWLPGWLEAINNNQNSLFLTIGPGDFLVHHAIALGLHTTTLILVKGALDAR- : [UniRef90_Q8HDB7](http://www.ebi.ac.uk/ebisearch/search.ebi?db=allebi&query=UniRef90_Q8HDB7)

[UniRef90_B6CWT2](http://www.ebi.ac.uk/ebisearch/search.ebi?db=allebi&query=UniRef90_B6CWT2) : -NLLDXXIXHXDAIISHLNWVCXFLXFHSFGXYIHNXTXRALGRXXXMFSDXXXPLRPIXAXFIXNXHXAXP--------------------------TSTAXXAXXTXXXXXGGDILAIGSKIAIMPMKLGTADFMVHHIHAFTIHVTVLILLKGVLY--- : [UniRef90_B6CWT2](http://www.ebi.ac.uk/ebisearch/search.ebi?db=allebi&query=UniRef90_B6CWT2)

[UniRef90_Q9XQV2](http://www.ebi.ac.uk/ebisearch/search.ebi?db=allebi&query=UniRef90_Q9XQV2) : EDIIGRVLAHKAAIISHLSWVSLWLGFHTLGVYIHNDTVTAFGEPQ-----NSILIEPIFAQIIQSASGKTLYGTTLFSVVNPSS-----------------------------GWVQSVNKSFGSLLLPIGPGDLLAHHAIALGLHVTVLILMKGALDAR- : [UniRef90_Q9XQV2](http://www.ebi.ac.uk/ebisearch/search.ebi?db=allebi&query=UniRef90_Q9XQV2)

[UniRef90_G9BYU1](http://www.ebi.ac.uk/ebisearch/search.ebi?db=allebi&query=UniRef90_G9BYU1) : NTLIERTLRHRDAIISHLNWVCIFLGCHSFGLYIHNDTMRALGRSQDMFSDRAIVLKPIFADFIQHIQTVVP--------------------------SITAPNALTTASYAFGGDTITVGSKIALAPIPLG------------------------------ : [UniRef90_G9BYU1](http://www.ebi.ac.uk/ebisearch/search.ebi?db=allebi&query=UniRef90_G9BYU1)

[UniRef90_D7KRB4](http://www.ebi.ac.uk/ebisearch/search.ebi?db=allebi&query=UniRef90_D7KRB4) : NDLLDRVLRHRDAIISHLNWVCIFLGFHSFGLASTRYVFR-------------------------YCYTI---------------------TTSLCSMDTKYPCFSTCLT-WGGGELVAVGGKVALLPIPLGTADFLVHHIHAFTIHVTVLILLKGVLFARS : [UniRef90_D7KRB4](http://www.ebi.ac.uk/ebisearch/search.ebi?db=allebi&query=UniRef90_D7KRB4)

[UniRef90_I1J2J1](http://www.ebi.ac.uk/ebisearch/search.ebi?db=allebi&query=UniRef90_I1J2J1) : --------------------------------------MLAFGTPEKQ-----ILIEPIFAQWIQSAHGKTTYGFDILLSSTNGPAFNAGR------------------SLWLPGWLNAVNENSNSLFLTIGPGDFLVHHAIALGLHTTTLILVKGALDAR- : [UniRef90_I1J2J1](http://www.ebi.ac.uk/ebisearch/search.ebi?db=allebi&query=UniRef90_I1J2J1)

[UniRef90_J7IGD4](http://www.ebi.ac.uk/ebisearch/search.ebi?db=allebi&query=UniRef90_J7IGD4) : DNVLARMLDHKEAIISHLSWASLFLGFHTLGLYVHNDVMLAFGTPEKQ-----ILIEPIFAQWIQSAHGKTAYGFDVLLSSTNGPAFNAGRSIWL------------------------------------------------------------------- : [UniRef90_J7IGD4](http://www.ebi.ac.uk/ebisearch/search.ebi?db=allebi&query=UniRef90_J7IGD4)

[UniRef90_E0X092](http://www.ebi.ac.uk/ebisearch/search.ebi?db=allebi&query=UniRef90_E0X092) : -NVLARILDHKEAIISHLSWVSLFLGFHTLGLYVHNDVMLAFGTPEKQ-----ILIEPVFAQWIQAAQGKTLYGYDLLLSASASPAYTAGQTLWLPGWLDA------------------------------------------------------------- : [UniRef90_E0X092](http://www.ebi.ac.uk/ebisearch/search.ebi?db=allebi&query=UniRef90_E0X092)

[UniRef90_Q2I6P3](http://www.ebi.ac.uk/ebisearch/search.ebi?db=allebi&query=UniRef90_Q2I6P3) : ---VELILAHRDVIISHLVWASVSLGLHSYGLYIHNDTMQALGRPQDIFTDNSICLRPVFGCFI---------------------------------------------SRFLGSDVEVLETKVVRTALQLGTADFLVHHIHAFTIHTTVLILVKGLLFSRS : [UniRef90_Q2I6P3](http://www.ebi.ac.uk/ebisearch/search.ebi?db=allebi&query=UniRef90_Q2I6P3)

[UniRef90_D9IXI2](http://www.ebi.ac.uk/ebisearch/search.ebi?db=allebi&query=UniRef90_D9IXI2) : NSLVALCLNYRDAIISHLNWLCIFLGLHSFGIYIHNDTLAALGR----FDDQITNLPPLGAEWFQHAVTANF---------PINNGFKNHFNTQIL-----------------------MNDKIVFSNLSFNTADFLVHHIHAFTIHVTVLILVKGILFSR- : [UniRef90_D9IXI2](http://www.ebi.ac.uk/ebisearch/search.ebi?db=allebi&query=UniRef90_D9IXI2)

[UniRef90_B9TUA2](http://www.ebi.ac.uk/ebisearch/search.ebi?db=allebi&query=UniRef90_B9TUA2) : NNVLSRVLEHKEAIISHLSWVSLFLGFHTLSLYVHNDVVVAFGTPEKQ-----ILIEPVFAQFVQAASGKALYGMDTLLSNPDSIAQ--------------------TGAVWLPGWLDAINSGTNSLFLTI------------------------------- : [UniRef90_B9TUA2](http://www.ebi.ac.uk/ebisearch/search.ebi?db=allebi&query=UniRef90_B9TUA2)

[UniRef90_M1WY86](http://www.ebi.ac.uk/ebisearch/search.ebi?db=allebi&query=UniRef90_M1WY86) : -----------------------------------------------MFSDASIKLQPVFAQWLQNLHTMAPG-------------------------SSTAPNALTTVSHAFGGGAVAIGGKVAMMPIALGTADFMVHHIHAFTIHVTVLILLKGLLFARS : [UniRef90_M1WY86](http://www.ebi.ac.uk/ebisearch/search.ebi?db=allebi&query=UniRef90_M1WY86)

[UniRef90_W1P9T5](http://www.ebi.ac.uk/ebisearch/search.ebi?db=allebi&query=UniRef90_W1P9T5) : ------------------------------------------GRLGRAHKEKQILIEPIFAQWIQSAHGNTSYGFDVLLSSTNGPAFNAGQ------------------SLWLPSWLNVINENGNSLFLTISPGDFLVHHVISLGLHTATLILVKGALDAR- : [UniRef90_W1P9T5](http://www.ebi.ac.uk/ebisearch/search.ebi?db=allebi&query=UniRef90_W1P9T5)

[UniRef90_Q8MAF3](http://www.ebi.ac.uk/ebisearch/search.ebi?db=allebi&query=UniRef90_Q8MAF3) : DNLLDRILKHRDIIIAHLNWVCIFLGYHSFGFYIHNDIMFALGRXGDAFSDNAIQLQPIFAQCIQLLHTNIPH--------------------------ITAPNILLGISYVFSGDSLLVNGQIAVGIIVLS------------------------------ : [UniRef90_Q8MAF3](http://www.ebi.ac.uk/ebisearch/search.ebi?db=allebi&query=UniRef90_Q8MAF3)

[UniRef90_P58309](http://www.ebi.ac.uk/ebisearch/search.ebi?db=allebi&query=UniRef90_P58309) : ------VLTQRDIILGHLTWVVAFLGVHSFGLYVHNDTMQALGRPDDMFSDNAISLLPVFARW----STLT---------------------------------------------LNSTGSAVSVLGVELSTADFMVTHIHAFTIHTTVLILVKGFLYARS : [UniRef90_P58309](http://www.ebi.ac.uk/ebisearch/search.ebi?db=allebi&query=UniRef90_P58309)

[UniRef90_Q2I6P1](http://www.ebi.ac.uk/ebisearch/search.ebi?db=allebi&query=UniRef90_Q2I6P1) : -DVIALILAHKGAIISHLSWLSLFVGFHTLLVFIHNDSVFAFGEPEK-----AILIEPVFAQIIQGSSGLVFYQIGMLEPVMESHFDG-------------------------------------IGFLPIGPGDLLAHHSIALGMHVTCIILLKGSLDAR- : [UniRef90_Q2I6P1](http://www.ebi.ac.uk/ebisearch/search.ebi?db=allebi&query=UniRef90_Q2I6P1)

[UniRef90_Q52W88](http://www.ebi.ac.uk/ebisearch/search.ebi?db=allebi&query=UniRef90_Q52W88) : EDLVARILENKAVSISHLSWICLWLGFHTLALYIHNDTIVAFGEQEKQ-----ILIEPVFAQVIQESIFNIGFQFQ----------------------------------------------GIGILQMPLGPGDFLVHHAIALGLHVTMLILLKGALDAR- : [UniRef90_Q52W88](http://www.ebi.ac.uk/ebisearch/search.ebi?db=allebi&query=UniRef90_Q52W88)

[UniRef90_U6EFU9](http://www.ebi.ac.uk/ebisearch/search.ebi?db=allebi&query=UniRef90_U6EFU9) : -------IRHRDPIYSHLIWVCIAIGLHSFSLYCHNDTLEALGRPEDIFHDNSIQLKAIFAK-------------QSFLRAELQP------------------------------DIEMLDKKIIRITQELGTADFIVHHIHAFSIHVTLLIHSKGVLYARN : [UniRef90_U6EFU9](http://www.ebi.ac.uk/ebisearch/search.ebi?db=allebi&query=UniRef90_U6EFU9)

[UniRef90_V7AR62](http://www.ebi.ac.uk/ebisearch/search.ebi?db=allebi&query=UniRef90_V7AR62) : ----DRVLRHR---------------FHSFGLYIHNDTMSALGRPQDMFSDTAIHLQPIFAQWIQNTHALAPG--------------------------TTTPGVATSTSFTWGGELVAIGSKVALLPDLVSSSGSALHHA--------------------- : [UniRef90_V7AR62](http://www.ebi.ac.uk/ebisearch/search.ebi?db=allebi&query=UniRef90_V7AR62)

[UniRef90_Q8MAF4](http://www.ebi.ac.uk/ebisearch/search.ebi?db=allebi&query=UniRef90_Q8MAF4) : -NLLKRVLLHRRSILAHLNWVCIFLGTHSFGFYAHNDTMRALGRSKETFGDGSMALSPIFAKCIQGIHSVAPGN--------------------------TAPQIISISSHVFGVGTIAVKGRIAIQPLYLG------------------------------ : [UniRef90_Q8MAF4](http://www.ebi.ac.uk/ebisearch/search.ebi?db=allebi&query=UniRef90_Q8MAF4)

[UniRef90_P58383](http://www.ebi.ac.uk/ebisearch/search.ebi?db=allebi&query=UniRef90_P58383) : NDLVGKLLATKATVISTLSWITLFLGFHATGLYMHNDAMAAFGVPQKQ-----IIIEPVFAEFIQQ---------------------VFFLGTPVYGLGSAA---------------VSSTPTLSFLPIISG-GDFLVHHAIALGLHTTVLVLIKGALDS-- : [UniRef90_P58383](http://www.ebi.ac.uk/ebisearch/search.ebi?db=allebi&query=UniRef90_P58383)

[UniRef90_K4AUR4](http://www.ebi.ac.uk/ebisearch/search.ebi?db=allebi&query=UniRef90_K4AUR4) : -----------------------------FGLYIHNDTMSSLGRPQYMFPDTAIHT--CLSTWCNNNLQFNLG----------------------------------------GGDLVAMGGKVAFLPIPLGTADLLVHHIHAFTIHVTVLIFLKGILFARS : [UniRef90_K4AUR4](http://www.ebi.ac.uk/ebisearch/search.ebi?db=allebi&query=UniRef90_K4AUR4)

[UniRef90_D9IXI4](http://www.ebi.ac.uk/ebisearch/search.ebi?db=allebi&query=UniRef90_D9IXI4) : ----------------------------------HNDVVTAFGHSERQ-----ILLQPVFAQLIQTASGKNIYYPDVKLFYPLNQRDPRFLN----------------------GWLETINNLKVSPFLNVGPGDFLVHHAIALGLHTTVLILIKGAIDSR- : [UniRef90_D9IXI4](http://www.ebi.ac.uk/ebisearch/search.ebi?db=allebi&query=UniRef90_D9IXI4)

[UniRef90_U6EG22](http://www.ebi.ac.uk/ebisearch/search.ebi?db=allebi&query=UniRef90_U6EG22) : -----RIHTHKAAIIPHLSWVSLWLGFHTLAVYSHNDTCIAFNSPSKQ-----ILIEASNAQLIQQASGKALYGTI--------------------------------------NSINNYNKSFDSFIHPISPGDSYVHHAIALGLHITILILIKGGLEAR- : [UniRef90_U6EG22](http://www.ebi.ac.uk/ebisearch/search.ebi?db=allebi&query=UniRef90_U6EG22)

[UniRef90_Q52W92](http://www.ebi.ac.uk/ebisearch/search.ebi?db=allebi&query=UniRef90_Q52W92) : ------VLNHRDIICGHLIWICIALGLHSFSLYIHNDTLQAFGRPEDMFHDNSIQLKPVFSNW----------------------------------------------HPIVSFDIKILDKKVIGIGPYLGTADFIVHHIHAFTIHV-------------- : [UniRef90_Q52W92](http://www.ebi.ac.uk/ebisearch/search.ebi?db=allebi&query=UniRef90_Q52W92)

[UniRef90_D7U1J3](http://www.ebi.ac.uk/ebisearch/search.ebi?db=allebi&query=UniRef90_D7U1J3) : ---LDRVLRHRDAIISHLNWAW---------------------RPQDMFSYTVIQLQLVFAQWIQNTHALAPG--------------------------ATTPGATTSTSLTWSGDLVAVGGKVALLPIPF------------------------------- : [UniRef90_D7U1J3](http://www.ebi.ac.uk/ebisearch/search.ebi?db=allebi&query=UniRef90_D7U1J3)

[UniRef90_M5DDL9](http://www.ebi.ac.uk/ebisearch/search.ebi?db=allebi&query=UniRef90_M5DDL9) : -------------------------------------------------------LPPVFAQWIQNTHALAPG--------------------------TTAPGATTSTSLTWGGGGLVAVGGKALLPIPLGTADFLVHHIHAFTIHVTVLILLKGVLFARS : [UniRef90_M5DDL9](http://www.ebi.ac.uk/ebisearch/search.ebi?db=allebi&query=UniRef90_M5DDL9)

[UniRef90_A9X3X5](http://www.ebi.ac.uk/ebisearch/search.ebi?db=allebi&query=UniRef90_A9X3X5) : -SVIHRILELKAAILSHLSWLCLWIGFHLLCLYIHNDTVTAFGEPEKQL-----LIEPVFGQLLQGSSGKAFYSLGMIGGLNANETFGSFLLPLGPGDLLA-------------------------------------HHAISLALHVTTLVALKGSLDSR- : [UniRef90_A9X3X5](http://www.ebi.ac.uk/ebisearch/search.ebi?db=allebi&query=UniRef90_A9X3X5)

[UniRef90_V5JUZ2](http://www.ebi.ac.uk/ebisearch/search.ebi?db=allebi&query=UniRef90_V5JUZ2) : -------------------------------------------------------------QWIQAAQGKTLYGFDFLLSSSSSNATAASQSLWLP------------------GWLEAINNNQNSLFLTIGPGDFLVHHAIALGLHTTTLILVKGALDAR- : [UniRef90_V5JUZ2](http://www.ebi.ac.uk/ebisearch/search.ebi?db=allebi&query=UniRef90_V5JUZ2)

[UniRef90_Q52W91](http://www.ebi.ac.uk/ebisearch/search.ebi?db=allebi&query=UniRef90_Q52W91) : NSIVQQLLGHRDIIMGHLIWVTIALGLHAFGIYIHNDTLQALGRPEDIFSDNSIQLKPLFAVWVQSLPSLFLLNALSGDAAVTGIPGFGLEVLDGKVVTMT------------------------------------------------------------- : [UniRef90_Q52W91](http://www.ebi.ac.uk/ebisearch/search.ebi?db=allebi&query=UniRef90_Q52W91)

: 1---------11--------21--------31--------41--------51--------61--------71--------81--------91--------101-------111-------121-------131-------141-------151-------16 :

OrigSeq : NNVLDRVLRHRDAIISHLAWVCQFLGFHSFAMYCHNDTMRAFGRPQDMFSDTGIQLQPVFAQWLQHIHTMTIGNPSLQVAAPLGHAFGGLRNLELTGLGTAAPNLHDPVSYAFGGGVVAVGGKVAMMPITLGTADFLIHHIHAFTIHVTVLVLLKGVLFARS : OrigSeq

Jnet : --HHHHHHHHHHHHHHHHHHHHHHHHHHHHHHHHHHHHHHH----------------HHHHHHHHHHHHHH---------------------------------------EEE---HEHH-----EE--------HHHHHHHHHHHHHHHHHHHHH------ : Jnet

jhmm : -HHHHHHHHHHHHHHHHHHHHHHHHHHHHHHHHHHHHHHHHH---HHHH----EEEHHHHHHHHHHHHHHHH---EEE----------HHH-------HHHHHHHH----EEE---HHHHH---EEEEE------HHHHHHHHHHHHHHHHHHHH------- : jhmm

jpssm : --HHHHHHHHHHHHH---HHHHHHHHHHHHHHHHHHHHH-------------------HHHHHHHHHHHHH----------------------------------------EE---EEE----------------HHHHHHHHHHHHHHHHHHHHH------ : jpssm

Lupas 14 : ------------------------------------------------------------------------------------------------------------------------------------------------------------------ : Lupas 14

Lupas 21 : ------------------------------------------------------------------------------------------------------------------------------------------------------------------ : Lupas 21

Lupas 28 : ------------------------------------------------------------------------------------------------------------------------------------------------------------------ : Lupas 28

Jnet_25 : --BB--BB-B--BBBBBBBBBBB-BBBBBBBBBBBBBBB-BB-----BB---BB-B-BBBB-BB-BBB--BBB-B-BBBBB--B-BBB-B--B-B-B-BBBBBBBB---BBBBBBBB--B---B-BBBB-B-BBBBBBBBBBBB-BBBBBBBBBBBBB-B-- : Jnet_25

Jnet_5 : ------B------BBB----B-B-B---BBBBBB---BB-B-----------------BBB-BB--------------------------------------------------------------B--------BBB--B-B----BBBBB-B-B------ : Jnet_5

Jnet_0 : ---------------------------------------------------------------B------------------------------------------------------------------------------B-----B--B---------- : Jnet_0

Jnet Rel : 926899999767750333179999999987578877441202777332377772220378999999987301677101777777777722377777771112001277640305650010267721102667763789999999999999998730158999 : Jnet Rel

**Notes**

**Key:**

Colour code for alignment:

Blue - Complete identity at a position

Shades of red - The more red a position is, the higher the level of

conservation of chemical properties of the amino acids

Jnet - Final secondary structure prediction for query

jalign - Jnet alignment prediction

jhmm - Jnet hmm profile prediction

jpssm - Jnet PSIBLAST pssm profile prediction

Lupas - Lupas Coil prediction (window size of 14, 21 and 28)

Note on coiled coil predictions - = less than 50% probability

c = between 50% and 90% probability

C = greater than 90% probability

Jnet_25 - Jnet prediction of burial, less than 25% solvent accessibility

Jnet_5 - Jnet prediction of burial, less than 5% exposure

Jnet_0 - Jnet prediction of burial, 0% exposure

Jnet Rel - Jnet reliability of prediction accuracy, ranges from 0 to 9, bigger is better.
